# Ornithine lipids from *Akkermansia muciniphila* are dynamically modulated in colitis and shape macrophage inflammatory responses

**DOI:** 10.1080/19490976.2025.2601376

**Published:** 2025-12-16

**Authors:** Habiba Selmi, Alesia Walker, Laurence Balas, Marianna Lucio, Markus Klotz, Aicha Jeridi, Anna G. Burrichter, Devon Conti, Lorenzo Chaffringeon, Brice Beinsteiner, Marion Jasnin, Nicolas Vanthuyne, Thierry Durand, Ali Önder Yildirim, Bärbel Stecher, Laurent Debarbieux, Philippe Schmitt-Kopplin

**Affiliations:** a Research Unit Analytical BioGeoChemistry, Helmholtz Zentrum München, Neuherberg, Germany; b Institut des Biomolécules Max Mousseron, IBMM, Université de Montpellier, CNRS, ENSCM, Montpellier, France; c Institute of Lung Health and Immunity (LHI), Comprehensive Pneumology Center (CPC), Helmholtz Munich, Member of the German Center for Lung Research (DZL), Munich, Germany; d Max von Pettenkofer Institute of Hygiene and Medical Microbiology, Faculty of Medicine, LMU Munich, Germany; e Chair of Intestinal Microbiome, Technical University of Munich, Freising, Germany; f Institut Pasteur, Université Paris Cité,CNRS UMR6047, Bacteriophage Bacterium Host, Paris, France; g Sorbonne Université, Collège Doctoral, Paris, France; h Helmholtz Pioneer Campus, Helmholtz Zentrum München, Neuherberg, Germany; i Aix Marseille Univ., CNRS, Centrale Med, FSCM, Chiropole, Marseille, France; j Institute of Experimental Pneumology, LMU University Hospital, Ludwig-Maximilian’s University, Munich, Germany; k German Center for Infection Research (DZIF), partner site LMU Munich, Germany; l Chair of Analytical Food Chemistry, Technical University of Munich, Freising, Germany

**Keywords:** Ornithine lipids, *akkermansia muciniphila*, *citrobacter rodentium*, Oligo-Mouse-Microbiota, ulcerative colitis, IL-1β

## Abstract

The gut microbiota is a key modulator of host immunity, in part through the production of structurally diverse and largely still uncharacterized bacterial lipids and metabolites with potential immunoregulatory properties. Using a gnotobiotic Oligo-Mouse-Microbiota (OMM^12^) mouse model infected with the *Citrobacter rodentium* pathogen, we investigated metabolomic changes associated with colitis. Untargeted metabolomics revealed an accumulation of host-derived lipids in the inflamed colon, while several bacterial lipid classes, including sphingolipids, glycerophospholipids, and fatty acyls were depleted. Among the bacterial lipids, ornithine-containing lipids (OLs) produced by *Akkermansia muciniphila* were significantly reduced during inflammation. Isolation, structural characterization, and chemical synthesis revealed OL 16:0/15:0 as a membrane-associated lipid from *A. muciniphila*. This lipid contains an *L*-ornithine head group, with its *α*-amino group forming an amide bond with 3(*R*)-hydroxypalmitic acid, while the 3(*R*)-hydroxyl position is esterified with pentadecanoic acid. Functional studies showed that macrophages internalize and partially metabolize OL 16:0/15:0 into *N*^*α*^-(3-hydroxypalmitoyl)-*L*-ornithine and 3(*R*)-hydroxypalmitic acid. In LPS-stimulated macrophages, a 1:1 mixture of OL diastereomers (*3R,S + 3S,S*) reduced *Il6* and *Il1b* gene expression and decreased IL-6 secretion, without triggering IL-1β release. Interestingly, this diastereomeric mixture exhibited an opposite effect to the natural (*3R,S*)-epimer, which selectively promoted IL-1β secretion in LPS-primed macrophages. These results uncover a possible stereoselective modulation of IL-1β production by bacterial OLs. Overall, OL 16:0/15:0 is dynamically regulated during inflammation and may play a role in the immunomodulation of host–microbiota interactions.

## Introduction

The enteric pathogen *Citrobacter rodentium* serves as a well-established murine model to study infections caused by human enteropathogenic and enterohemorrhagic *Escherichia coli*. *C. rodentium* closely mimics in mice the infection mechanisms of enteropathogens by colonizing the gut mucosa and inducing hallmark attaching and effacing lesions.^1^ Moreover, this murine infection model has been widely used to investigate the pathophysiology of human intestinal diseases, particularly ulcerative colitis (UC), a prevalent form of inflammatory bowel disease (IBD).^2^ Crohn’s disease and UC are two common forms of IBD and are chronic, relapsing conditions of the gastrointestinal tract characterized by epithelial barrier disruption, dysregulated mucosal immunity, and complex interactions between gut microbes and the host immune system.^3^ While the etiology of IBD is multifactorial, including genetic susceptibility, immune dysregulation, and environmental influences, growing evidence suggests that impaired host sensing of microbial signals, along with alterations in gut microbiota composition and function, are implicated in disease pathogenesis.^4^^,^^5^ Among the key mediators of host–microbiota interaction, microbial-derived lipids and metabolites have emerged as crucial molecular signals.^6^ In particular, recent metabolomics and lipidomics studies have highlighted their role in regulating immune responses and contributing to inflammation.^7^ This underscores their potential as drivers or modulators of IBD progression.^8^ The *C. rodentium* mouse model has been instrumental in elucidating the links between bacterial infections, mucosal immune responses, and gut inflammation by replicating key pathogenetic triggers of UC,^9–11^ with several reported studies highlighting the immunometabolic pathways activated during infection.^12^^,^^13^

In UC, metabolomics analysis has revealed significant molecular shifts, particularly in lipid metabolism.^14^^,^^15^ These lipidomic changes are recognized for their key role in modulating host-microbiome interactions and immune responses, thereby contributing to UC pathogenesis.^16^^,^^17^ In particular, high-resolution mass spectrometry-based lipidomics has identified bioactive lipid mediators, such as *ω*-3 fatty acids, which exhibit immunomodulatory properties in UC.^18^^,^^19^ While host-derived lipids have been extensively studied in inflammatory diseases,^20^ the role of microbial lipids in immune regulation under disease conditions remains largely unexplored. Among bacterial lipid mediators, sphingolipids have been shown to influence host lipid metabolism, as their depletion disrupts microbiome-host interactions and induces a compensatory increase in host-derived sphingolipids.^16^ In addition, cardiolipins from *Muribaculum intestinale* and phospholipids from *Akkermansia muciniphila* have been implicated in gut homeostasis and immune modulation.^21^^,^^22^

*A. muciniphila*, a mucin-degrading gut commensal, has emerged as a key microbial species implicated in maintaining gut homeostasis and modulating inflammatory responses, either directly or through its structurally unique lipids and microbial metabolites.^23–26^ It has been demonstrated to enhance epithelial barrier function, regulate mucosal immune function, and influence host metabolism in extraintestinal compartments.^27^ In a recent study of experimental colitis, dietary palmitoleic acid was shown to enrich *A. muciniphila* abundance and enhance the efficacy of anti-TNF therapy, supporting its role in promoting gut immune homeostasis.^28^ Moreover, structural analysis of lipooligosaccharide derived from *A. muciniphila* cell membrane revealed a unique composition that activates both TLR2 and TLR4 signaling, with a marked bias toward anti-inflammatory TLR2-mediated responses and induction of IL-10 expression.^24^

In the present study, we investigate lipid alterations associated with gut inflammation by employing a gnotobiotic mouse model stably colonized with the Oligo-Mouse-Microbiota (OMM^12^) synthetic community and infected with *C. rodentium.*^29^ This defined OMM^12^ consortium harbors representatives of the major bacterial phyla typically found in the murine gastrointestinal tract, Firmicutes, Bacteroidetes, Verrucomicrobia, Actinobacteria, and Proteobacteria.^30^ The model establishes a stable and well-characterized microbial community, providing a robust tool for studying host–microbe interactions and enabling comprehensive profiling of host and microbiome lipidomes and metabolomes throughout disease progression. High-throughput LC-MS-based metabolomics, combined with advanced multivariate statistical analyzes, revealed a significant increase in host-derived lipids and reduction in microbial lipids within the colon at peak inflammation. Among the altered lipid classes, ornithine-containing lipids (OLs) from *A. muciniphila* displayed dynamic regulation. To further elucidate their role, we isolated, structurally characterized and chemically synthesized OL 16:0/15:0, found in *A. muciniphila* cell membrane and outer membrane vesicles (OMV). Functional assays in macrophages revealed a stereoselective effect on IL-1β secretion.

## Methods

### Ethics approval statement

A total of forty OMM^12^ and six axenic mice (C57BL/6J), 7-9 weeks old, including both males and females, were obtained from Institut Pasteur (Paris, France). Animals were housed in isocages and maintained on a standard diet. Ethical approval was obtained from the Animal Experimentation Committee of Institut Pasteur, with authorization from the French Ministry of Research (APAFIS#26874-2020081309052574 v1).

### Animal experiments

OMM^12^ mice received by oral gavage 200 μL of *C. rodentium* suspension (5 × 10^7^ CFU) in phosphate-buffered saline (PBS). Mice from two independent experiments were sacrificed at three time points: pre-infection (day 0, *n* = 13), day 10 p.i. (*n* = 10), and day 20 p.i. (*n* = 17) to collect ileal and colonic sections. Three mice from each group were dedicated to intestinal tissue characterization (fixation in Carnoy followed by HE staining). Intestinal contents were obtained by gently squeezing the intestine with the back of a scalpel, weighed, snap-frozen in liquid nitrogen, and stored at −80 °C for further analysis. Before sacrifice, fecal pellets were collected at different time points to monitor *C. rodentium* load by plating on Drigalski agar and to perform Lipocalin-2 assays.

### Lipocalin-2 quantification

Fecal lipocalin-2 levels were measured from frozen supernatant of fecal pellets resuspended in PBS, using an enzyme-linked immunosorbent assay (ELISA) kit (DY1857, R&D Systems, Minneapolis, USA) according to the manufacturer's instructions.

### Preparation of bacterial and murine intestinal samples

Bacterial cultures (growth conditions detailed in Supplementary Note S1) were harvested at early stationary phase. Cells were pelleted by centrifugation at 14,000 × *g* for 15 min at 4 °C, and supernatants were collected separately. Pellets were snap-frozen in liquid nitrogen. For LC-MS analysis, bacterial pellets were washed twice with 1 mL cold sterile PBS and centrifuged to remove residual medium.

Gut contents from OMM^12^ and germ-free mice, as well as bacterial pellets, were processed using the same extraction protocol. Samples were resuspended in 1 mL of pre-chilled methanol (MeOH; -20 °C, LiChrosolv, Supelco, Merck, Darmstadt, Germany), thoroughly mixed, and transferred into sterile ceramic bead tubes (NucleoSpin® Bead Tubes, Macherey-Nagel, Dueren, Germany). Homogenization was performed using a Precellys® Evolution Homogenizer (Bertin Corp., Rockville, MD, USA) at 4,500 rpm for three 40-second cycles with 2-second pauses between cycles. Colonic tissue samples from germ-free mice were also extracted. Tissue preparation followed the same procedure as for colonic contents, with the addition of a second homogenization step (6,800 rpm for three 60-second cycles, with 3-second pauses) to ensure complete tissue disruption.

Following homogenization, all samples were centrifuged at 21,000 × *g* for 10 minutes at 4 °C. The upper phase (900 µL) was collected into safety reaction tubes (Eppendorf, Hamburg, Germany). A 50 µL aliquot of the supernatant was evaporated at 40 °C using a SpeedVac concentrator (Savant SPD121P, Fisher Scientific, Waltham, MA, USA) and reconstituted in 10% acetonitrile (ACN, Merck KGaA, LiChrosolv®, Darmstadt, Germany) spiked with a mixture of deuterated standards (d4-cholic acid and d4-glycodeoxycholic acid, 0.01 mg/mL in MeOH). Samples were then prepared for non-targeted LC-MS analysis.

### OMVs isolation and cryo-EM data acquisition

Isolation of *A. muciniphila* vesicles was performed as previously described.^31^ OMVs were purified from bacterial cultures using sequential centrifugation and filtration steps. Final OMV pellets were resuspended in sterile PBS and prepared for cryo-EM and LC-MS analyzes. For cryo-EM analysis, OMVs were diluted in sterile PBS and applied to holey R 1.2/1.3 carbon 200 mesh copper grids (Quantifoil). Grids were treated by glow discharge (at 4 mA for 30 s), blotted and cryo-cooled in liquid ethane using a Vitrobot Mark IV system (Thermo Fisher) with the chamber operating at 95% humidity and at 4 °C. The micrographs were acquired using the EPU software on the Krios G4 equipped with a cold-FEG operated at 300 kV and equipped with a Falcon IVi camera and a Selectris X energy filter (Thermo Fisher). Micrographs were captured at 105,000 × magnification.

### LC-MS/MS analysis of OMM^12^ colonic and ileal samples

LC-MS/MS analysis was performed on an ultra-high performance liquid chromatography (UHPLC) system (ExionLC, AB Sciex LLC, Framingham, MA, USA) coupled to a quadrupole time-of-flight mass spectrometer (X500 QTOF MS, AB Sciex LLC, Framingham, MA, USA) with a DuoSpray ESI source. Before each analysis, mass calibration was conducted in both ionization modes using a calibration delivery system (ESI Positive/Negative Calibration Solution, AB Sciex Germany GmbH, Darmstadt, Germany), with automatic calibration of the QTOF performed after every tenth injection. The MS/MS data were acquired in information-dependent acquisition (IDA) mode, with full scan coverage from 65 to 1000 Da in both positive and negative ESI modes. Chromatographic separation was performed using an ACQUITY BEH C8 column with gradient elution, as reported previously.^32^ The autosampler was maintained at 4 °C, the column was heated to 60 °C, and the injection volume was 5 µL with a flow rate of 0.35 mL/min. A pooled quality control (QC) sample, consisting of aliquots from all biological samples, was injected every tenth run. Samples were analyzed in randomized order. Full details of TOF MS and MS/MS parameters are provided in Supplementary Table S1.

### Data processing and feature identification

Non-targeted peak picking was performed using Genedata Expressionist Refiner MS 15.0.7 (Genedata GmbH, Basel, Switzerland). Raw data files (.wiff2) were processed using a multi-step workflow, including chemical noise subtraction, gridding, retention time restriction, peak detection, blank peak filtering, chromatogram isotope clustering, consolidation, and MS/MS peak detection. Features were annotated by comparing mass-to-charge ratios (*m*/*z*, ± 0.005 Da) and retention times (RT, ± 0.07 min) against an in-house standard library and by searching the Human Metabolome Database (HMDB) with an *m*/*z* and RT tolerances of 0.005 Dalton and ± 0.05 min, respectively. A data matrix was generated, containing features defined by unique cluster numbers (*m*/*z* and RT) with corresponding maximum intensity values for each sample. Intensities were normalized to sample weight and internal standards using the Analyst module in Genedata Expressionist. Chromatogram visualization was achieved in MZmine 3.9.0, after converting.wiff2 files to MzML format with ProteoWizard msConvert 3.0.20342. MS/MS spectra were matched to spectral libraries using MS PepSearch (released 22/02/2019), with libraries downloaded from both the MassBank of North America (MoNA) and the MSDIAL-TandemMassSpectralAtlas-VS69. Matching was performed with precursor ion tolerances of 0.01 *m*/*z* and MS peak tolerances of 0.05 *m*/*z*. Molecular feature classification and prediction were conducted using the CANOPUS tool in Sirius 5.8.5.^33–35^ Features were considered annotated if they matched a spectral library with a dot product score greater than 700, or if they were classified by CANOPUS with a classification probability exceeding 0.7. Targeted peak picking of .wiff2 data was performed using Sciex OS Analytics 3.0 (AB Sciex LLC, Framingham, MA, USA).

### OL liposome preparation and cryo-EM data acquisition

Liposomes of synthetic OL 16:0/15:0 were prepared following established protocols,^36^ with slight modifications. Briefly, stock solutions of OLs (as a diastereomeric mixture and (3*R,S*)-epimer) were prepared at 1.5 mg/mL in chloroform (CHCl₃). Lipid films were formed by subjecting the solutions to three evaporation cycles under nitrogen flow, followed by drying in a SpeedVac system. The resulting films were stored at −20 °C until further use. On the day of treatment, liposomes were freshly prepared by solubilizing lipid films in sterile and filtered 10 mM HEPES to achieve a final concentration of 4 mg/mL. The suspension was heated at 70 °C for 20 minutes, followed by two sonication cycles (5 seconds each, 5-second intervals) at 35 kHz. This step yielded white liposome suspensions, which were further diluted with filtered HEPES to a final stock concentration of 1.5 mg/mL. Intermediate OL solutions were prepared for subsequent bone marrow-derived macrophages (BMDM) experiments.

For cryo-EM analysis, OL samples were applied to holey R 3.5/1 carbon 200-mesh copper grids (Quantifoil) covered with a homemade 3 nm-thick continuous carbon film. Grids were glow-discharged (4 mA, 10 s), blotted, and cryo-cooled into liquid ethane using a Vitrobot Mark IV (Thermo Fisher) operated at 95% humidity and 22 °C. Micrographs were acquired using EPU software on a Krios G4 electron microscope equipped with a cold-FEG (operated at 300 kV), a Falcon IVi camera, and a Selectris X energy filter (Thermo Fisher). Micrographs were captured at a magnification of 105,000 × . In one experiment, 4 grids were prepared. During screening, 19 example micrographs were acquired at high magnification.

### BMDMs cytokine assays and metabolic profiling of OL 16:0/15:0

Bone marrow cells were harvested from the femurs and tibias of C57BL/6J mice and cultured in 6-well plates at a density of 3 × 10⁶ cells per well in 3 mL of complete medium, comprising RPMI 1640 supplemented with 10% fetal bovine serum (FBS), 1% penicillin-streptomycin, and 50 μM 2-mercaptoethanol. To achieve macrophage differentiation, recombinant murine macrophage colony-stimulating factor (M-CSF; ImmunoTool, #12343115) was added at 20 ng/mL. Cultures were maintained at 37 °C in a humidified atmosphere containing 5% CO₂, with half of the medium replaced on days 3 and 6. On day 7, the medium was fully refreshed, and the M-CSF concentration was reduced to 10 ng/mL. On day 8, differentiated macrophages were used for OL treatments and cytokine quantification following LPS stimulation or other follow-up experiments. BMDMs were pre-treated with OL liposome suspensions at 50, 100, and 150 μg/mL, for 1 hour. After pre-treatment, cells were stimulated with LPS (10 ng/mL) for 6 hours. Controls included HEPES buffer (10 mM) ± LPS.

Cytokine gene expression and secretion were assessed by qPCR and ELISA (Bio-Techne kits), respectively. Data were analyzed by one-way ANOVA with Dunnett’s post hoc test using GraphPad Prism v10.2.3 (p* ≤ 0.05). ELISA was performed to quantify IL-1β levels in cell culture supernatant using the Mouse-IL1beta/IL-1F2 DuoSet ELISA from Bio-Techne, following the manufacturer’s instructions.

For metabolic profiling, BMDMs were treated with OL 16:0/15:0 (10 or 25 μg/mL), and culture supernatants were collected at 0, 18, 24, and 48 h. Control samples, consisting of OL solutions in culture medium without cells, were processed in parallel to assess non-cellular OL degradation. Supernatant samples were prepared using established extraction protocols and analyzed using a C8 RP method in ESI(–) for metabolic profiling. Metabolites with ≥3-fold change (OL vs. control) were considered significant.

### Statistical analysis

Univariate and multivariate statistical analyzes were employed to identify features associated with *C. rodentium*-induced colitis. Orthogonal Partial Least Squares Discriminant Analysis (OPLS-DA) was performed to compare OMM^12^ mice at baseline, 10 d, and 20 d p.i., as well as to compare OMM^12^ with axenic mice. Sex was not included as a covariate in the multivariate or univariate statistical models because no significant sex-dependent effects were observed in the preliminary analyzes. Features with MS/MS data were filtered based on occurrence (≥10%) and scaled to unit variance. OPLS-DA models were assessed for overfitting using cross-validation ANOVA (CV-ANOVA), with significance set at p* ≤ 0.05. Model fit (R²Y(cum)) and predictive power (Q²(cum)) were calculated for each model. Features with a variable importance in projection (VIP) score >1 were considered significant. Statistical significance was further determined using pairwise Welch's *t*-tests with Benjamini–Hochberg correction (*p ≤ 0.05). Only significant features were classified according to their putative origin as either host- or bacteria-derived metabolites. Host and bacterial features were classified as significant if they had a VIP score >1 and a fold change |log₂FC| ≥ 2.3. Heatmaps were generated for data visualization following Z-score normalization across time points. Statistical analyzes were conducted using SIMCA 13.03 (Umetrics) and RStudio (version 2023.12.1).

## Results

### *C. rodentium*-induced colitis alters colonic lipid profiles

**Figure 1.**
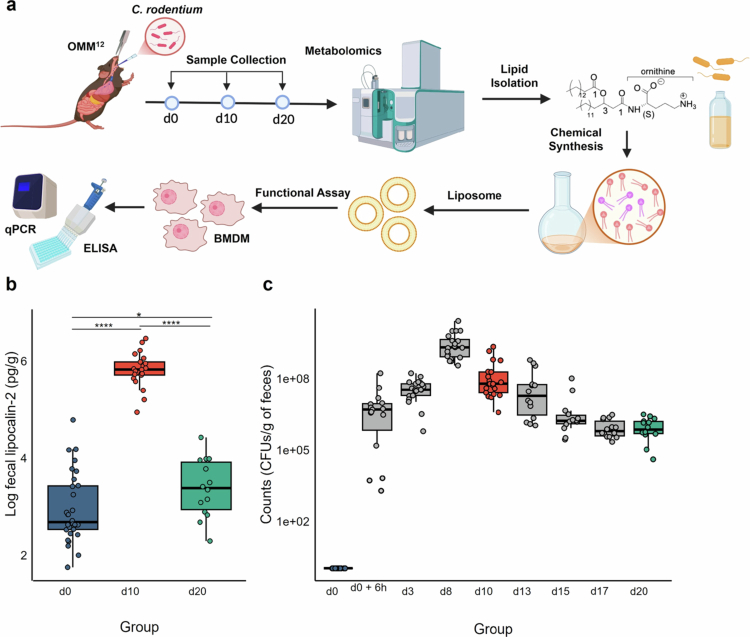
Experimental design and inflammatory response in a *C. rodentium*-induced colitis OMM^12^ mouse model. (a) Schematic overview of the experimental workflow, illustrating the main steps of the study. OMM^12^ mice were orally gavaged with *C. rodentium* at day 0 (d0). Colonic and ileal content samples were collected at baseline (d0, *n* = 10), 10 days (d10, *n* = 7), and 20 days (d20, *n* = 14) p.i. for LC-MS-based metabolomic profiling. Lipid isolation, chemical synthesis and functional assays were performed to further characterize lipids identified from murine samples. (b) Box plots show log-transformed lipocalin-2 values at d0, d10, and d20, alongside with *C. rodentium* fecal loads (CFUs/g of feces) over time (c). Statistical significance for lipocalin-2 levels was assessed using a pairwise Welch *t*-test with Benjamini-Hochberg adjustment (*p ≤ 0.05, **p ≤ 0.01, ***p ≤ 0.001).

To investigate metabolic changes associated with colitis, we infected OMM^12^ mice with *C. rodentium* and collected ileal and colonic contents at baseline (d0), 10 d (d10), and 20 d (d20) post-infection (p.i.) for LC-MS-based metabolomics analysis, chemical and functional characterization of key compounds (Figure 1a). At day 10 p.i., we observed a significant increase in fecal lipocalin-2 level, a biomarker of intestinal inflammation^37^ (Figure 1b). This inflammatory peak followed the highest burden of *C. rodentium*, which occurred at day 8 (Figure 1c). Accordingly, colonic sections collected at day 10 p.i. displayed marked epithelial damage characterized by crypt deformation and dense immune cell infiltration, consistent with acute inflammation. By day 20 p.i., colonic architecture had largely recovered, with restoration of epithelial and crypt structures and a pronounced reduction in inflammatory cell infiltration (Supplementary Figure S1).

**Figure 2.**
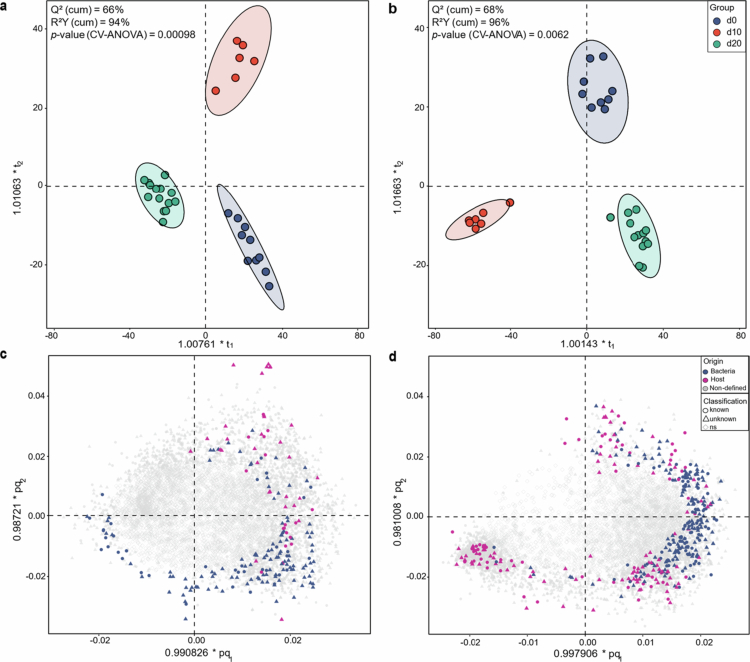
Distinct metabolic profiles in *C. rodentium*-induced colitis. OPLS-DA score plots of ileal (a) and colonic (b) metabolomic profiles, showing samples clustered into three distinct groups corresponding to different time points: day 0 (d0, blue), day 10 (d10, red), and day 20 (d20, green) post-infection. The distances between points reflect differences in global metabolomic profiles: samples closer together have more similar profiles, while greater distances indicate larger metabolic divergence. The clear separation between time points indicates dynamic shifts in metabolic states during infection. Ellipses represent 95% confidence intervals for each group. (c, d) OPLS-DA loading plots for ileal (c) and colonic (d) groups, illustrating the distribution of bacterial-derived (blue), host-derived (purple), and non-defined (gray) statistically significant features (VIP >1). Circles (◯) mark identified significant features, triangles (△) represent unknown significant features, and diamonds (◇) correspond to non-significant features (ns) (VIP ≤1).

Consistent with previous metabolomic studies of colitis-associated metabolic shifts,^38^ our analysis revealed substantial lipidomic and metabolomic alterations. Orthogonal Partial Least Squares Discriminant Analysis (OPLS-DA) identified distinct metabolic profiles between control (d0) and infected mice (d10, d20), with nearly 2,500 metabolite features exhibiting statistically significant changes (Figure 2a, b). Notably, ileal metabolic patterns remained relatively stable between baseline (d0) and peak inflammation (d10) (Figure 2a). In contrast, colonic profiles diverged significantly at d10, likely reflecting the predominant site of infection of *C. rodentium* in the distal colon, where severe mucosal damage occurs.^39^ By d20, colonic metabolic profiles had partially reverted toward baseline, indicating the onset of a recovery phase (Figure 2b).

We compared non-infected germ-free (GF) and OMM^12^ mice to distinguish host- and microbiome-derived metabolic alterations. This analysis revealed distinct contributions of bacterial and host metabolism to colonic features (Figure 2d; Supplementary Figure S2). Notably, a substantial number of significant features were identified as lipids (Supplementary Figure S3), reflecting a pronounced lipidomic shift at peak inflammation, with host-derived lipids enriched and bacterial lipids depleted. These results indicate that *C. rodentium*-induced colitis drives metabolic reprogramming, characterized by host lipid accumulation and bacterial lipid depletion, reflecting functional adaptations in both the host and microbiome.^40^

### Host and microbial lipid abundances define a metabolic feature of *C. rodentium*-induced colitis

**Figure 3.**
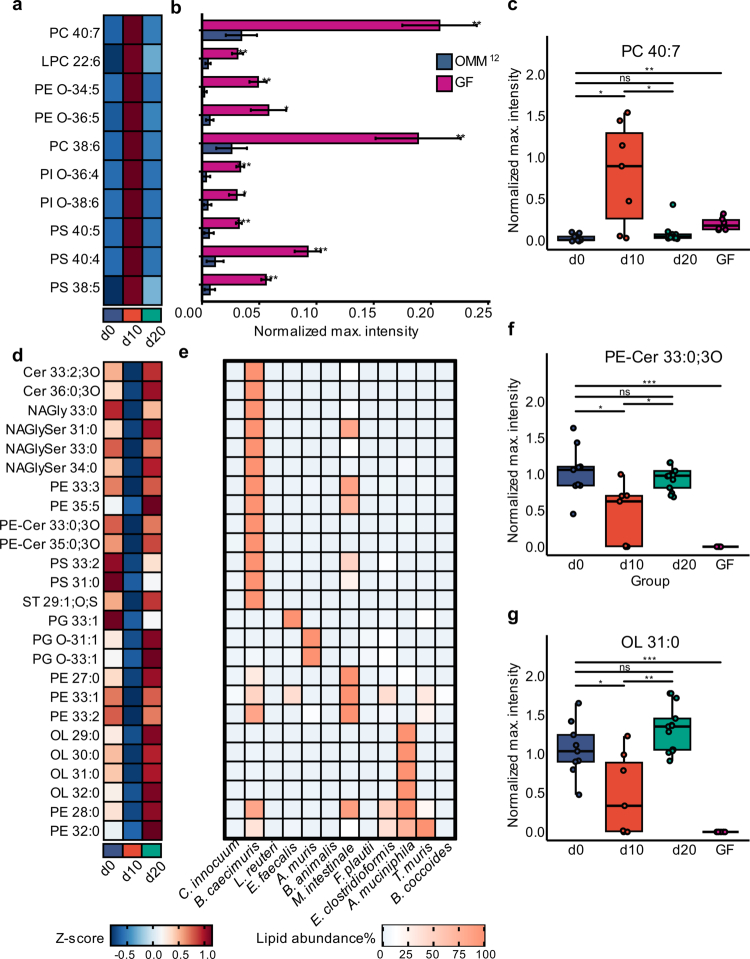
Host- and microbial-derived lipid alterations during *C. rodentium*-induced colonic inflammation. (a) Heatmap of representative host-derived lipid species in the colon showing significant changes across baseline d0, d10, and d20 p.i. Colors indicate relative abundance changes, with red denoting increased and blue decreased levels. Data represent the means of normalized maximum intensities. Lipid intensities were normalized to internal standards and sample weight before averaging within groups, then Z-score transformed and displayed as heatmaps. (b) Abundance of different lipid species in colonic samples from OMM^12^ and GF mice, demonstrating their predominantly host-derived origin. (c) Boxplot of normalized maximum peak intensities for the host-derived lipid species PC 40:7, showing a significant increase at day 10 post-infection (d10 p.i.). (d) Top 25 bacterial lipid species ranked by their bacterial origin, showing temporal changes in abundance throughout different inflammation stages in colon. (e) Lipid profiles of the 12 individual bacterial strains of the OMM^12^ community. Peak intensities are expressed as percentages, normalized to the most abundant peak (set at 100%). (f, g) Boxplots showing normalized maximum peak intensities of two bacterial lipid species, PE-Cer 33:0;3O (f) and OL 31:0 (g), across distinct stages of inflammation and in GF samples. Statistical analyzes for panels (b, c, f, and g) were conducted using pairwise Welch’s *t*-test, with *p*-values adjusted for multiple comparisons using the Benjamini–Hochberg method to control the false discovery rate (*p ≤ 0.05, **p ≤ 0.01, ***p ≤ 0.001). Boxplots show the interquartile range (IQR) and median, with whiskers extending to data points within 1.5 times the IQR from the first and third quartiles.

We employed high-resolution LC-MS analysis to investigate host and microbial lipid alterations in the colon during inflammation. Distinct shifts were observed across multiple host lipid classes, including phosphatidylcholines (PC), phosphatidylethanolamines (PE), phosphatidylinositols (PI), and phosphatidylserines (PS), along with their lysophospholipid counterparts (Figure 3a). Several lipid species were strongly increased on day 10 p.i., subsequently returning to baseline levels at d20, reflecting the pattern of fecal lipocalin-2 levels (Figure 1b). These lipids were markedly more abundant in colonic content samples from GF mice compared to OMM^12^ mice (Figure 3b) and detected in colonic organ samples from GF mice (Examples in Supplementary Figure S4), indicating a predominantly host-derived origin.^41^^,^^42^ Among these, we observed at d10 p.i. higher levels of ether-linked PEs and ester-linked PCs compared to d0 and d20 including PC 40:7 (Figure 3c). These lipid changes may reflect immune activation during *C. rodentium* infection, as ether-linked phospholipids have been detected in immune cells such as macrophages.^43^^,^^44^ They may also result from tissue damage and membrane degradation associated with inflammation. On d10, host-derived lipids showed a marked increase, while bacterial lipids exhibited a strong reduction in the colon section, followed by a partial recovery on day 20 (Figure 3a,d). Notably, all lipid species showed similar abundance patterns across conditions (Figure 3d), suggesting a class-wide response.

A comparable trend was observed for ceramide phosphoethanolamine (PE-Cer), *N*-acyl glycine-serine (NAGlySer) lipids and OL, indicating that inflammation is associated with consistent shifts in the abundance of multiple bacterial lipid classes. In murine colonic samples, four predominant OL species were detected (Supplementary Figure S5).

We aimed to profile lipids by LC-MS derived from OMM^12^ bacterial communities grown *in vitro* to further investigate bacterial lipids associated with inflammation (Supplementary Figure S6a). Furthermore, targeted lipidomics of bacterial monocultures was performed to assign specific lipid signatures to individual taxa contributing to the lipidomic shifts observed during colitis (Figure 3d). This analysis uncovered diverse classes of bacterial lipids, including PEs, fatty amides such as *N*-acylglycine (NAGly), NAGlySer, OLs, and sphingolipids, mostly dihydroceramides (Cer) and PE-Cer (Examples in Supplementary Figure S6b).

Lipidomic profiling of individual microbial strains showed distinct lipid signatures, particularly in *Bacteroides caecimuris* and *A. muciniphila* (Figure 3f,g). For example, PE-Cer 33:0;3O, specific to *Bacteroides caecimuris,* showed a marked decrease at d10 p.i. (Figure 3f). Four OLs, uniquely produced by *A. muciniphila*, were significantly reduced at d10 p.i., including OL 31:0 (Figure 3g).

### Structural elucidation of OLs isolated from *A. muciniphila*

Next, we focused on OLs, a lipid class that emerged as one of the most significantly modulated microbial lipid species in this study (Figure 3d,g). *A. muciniphila* was identified as the primary producer of these lipids. We selected OLs based on their taxonomic specificity and strong association with *A. muciniphila*, a member of the OMM^12^ consortium with known immunomodulatory properties,^23^ rather than their abundance in intestinal content or in the *in vitro* system.

**Figure 4.**
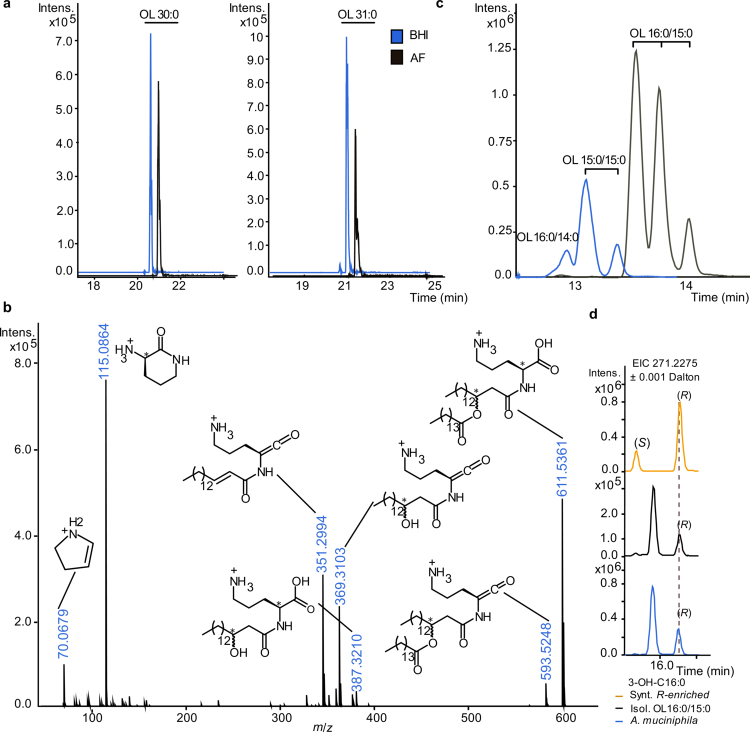
Structural characterization of OLs from the *A. muciniphila* cell pellet. (a) Extracted ion chromatograms (EICs) of two of the most abundant OL species in *A. muciniphila*, OL 30:0 (*m*/*z* 595.5057 ± 0.001 Dalton) and OL 31:0 (*m*/*z* 609.5215 ± 0.001 Dalton), from *A. muciniphila* cultured in brain heart infusion (BHI) medium or anaerobic rich (AF) medium. Separation was performed using a reversed-phase (RP) C8 column in electrospray ionization in negative mode (ESI(-)). (b) MS/MS spectrum of OL 16:0/15:0 acquired in ESI(+), shows key fragment ions with their annotated structures. (c) EICs of the most abundant OL species: OL 15:0/15:0 (*m*/*z* 597.5215 ± 0.001 Dalton) and OL 16:0/15:0 (*m*/*z* 611.5361 ± 0.001 Dalton). Separation was achieved using a C18 column in ESI(+) mode. (d) Chiral separation and absolute configuration assignment of 3-OH-C16:0. Chiral separation and stereochemical assignment of 3-OH-C16:0 were conducted on hydrolysates from OL 16:0/15:0 fractions and *A. muciniphila* pellet extracts using a Chiralpak IA-U column. The (*R*) configuration of straight-chain 3-OH-C16:0 in biological samples was confirmed by comparison with a synthetic enantiomerically enriched 3(*R*)-OH-C16:0 standard (80% (*R*) and 20% (*S*) diastereomers, defined by peak area), analyzed *via* LC-MS/MS in ESI(-) mode. Branched 3-OH-C16:0 species have been detected in *A. muciniphila*-derived OL 16:0/15:0, although their absolute stereochemistry remains undefined.

To characterize the structural diversity of OLs, *A. muciniphila* was cultured in two media with distinct nutrient compositions (Supplementary Note S1). High-resolution LC-MS combined with tandem mass spectrometry (MS/MS) identified four predominant OLs: OL 29:0, OL 30:0, OL 31:0, and OL 32:0, with representative examples of the most abundant species in the *A. muciniphila* cell membrane (Figure 4a).

Moreover, we annotated a wide range of lipids in the *A. muciniphila* cell membrane, including canonical bacterial membrane components mainly PE, glycerophosphoglycerols (PG) and their lysophospholipid derivatives, highlighting the biochemical complexity of the *A. muciniphila* membrane lipidome (Examples in Supplementary Figure S7).

Identified OLs were isolated and structurally characterized by high-resolution LC-MS/MS (Supplementary Figure S8, Supplementary Tables S2 and S3, and Supplementary Note S2). MS/MS fragmentation patterns confirmed the presence of an ornithine head group, further validated by nuclear magnetic resonance (NMR) spectroscopy (Figure 4b, Supplementary Figure S9). Detailed tandem MS analysis showed that OLs contain different fatty acid (FA) compositions, with C14:0 predominantly found in OL 29:0, while OL 30:0, OL 31:0 and OL 32:0 contained a C15:0 FA chain (Supplementary Figure S10). Using an optimized LC-MS method (see Supplementary Note S2), we identified OL 15:0/14:0, OL 15:0/15:0, OL 16:0/15:0, and OL 17:0/15:0 as the most abundant OLs, with representative EICs of OL 30:0 and OL 31:0, indicating the presence of multiple structural isomers (Figure 4c).To align with established fatty amide lipid nomenclature and previous studies on OLs,^45^^,^^46^ we designate the amide-linked FA as the first moiety and the ester-bound FA as the second. Although OLs share structural similarities with fatty acid esters of hydroxy fatty acids (FAHFA), their annotation follows the opposite convention.^47^

To determine whether the FA and hydroxy fatty acid (OH-FA) moieties of OLs were straight-chain or branched-chain and define the position and stereochemistry of the hydroxy group, we performed sequential alkaline hydrolysis. The ester bond was first cleaved to release the ester-linked FA, followed by amide bond hydrolysis, which liberated free *L*-ornithine and the corresponding OH-FA. Comparative analysis of hydrolyzed OL fractions and FA standards, including straight-chain, iso-, and anteiso-branched species, identified ester-linked straight-chain and anteiso-branched C15:0 FAs in OL 16:0/15:0 (Supplementary Figure S11a). Anteiso-branched C15:0 FAs have previously been identified as the predominant species in phospholipids derived from *A. muciniphila* cell pellet.^22^ Amide-linked straight-chain OH-FAs were identified using authentic OH-FA standards, confirming the presence of both straight-chain 3-hydroxypentadecanoic acid (3-OH-C15:0) and 3-hydroxypalmitic acid (3-OH-C16:0) in OL 15:0/15:0 and OL 16:0/15:0, respectively (Supplementary Figure S11b, c, Supplementary Note S2).

We synthesized and characterized an enantiomerically enriched 3(*R*)-OH-C16:0 standard (Supplementary Note S3 and S4) to determine the absolute stereochemistry of *A. muciniphila*-derived 3-OH-FAs in OL 16:0/15:0. Its configuration was confirmed by NMR analysis following derivatization into *α*-O-acetyl mandelate diastereomers (Supplementary Figure S12, Supplementary Table S4). Subsequently, chiral chromatography was performed to compare the synthetic standard with the natural 3-OH-C16:0 obtained from hydrolyzed OL 16:0/15:0 sample, confirming that the straight-chain 3-OH-FA has an (*R*) configuration (Figure 4d). Altogether, structural analyzes confirm that isolated OL 16:0/15:0 exclusively contains an (*S*)-configured ornithine head group and a 3(*R*)-configured OH-FA (Figure 4d, Supplementary Figure S13).

**Figure 5.**
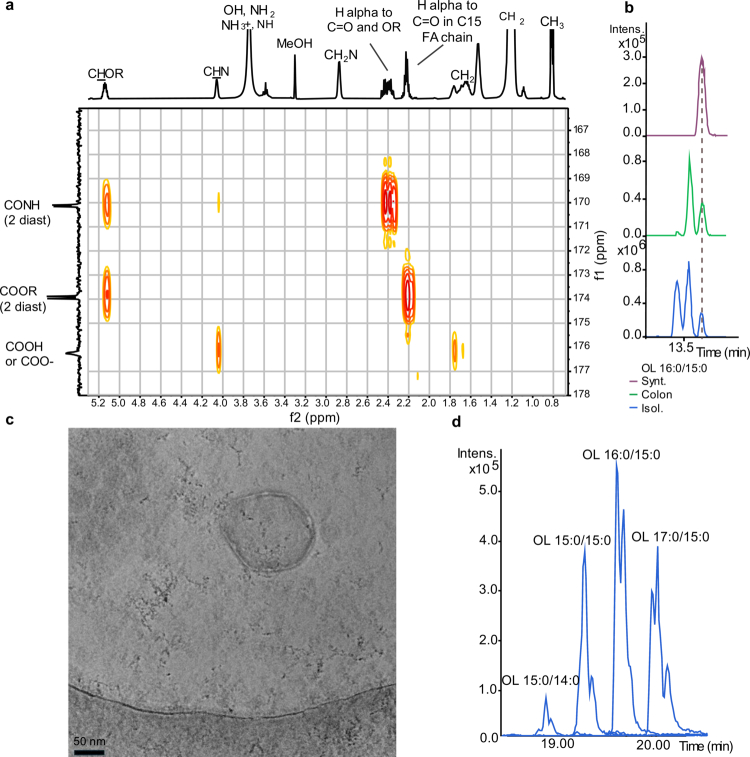
Characterization of synthetic OL 16:0/15:0 and OL-containing vesicles derived from *A. muciniphila*. (a) Structural elucidation of synthetic OL 16:0/15:0 using NMR spectroscopy. The HMBC spectrum (600 MHz, CDCl₃/MeOH-d₄ 9:1) confirms the amide linkage between the ornithine moiety and the FAHFA. The ester bond is positioned at C3 relative to the amide, while the *δ*-primary amine of ornithine remains unmodified. (b) EICs comparing synthetic OL 16:0/15:0 with natural OLs 16:0/15:0 detected in colon content and those isolated from the *A. muciniphila* cell pellet. (c) Representative cryo-electron microscopy (cryo-EM) micrograph of bacterial vesicles (~220 nm in diameter) isolated from *A. muciniphila* cultures (Scale bar: 50 nm, magnification: 105,000 × ). (d) EICs display key peaks of distinct OL species, highlighting the enrichment of OLs in vesicles derived from *A. muciniphila*.

Among all detected OL species, OL 30:0 and OL 31:0 were the most abundant and showed a significant increase in colonic samples at day 0. OL 30:0 has recently been described for its immunomodulatory effects and, along with other OL species, has been detected in the healthy human and murine gut.^48^ However, OL 31:0 has not been structurally or functionally characterized to date. To further investigate this lipid structure and function, we chemically synthesized OL 16:0/15:0 with straight-chain FAs as a diastereomeric mixture, followed by full structural characterization using ¹H and ¹³C NMR spectroscopy (Figure 5a, Supplementary Note S5). Comparative chromatographic analysis confirmed that the synthetic OL matched its natural counterpart, validating the assigned structure (Figure 5b). Additionally, both the (3*R*,*S*)-epimer, identified in *A. muciniphila*, and the (3*S*,*S*)-epimer were obtained by chiral separation (Supplementary Figure S14) of the protected diastereomeric OL 16:0/15:0 mixture, which consisted of a ~1:1 (3*R,S*:3*S,S*) ratio (Supplementary Table S5), followed by subsequent hydrogenation. All synthetic OL standards and intermediates were fully characterized by NMR and MS (Supplementary Note S6).

Due to the established role of bacterial OMVs as lipid delivery systems, we investigated whether OLs were associated with *A. muciniphila* OMVs. Cryo-electron microscopy (cryo-EM) data acquisition of OMVs extracted from *A. muciniphila* culture revealed vesicular structures ranging from 40 to 300 nm in diameter (Figure 5c), consistent with previous reports on *A. muciniphila* OMV.^49^ LC-MS analysis of vesicle extracts confirmed the presence of OLs, including OL 16:0/15:0, indicating that these lipids are integral components of *A. muciniphila* vesicles (Figure 5d). Additionally, other lipid species, previously identified in *A. muciniphila* total membrane, were also detected in OMVs extracts (Supplementary Figure S15).

### Immunomodulatory properties and metabolism of OL 16:0/15:0 in BMDMs

To evaluate the immunomodulatory properties of OL 16:0/15:0, we assessed its impact on IL-1β regulation in BMDMs. We investigated whether OL 16:0/15:0, tested as both a diastereomeric mixture (3*R*,*S* + 3*S*,*S*) and as its naturally occurring (3*R*,*S*)-epimer, differentially modulates these pathways.

**Figure 6.**
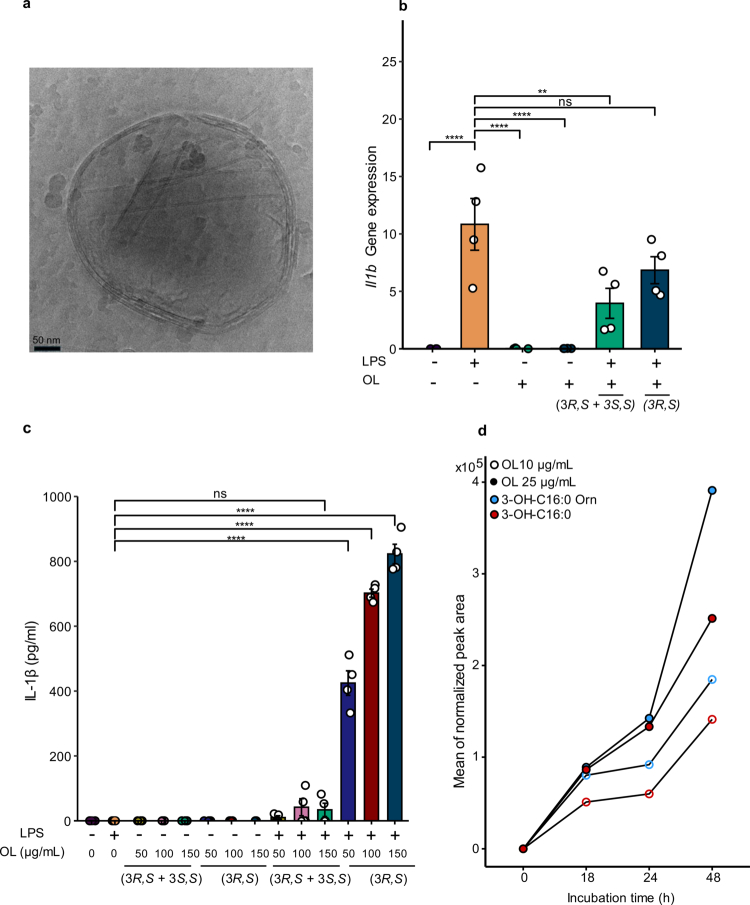
Functional analysis and metabolic fate of synthetic OL 16:0/15:0 in BMDMs. (a) Example of cryo-EM micrograph of synthetic OL 16:0/15:0 liposome (~430 nm in diameter), illustrating their nanoscale morphology (scale bar: 50 nm, magnification 105,000 × ). (b) *Il1b* mRNA expression in BMDMs stimulated with LPS (10 ng/mL) and OL 16:0/15:0 diastereomeric (3*R,S* + 3*S,S*) or epimer (3*R,S*) forms. Expression was normalized to *Hprt* and analyzed *via* qPCR. Data are presented as mean values of technical duplicates from two independent experiments. (c) ELISA quantification of IL-1β detected in supernatants, comparing the diastereomeric *(3R,S + 3S,S)* OL mixture and the *(3R,S)*-epimer effects at different concentrations. qPCR (b) and ELISA (c) were performed from the same experiments, using supernatants and cell pellets collected from identical BMDM cultures. Data in both plots represent the mean values ± SEM, with statistical significance determined using one-way ANOVA with Dunnett’s multiple comparisons test (****p ≤ 0.0001). (d) LC–MS analysis of OL 16:0/15:0 *(3R,S + 3S,S)* metabolism in BMDMs. Cells were treated with OL, and supernatants were collected at 0, 18, 24, and 48 h. Peak areas were normalized to medium spiked with OL alone. Data represent the mean values from independent biological replicates. Metabolites detected included 3-OH-C16:0-Orn, corresponding to the lyso-ornithine lipid species and 3-hydroxy-palmitic acid (3-OH-C16:0).

OLs were formulated as liposomes, mimicking their potential delivery through OMVs (Figure 6a). In LPS-stimulated BMDMs, treatment with the diastereomeric OL mixture (3*R*,*S* + 3*S*,*S*) markedly suppressed *Il1b* gene expression and significantly reduced both *Il6* transcription and IL-6 secretion (Figure 6b, Supplementary Figure S16a, b). However, the synthetic (3*R,S*)-epimer, found in *A. muciniphila,* selectively induced IL-1β secretion in LPS-primed BMDMs without affecting *Il1b* transcription (Figure 6b,c). Notably, OL 16:0/15:0, in either its diastereomeric mixture (3*R*,*S* + 3*S*,*S*) or (3*R*,*S*)-epimer form, did not trigger IL-1β secretion in unstimulated BMDMs (Figure 6c).

Together, these results support a stereoselective immunoregulatory effect, whereby the *(3R,S + 3S,S)* mixture dampens NF-κB–driven cytokine expression, while the (*3R,S*) epimer acts downstream of priming to promote IL-1β maturation in LPS-activated macrophages.

To characterize the time-dependent metabolic fate of OL 16:0/15:0, we performed LC-MS-based lipidomics on BMDM supernatants treated with two different concentrations of OL. Internalized OLs were partially metabolized, generating *N*^ɑ^-(3-hydroxypalmitoyl)-*L-*ornithine (3-OH-C16:0 Orn) and free 3-OH-C16:0 (Figure 6d). Our findings suggest that 3-OH-C16:0-Orn, a lyso-ornithine lipid (LOL) previously described in the literature,^50^ potentially produced through enzymatic cleavage of OL 16:0/15:0 by phospholipase A₂ (PLA₂), a mechanism previously reported for the hydrolysis of bioactive *N*-acyl lipids.^51^ Moreover, 3-OH-C16:0 progressively accumulated in macrophage supernatants, particularly at 48 hours post-treatment, suggesting amide hydrolysis of 3-OH-C16:0 Orn (Figure 6d, Supplementary Figure S17a). Notably, we observed a significant upregulation of *N*-acylethanolamine acid amidase (*NAAA*) gene expression in BMDMs at 48 hours after OL 16:0/15:0 treatment, which was associated with an accumulation of 3-OH-C16:0 (Supplementary Figure S17b). To evaluate the functional role of OL-derived metabolites, we treated BMDMs with synthetic 3-OH-C16:0 and measured the expression of inducible nitric oxide synthase (iNOS), a well-established inflammatory marker.^52^ Treatment with 3-OH-C16:0 significantly reduced *INOS* expression in LPS-stimulated BMDMs, suggesting a potential anti-inflammatory activity of this metabolite (Supplementary Figure S17c).

These findings suggest that OL 16:0/15:0 may modulate macrophage activation both directly and through its metabolic conversion into lipid-derived mediators with immunoregulatory potential.

## Discussion

Host–microbiota interactions, a key factor in IBD pathogenesis, are critically shaped by microbial metabolites that modulate immune responses and maintain intestinal homeostasis.^53^^,^^54^ While short-chain fatty acids, long-chain fatty acids,^26^ bile acids, and tryptophan metabolites have been extensively characterized as microbial modulators of host physiology,^55–57^ emerging evidence points to the bacterial lipid potential as modulators of host immunity.^28^ Among these, Chen et al.^26^ established the mechanistic link between microbial lipid metabolism and host immune regulation. They identified *A. muciniphila*–derived palmitoleic acid as a microbiota-dependent long-chain fatty acid that modulates TNF expression through type I interferon (IFN-I) signaling. Notably, palmitoleic acid levels were significantly reduced during disease progression, indicating that disease-associated inflammation alters the production of this immunoregulatory lipid. Their work further revealed that host *Ifnar1* variants modulate microbial palmitoleic acid synthesis, thereby shaping inflammatory responses and disease susceptibility.

Consistent with these findings, our study shows that bacterial lipids undergo dynamic remodeling during colitis and may represent an additional layer of immune regulation. Using a gnotobiotic *C. rodentium*-induced colitis model, we observed a marked shift in the intestinal lipidome, characterized by an increase in host-derived lipids and a concomitant depletion of microbial lipids at the peak of inflammation. Among the altered bacterial lipids, OLs produced by *A. muciniphila* were significantly reduced during inflammation, pointing to a potential role in shaping immune responses in this disease context.

*A. muciniphila*, a gut commensal with well-characterized immunoregulatory properties, has been extensively studied for its role in metabolic homeostasis,^58^ and has also been shown to enhance the efficacy of chemotherapy in colorectal cancer.^59^ Recent work demonstrated that *A. muciniphila*-derived lipids mediate anti-inflammatory effects by epigenetically repressing TNF expression.^26^ Our findings reveal a distinct class of *A. muciniphila* lipids, OLs, that modulate immune responses through structurally dependent and mechanistically diverse pathways. While bacterial OLs have been implicated in immune modulation *in vitro,*^60^ their functional roles in host-microbiome interactions and gut inflammation remain unclear.

The mechanisms underlying the observed depletion of OLs at peak inflammation remain to be elucidated. Several biological processes could contribute to these observations. One possibility is that OL biosynthesis by *A. muciniphila* is downregulated in response to inflammatory stress, leading to reduced lipid production.^61^ However, the genes responsible for OL biosynthesis in *A. muciniphila* have not yet been identified, representing a knowledge gap that limits our understanding of how the production of these lipids is regulated, particularly under inflammatory conditions. Consistent with this concept, Chen et al.^26^ reported that host IFN-I signaling modulated by *Ifnar1* genetic variants affects both *A. muciniphila* colonization and the microbial synthesis of palmitoleic acid during infection. Their findings provide direct evidence that cytokine-driven inflammatory pathways can modulate bacterial lipid metabolism and, in turn, influence the homeostasis of the gut microbial ecosystem.

Alternatively, OLs may be degraded by host- or microbiota-derived enzymes within the inflamed intestinal environment.^62^ In addition, OMVs containing OLs may be redistributed or more actively internalized by host immune cells, such as macrophages, during inflammation.^63–66^ Such redistribution could mechanistically link the observed decline in colonic OL levels to the induced IL-1β responses seen *in vitro*, as OLs delivered via OMVs may preferentially engage host immune pathways. Notably, OMVs derived from *A. muciniphila* have been reported to exert protective effects in colitis models by restoring intestinal homeostasis, potentially via their bioactive metabolites or lipid content.^67^ Intestinal inflammation also increases barrier permeability, facilitating the translocation of microbiota-derived OMVs to underlying tissues and immune cells.^68^ During inflammation, macrophages are actively recruited^69^ and exhibit enhanced phagocytic activity under inflammatory conditions,^70^ potentially promoting OMV uptake. Moreover, *A. muciniphila*-derived OMVs have been shown to cross the intestinal barrier and interact with both immune and epithelial cells.^67^^,^^71^

Previous studies identified OL species such as OL 16:0/16:0, OL 17:0/15:0, and OL 14:0/14:0, as bacterial-derived molecules with immunomodulatory properties.^72^^,^^73^ However, their dynamic regulation during intestinal inflammation and the specific role of OL 16:0/15:0 produced by *A. muciniphila* have not been previously investigated. To initiate such investigation, we isolated and structurally characterized OL 16:0/15:0, a lipid selectively modulated during inflammation. We confirmed its structure as a straight-chain C15:0 FA esterified to (3*R*)-hydroxypalmitic acid (C16:0), linked via an amide bond to the *α*-amino group of *L*-ornithine. Its presence in biological samples was confirmed by comparing retention time and fragmentation patterns with custom-synthesized OL and 3-OH-FA epimers, whose configurations were prior verified using 1D and 2D NMR analyzes.

To assess its immunological role, we evaluated the effect of OL 16:0/15:0 on IL-1β production in BMDMs. IL-1β is a key pro-inflammatory cytokine that contributes to pathogen clearance and intestinal immune homeostasis and acts as a significant mediator of inflammation in IBD. Our results revealed distinct immunological outcomes depending on OL stereochemistry that, hereafter, we attempted to link with previously reported data. The diastereomeric mixture (3*R*,*S* + 3*S*,*S*), formulated into liposomes, reduced *Il1b* and *IL-6* mRNA expression in LPS-stimulated macrophages, consistent with previous studies reporting that the diastereomeric mixture of OLs can modulate TLR4 signaling.^73^ This observation aligns with reduced NF-κB pathway activity.^74^^,^^75^

In contrast, the enantiopure (3*R*,*S*)-epimer, naturally produced by *A. muciniphila* and formulated identically, selectively induced the release of mature IL-1β in LPS-primed macrophages without significantly affecting *Il1b* transcription. Neither LPS nor OL alone was sufficient to induce IL-1β secretion. This suggests that the (3*R*,*S*) OL does not activate TLR4 signaling independently but instead acts downstream of priming, most likely by activating the inflammasome as a secondary signal. Such a mechanism aligns with the two-signal model of inflammasome activation, in which a priming stimulus (e.g., LPS) induces *Il1b* and *Nlrp3* expression, and a secondary signal (e.g., ATP, nigericin, ROS, Ca²⁺, lysosomal stress, or lipids) drives inflammasome assembly and IL-1β maturation.^76–79^ Moreover, direct recognition of pathogen- or damage-associated molecular patterns (PAMPs/DAMPs), can also promote potassium efflux through pannexin-1 channels, a well-established trigger of NLRP3 activation. Importantly, several lipid species, including phospholipids such as platelet-activating factor (PAF) and PAF-like lipids, have been shown to activate the canonical NLRP3 inflammasome through mechanisms involving both K⁺ efflux and Ca²⁺ influx, independently of the PAF receptor.^80^

These findings demonstrate that inflammasome activation can be triggered by diverse molecular signals and suggest that OL 16:0/15:0 may represent a previously unrecognized lipid-derived stimulus. Nevertheless, additional specific evidence is required to demonstrate that the observed response to OL reflects an authentic inflammasome activation.

We must also point out that the (3*S,S*)-epimer, present only in the synthetic diastereomeric mixture and not detected in the *A. muciniphila* cell membrane, may contribute to the transcriptional repression observed with the mixture. Although not tested individually, its presence could counteract or modulate the activity of the natural (*3R,S*)-epimer, thereby reducing *Il1b* mRNA levels without altering IL-1β secretion.

While bacterial OLs have been implicated in innate immune pathways,^73^ their stereochemical effects remain poorly defined. Most previous studies have mainly investigated OLs as stereoisomeric mixtures or crude lipid fractions,^48^^,^^73^^,^^81^ leaving the immunomodulatory properties of enantiomerically pure OLs uncharacterized. Kawai et al.^71^ demonstrated that diastereomeric mixtures of OL 16:0/16:0 and OL 17:0/15:0 activate CD14-dependent TLR4 signaling and induce TNF-*α* secretion in murine macrophages. Similarly, Pizzuto et al.^73^ reported that the OL 14:0/14:0 stereoisomeric mixture acts as both a priming and activating signal for the NLRP3 inflammasome, functioning as a partial TLR4 agonist in BMDMs. Interestingly, their findings showed that OL alone induced NF-κB activation but inhibited LPS-induced TLR4 signaling, revealing a dual role in immune modulation. Our results extend these findings by showing that enantiopure (3*R,S*)-OL 16:0/15:0, identified in *A. muciniphila*, stereoselectively induces IL-1β secretion in LPS-primed macrophages without directly engaging the NF-κB pathway.

These contrasting findings suggest that subtle variations in acyl chain length can impact immune responses. This may occur through modifications to receptor engagement, membrane interactions, or intracellular trafficking. However, other variables, including lipid purity (crude mixtures versus defined compounds), stereochemistry, and experimental context, may contribute to these divergent *in vitro* observations. Notably, OL 16:0/15:0 harbors a 3-hydroxy hexadecanoic acid moiety esterified with pentadecanoic acid. Such residues are hallmark components of lipid A in LPS and are recognized by innate immune sensors, including TLR4 and the non-canonical inflammasome receptor caspase-4/11.^82^^,^^83^ This structural motif is critical for receptor recognition and the immunostimulatory activity of bacterial lipids. Thus, the presence of a 3-hydroxy fatty acid in OL 16:0/15:0 may confer immunological properties distinct from OL species lacking this residue, potentially influencing its interaction with host receptors.

Bacterial lipids can reach host immune cells by several mechanisms, including delivery via OMVs and passive diffusion as free lipids.^41^ Once internalized by macrophages, these lipids are likely metabolized, resulting in the production and release of bioactive lipid-derived metabolites. Our data showed that OL 16:0/15:0 is internalized and metabolized by macrophages, generating ornithine-conjugated and free hydroxypalmitic acid (3-OH-C16:0-Orn and 3-OH-C16:0), most likely through an initial cleavage by phospholipase A₂ (PLA₂) followed by amide hydrolysis of 3-OH-C16:0-Orn catalyzed by *N*-acylethanolamine acid amidase. Enzymatic hydrolysis of amide bonds is known to involve *N*-acylethanolamine acid amidase, a lysosomal enzyme highly expressed in macrophages and B cells, which regulates fatty acid ethanolamide metabolism and modulates inflammation *via* peroxisome proliferator-activated receptor-*α* (PPAR-*α*).^84^

The accumulation of these metabolites suggests active lipid processing following uptake. Interestingly, 3-OH-C16:0 Orn is a microbiota-derived lipid previously identified for its immunomodulatory properties through sphingosine-1-phosphate receptor 4 (S1PR4), a key regulator of macrophage function and immune homeostasis.^50^ Treatment with synthetic 3-OH-C16:0 reduced *INOS* expression in LPS-stimulated macrophages, suggesting a potential regulatory effect of OL-derived metabolites.

## Conclusion and future directions

These findings, together with recent reports,^26^^,^^48^^,^^73^ indicate that *A. muciniphila* produces a range of structurally and functionally distinct lipid mediators that engage different immune pathways. Their abundance, stereochemistry, and structure determine distinct effects on macrophage activation, ranging from inhibition of NF-κB–dependent transcription to a potential activation of the inflammasome. Collectively, this emerging evidence underscores the complexity of *A. muciniphila*–derived lipid signaling as a context-dependent modulator of host immune responses.

Our data establish OL 16:0/15:0 as a microbiota-derived lipid dynamically regulated during colitis and as a potential modulator of inflammatory responses *in vitro*, in a stereochemistry-dependent manner. These results expand the current understanding of host-microbiota interactions, shifting the focus beyond small molecules to include microbial lipids as emerging immunomodulatory factors. Importantly, OLs produced by *A. muciniphila* have also been detected in human gut samples,^48^ supporting the potential relevance of these lipids in human intestinal health and disease. Moreover, given the broad distribution of OLs among gut-associated Gram-negative bacteria,^85^ future studies should explore whether OLs function as broad lipid-based signals in immune regulation.

While the *in vitro* findings in this study provide a mechanistic basis, the functional relevance of OLs *in vivo* within the context of intestinal inflammation remains to be clarified. Future work should assess OL 16:0/15:0 in murine colitis models to determine its impact on disease onset and progression. Extending cytokine profiling will also offer a broader view of its immunomodulatory spectrum. Since OLs are naturally present in *A. muciniphila*-derived OMVs, investigating OMV-mediated delivery could shed light on their bioavailability and tissue targeting *in vivo*. Finally, confirming inflammasome involvement will require targeted approaches, including the use of specific inhibitors.

Altogether, these findings establish the role of the OMM^12^-colonized gnotobiotic mouse model for dissecting microbiome-derived lipid signaling and identify OL 16:0/15:0 as a structurally defined bacterial lipid dynamically modulated during colitis, with potential implications for the pathogenesis of inflammatory bowel disease.

## Supplementary Material

Supplementary Material Supplementary figures.

## Data Availability

All LC-MS/MS data generated in this study are publicly available in the MassIVE repository under the following accession numbers: MSV000097399 (*C. rodentium* *in vivo* study), MSV000097546 (axenic mice samples), MSV000097402 (12 single bacterial strains from the *in vitro* analysis), MSV000097616 (Fermenter bacterial *in vitro* analysis), MSV000097422 (*A. muciniphila* OMV analysis) and MSV000097423 (Synthetic OL 16:0/15:0). These datasets can be accessed via https://massive.ucsd.edu NMR data obtained during this study are provided in supplementary information.

## References

[1] Deng W, Vallance BA, Li Y, Puente JL, Finlay BB. *Citrobacter rodentium* translocated intimin receptor (Tir) is an essential virulence factor needed for actin condensation, intestinal colonization and colonic hyperplasia in mice. Mol Microbiol. 2003;48(1):95–115. doi: 10.1046/j.1365-2958.2003.03429.x. 12657048

[2] Koroleva EP, Halperin S, Gubernatorova EO, Macho-Fernandez E, Spencer CM, Tumanov AV. *Citrobacter rodentium*-induced colitis: A robust model to study mucosal immune responses in the gut. J Immunol Methods. 2015;421:61–72. doi: 10.1016/j.jim.2015.02.003. 25702536 PMC12955730

[3] Khor B, Gardet A, Xavier RJ. Genetics and pathogenesis of inflammatory bowel disease. Nature. 2011;474(7351):307–317. doi: 10.1038/nature10209. 21677747 PMC3204665

[4] Lavelle A, Sokol H. Gut microbiota-derived metabolites as key actors in inflammatory bowel disease. Nat Rev Gastroenterol Hepatol. 2020;17(4):223–237. doi: 10.1038/s41575-019-0258-z. 32076145

[5] Su F, Su M, Wei W, Wu J, Chen L, Sun X, Liu M, Mao R, Bourgonje AR, Hu S. Integrating multi-omics data to reveal the host-microbiota interactome in inflammatory bowel disease. Gut Microbes. 2025;17(1). doi: 10.1080/19490976.2025.2476570. PMC11901428 40063366

[6] Brown EM, Temple ER, Jeanfavre S, Avila-Pacheco J, Taylor N, Liu K, Nguyen PN, Mohamed AM, Ung P, Walker RA, et al. *Bacteroides* sphingolipids promote anti-inflammatory responses through the mevalonate pathway. Cell Host Microbe. 2025;33(6):901–914.e6. doi: 10.1016/j.chom.2025.05.007. 40449488 PMC12169427

[7] Bao B, Wang Y, Boudreau P, Song X, Wu M, Chen X, Patik I, Tang Y, Ouahed J, Ringel A, et al. Bacterial sphingolipids exacerbate colitis by inhibiting ILC3-derived IL-22 Production. Cell Mol Gastroenterol Hepatol. 2024;18(2):101350. doi: 10.1016/j.jcmgh.2024.04.007. 38704148 PMC11222953

[8] Pan Y, Zhang H, Li M, He T, Guo S, Zhu L, Tan J, Wang B. Novel approaches in IBD therapy: targeting the gut microbiota-bile acid axis. Gut Microbes. 2024;16(1). doi: 10.1080/19490976.2024.2356284. PMC11110704 38769683

[9] Zhu Q, Korenfeld D, Suarez-Fueyo A, Graham S, Jin L, Punit S, Duffy R, Puri M, Caruso A, Hu C, et al. Epithelial dysfunction is prevented by IL-22 treatment in a *Citrobacter rodentium*-induced colitis model that shares similarities with inflammatory bowel disease. Mucosal Immunol. 2022;15(6):1338–1349. doi: 10.1038/s41385-022-00577-w. 36372810

[10] Luperchio SA, Schauer DB. Molecular pathogenesis of *Citrobacter rodentium* and transmissible murine colonic hyperplasia. Microbes Infect. 2001;3:333–340. doi: 10.1016/S1286-4579(01)01387-9 11334751

[11] Collins JW, Keeney KM, Crepin VF, Rathinam VAK, Fitzgerald KA, Finlay BB, Frankel G. *Citrobacter rodentium*: infection, inflammation and the microbiota. Nat Rev Microbiol. 2014;12(9):612–623. doi: 10.1038/nrmicro3315. 25088150

[12] Mullineaux-Sanders C, Sanchez-Garrido J, Hopkins EGD, Shenoy AR, Barry R, Frankel G. *Citrobacter rodentium*–host–microbiota interactions: immunity, bioenergetics and metabolism. Nat Rev Microbiol. 2019;17(11):701–715. doi: 10.1038/s41579-019-0252-z. 31541196

[13] Bhinder G, Sham HP, Chan JM, Morampudi V, Jacobson K, Vallance BA. The *Citrobacter rodentium* mouse model: studying pathogen and host contributions to infectious colitis. J Vis Exp. 2013;50222(72). doi: 10.3791/50222. PMC3605715 23462619

[14] Gentry EC, Collins SL, Panitchpakdi M, Belda-Ferre P, Stewart AK, Carrillo Terrazas M, Lu H, Zuffa S, Yan T, Avila-Pacheco J, et al. Reverse metabolomics for the discovery of chemical structures from humans. Nature. 2023;626:419–426. doi: 10.1038/s41586-023-06906-8. 38052229 PMC10849969

[15] Diab J, Hansen T, Goll R, Stenlund H, Ahnlund M, Jensen E, Moritz T, Florholmen J, Forsdahl G. Lipidomics in ulcerative colitis reveal alteration in mucosal lipid composition associated with the disease state. Inflamm Bowel Dis. 2019;25(11):1780–1787. doi: 10.1093/ibd/izz098. 31077307

[16] Brown EM, Ke X, Hitchcock D, Jeanfavre S, Avila-Pacheco J, Nakata T, Arthur TD, Fornelos N, Heim C, Franzosa EA, et al. *Bacteroides*-derived sphingolipids are critical for maintaining intestinal homeostasis and symbiosis. Cell Host Microbe. 2019;25(5):668–680.e7. doi: 10.1016/j.chom.2019.04.002. 31071294 PMC6544385

[17] Song X, Sun X, Oh SF, Wu M, Zhang Y, Zheng W, Geva-Zatorsky N, Jupp R, Mathis D, Benoist C, et al. Microbial bile acid metabolites modulate gut RORγ+ regulatory T cell homeostasis. Nature. 2020;577(7790):410–415. doi: 10.1038/s41586-019-1865-0. 31875848 PMC7274525

[18] Simopoulos AP. Omega-3 fatty acids in inflammation and autoimmune diseases. J Am Coll Nutr. 2002;21(6):495–505. doi: 10.1080/07315724.2002.10719248. 12480795

[19] Masoodi M, Pearl DS, Eiden M, Shute JK, Brown JF, Calder PC, Trebble TM. Altered colonic mucosal polyunsaturated fatty acid (PUFA) derived lipid mediators in ulcerative colitis: new insight into relationship with disease activity and pathophysiology. PLOS One. 2013;8:e76532. doi: 10.1371/journal.pone.0076532. 24204637 PMC3799829

[20] Nieto N, Fernandez MI, Gil A. Dietary monounsaturate d n-3 and n-6 long-chain polyunsaturate d fatty acids affect cellular antioxidant defense system in rats with experimental ulcerative colitis induced by trinitrobenzene sulfonic acid. Dig Dis Sci. 1998;43(12):2676–2687. doi: 10.1023/A:1026655311878. 9881500

[21] Bang S, Shin YH, Ma X, Park SM, Graham DB, Xavier RJ, Clardy J. A cardiolipin from *Muribaculum intestinale* induces antigen-specific cytokine responses. J Am Chem Soc. 2023;145(43):23422–23426. doi: 10.1021/jacs.3c09734. 37871232 PMC10623554

[22] Bae M, Cassilly CD, Liu X, Park SM, Tusi BK, Chen X, Kwon J, Filipčík P, Bolze AS, Vlamakis H, et al. *Akkermansia muciniphila* phospholipid induces homeostatic immune responses. Nature. 2022;608(7921):168–173. doi: 10.1038/s41586-022-04985-7. 35896748 PMC9328018

[23] Derrien M, Belzer C, De Vos WM. *Akkermansia muciniphila* and its role in regulating host functions. Microb Pathog. 2017;106:171–181. doi: 10.1016/j.micpath.2016.02.005. 26875998

[24] Garcia-Vello P, Tytgat HLP, Elzinga J, Van Hul M, Plovier H, Tiemblo-Martin M, Cani PD, Nicolardi S, Fragai M, De Castro C, et al. The lipooligosaccharide of the gut symbiont Akkermansia muciniphila exhibits a remarkable structure and TLR signaling capacity. Nat Commun. 2024;15(1):8411. doi: 10.1038/s41467-024-52683-x. 39333588 PMC11436972

[25] Wu Z, Xu Q, Gu S, Chen Y, Lv L, Zheng B, Wang Q, Xia J, Yang L, Bian X, et al. *Akkermansia muciniphila* ameliorates *Clostridioides difficil*e infection in mice by modulating the intestinal microbiome and metabolites. Front Microbiol. 2022;13. doi: 10.3389/fmicb.2022.841920. PMC9159907 35663882

[26] Chen L, Zhang G, Li G, Wang W, Ge Z, Yang Y, He X, Liu Z, Mai Q, Pi J, et al. Ifnar gene variants influence gut microbial production of palmitoleic acid and host immune responses to tuberculosis. Nat Metab. 2022;4(3):359–373. doi: 10.1038/s42255-022-00547-3. 35288721

[27] Bian X, Wu W, Yang L, Lv L, Wang Q, Li Y, Ye J, Fang D, Jiang X, Shi D. Administration of akkermansia muciniphila ameliorates dextran sulfate sodium-induced ulcerative colitis in mice. Front Microbiol. 2019;10:2259. doi: 10.3389/fmicb.2019.02259. 31632373 PMC6779789

[28] Chen Y, Mai Q, Chen Z, Lin T, Cai Y, Han J, Wang Y, Zhang M, Tan S, Wu Z, et al. Dietary palmitoleic acid reprograms gut microbiota and improves biological therapy against colitis. Gut Microbes. 2023;15(1). doi: 10.1080/19490976.2023.2211501. PMC10202094 37203220

[29] Eberl C, Ring D, Münch PC, Beutler M, Basic M, Slack EC, Schwarzer M, Srutkova D, Lange A, Frick JS, et al. Reproducible colonization of germ-free mice with the oligo-mouse-microbiota in different animal facilities. Front Microbiol. 2020;10:2999. doi: 10.3389/fmicb.2019.02999. 31998276 PMC6965490

[30] Weiss AS, Burrichter AG, Durai Raj AC, von Strempel A, Meng C, Kleigrewe K, Münch PC, Rössler L, Huber C, Eisenreich W, et al. In vitro interaction network of a synthetic gut bacterial community. ISME J. 2022;16(4):1095–1109. doi: 10.1038/s41396-021-01153-z. 34857933 PMC8941000

[31] Elhenawy W, Debelyy MO, Feldman MF. Preferential packing of acidic glycosidases and proteases into Bacteroides outer membrane vesicles. In: Whiteley M, Greenberg EP. mBio; 2014; Vol. 5, e00909-14. doi: 10.1128/mBio.00909-14. PMC3952158 24618254

[32] Sillner N, Walker A, Koch W, Witting M, Schmitt-Kopplin P. Metformin impacts cecal bile acid profiles in mice. J Chromatogr B. 2018;1083:35–43. doi: 10.1016/j.jchromb.2018.02.029. 29522956

[33] Dührkop K, Fleischauer M, Ludwig M, Aksenov AA, Melnik AV, Meusel M, Dorrestein PC, Rousu J, Böcker S. SIRIUS 4: a rapid tool for turning tandem mass spectra into metabolite structure information. Nat Methods. 2019;16(4):299–302. doi: 10.1038/s41592-019-0344-8. 30886413

[34] Dührkop K, Nothias LF, Fleischauer M, Reher R, Ludwig M, Hoffmann MA, Petras D, Gerwick WH, Rousu J, Dorrestein PC, et al. Systematic classification of unknown metabolites using high-resolution fragmentation mass spectra. Nat Biotechnol. 2021;39(4):462–471. doi: 10.1038/s41587-020-0740-8. 33230292

[35] Dührkop K, Shen H, Meusel M, Rousu J, Böcker S. Searching molecular structure databases with tandem mass spectra using CSI:fingerID. Proc Natl Acad Sci. 2015;112(41):12580–12585. doi: 10.1073/pnas.1509788112. 26392543 PMC4611636

[36] Li T, He J, Horvath G, Próchnicki T, Latz E, Takeoka S. Lysine-containing cationic liposomes activate the NLRP3 inflammasome: effect of a spacer between the head group and the hydrophobic moieties of the lipids. Nanomedicine Nanotechnol Biol Med. 2018;14(2):279–288. doi: 10.1016/j.nano.2017.10.011. 29127038

[37] Chassaing B, Srinivasan G, Delgado MA, Young AN, Gewirtz AT, Vijay-Kumar M. Fecal lipocalin 2, a sensitive and broadly dynamic non-invasive biomarker for intestinal inflammation. PLOS One. 2012;7(9):e44328. doi: 10.1371/journal.pone.0044328 . 22957064 PMC3434182

[38] Older EA, Zhang J, Ferris ZE, Xue D, Zhong Z, Mitchell MK, Madden M, Wang Y, Chen H, Nagarkatti P, et al. Biosynthetic enzyme analysis identifies a protective role for TLR4-acting gut microbial sulfonolipids in inflammatory bowel disease. Nat Commun. 2024;15(1):9371. doi: 10.1038/s41467-024-53670-y. 39477928 PMC11525784

[39] Wlodarska M, Willing B, Keeney KM, Menendez A, Bergstrom KS, Gill N, Russell SL, Vallance BA, Finlay BB, Bäumler AJ. Antibiotic treatment alters the colonic mucus layer and predisposes the host to exacerbated *Citrobacter rodentium*-induced colitis. Bäumler AJ, ed. Infect Immun 2011;79(4):1536–1545. doi: 10.1128/IAI.01104-10. PMC3067531 21321077

[40] Sartor RB. Microbial influences in inflammatory bowel diseases. Gastroenterology. 2008;134(2):577–594. doi: 10.1053/j.gastro.2007.11.059. 18242222

[41] Heaver SL, Johnson EL, Ley RE. Sphingolipids in host–microbial interactions. Curr Opin Microbiol. 2018;43:92–99. doi: 10.1016/j.mib.2017.12.011. 29328957

[42] Liebisch G, Plagge J, Höring M, Seeliger C, Ecker J. The effect of gut microbiota on the intestinal lipidome of mice. Int J Med Microbiol. 2021;311(3):151488. doi: 10.1016/j.ijmm.2021.151488. 33662870

[43] Morgan PK, Pernes G, Huynh K, Giles C, Paul S, Smith AAT, Mellett NA, Liang A, van Buuren-Milne T, Veiga CB, et al. A lipid atlas of human and mouse immune cells provides insights into ferroptosis susceptibility. Nat Cell Biol. 2024;26(4):645–659. doi: 10.1038/s41556-024-01377-z. 38589531

[44] Ivanova PT, Milne SB, Brown HA. Identification of atypical ether-linked glycerophospholipid species in macrophages by mass spectrometry. J Lipid Res. 2010;51(6):1581–1590. doi: 10.1194/jlr.D003715. 19965583 PMC3035522

[45] Liebisch G, Fahy E, Aoki J, Dennis EA, Durand T, Ejsing CS, Fedorova M, Feussner I, Griffiths WJ, Köfeler H, et al. Update on LIPID MAPS classification, nomenclature, and shorthand notation for MS-derived lipid structures. J Lipid Res. 2020;61(12):1539–1555. doi: 10.1194/jlr.S120001025. 33037133 PMC7707175

[46] Zhang X, Ferguson-Miller SM, Reid GE. Characterization of ornithine and glutamine lipids extracted from cell membranes of *Rhodobacter sphaeroides*. J Am Soc Mass Spectrom. 2009;20(2):198–212. doi: 10.1016/j.jasms.2008.08.017. 18835523 PMC2779474

[47] Brejchova K, Balas L, Paluchova V, Brezinova M, Durand T, Kuda O. Understanding FAHFAs: from structure to metabolic regulation. Prog Lipid Res. 2020;79:101053. doi: 10.1016/j.plipres.2020.101053. 32735891

[48] Zhang Q, Linke V, Overmyer KA, Traeger LL, Kasahara K, Miller IJ, Manson DE, Polaske TJ, Kerby RL, Kemis JH, et al. Genetic mapping of microbial and host traits reveals production of immunomodulatory lipids by A*kkermansia muciniphila* in the murine gut. Nat Microbiol. 2023;8(3):424–440. doi: 10.1038/s41564-023-01326-w. 36759753 PMC9981464

[49] Mofrad LZ, Fateh A, Sotoodehnejadnematalahi F, Asbi DNS, Davar Siadat S. The effect of *Akkermansia muciniphila* and its outer membrane vesicles on micrornas expression of inflammatory and anti-inflammatory pathways in human dendritic cells. Probiotics Antimicrob Proteins. 2024;16(2):367–382. doi: 10.1007/s12602-023-10058-6. 36884184

[50] Cohen LJ, Esterhazy D, Kim SH, Lemetre C, Aguilar RR, Gordon EA, Pickard AJ, Cross JR, Emiliano AB, Han SM, et al. Commensal bacteria make GPCR ligands that mimic human signalling molecules. Nature. 2017;549(7670):48–53. doi: 10.1038/nature23874. 28854168 PMC5777231

[51] Nemati R, Dietz C, Anstadt EJ, Cervantes J, Liu Y, Dewhirst FE, Clark RB, Finegold S, Gallagher JJ, Smith MB, et al. Deposition and hydrolysis of serine dipeptide lipids of Bacteroidetes bacteria in human arteries: relationship to atherosclerosis. J Lipid Res. 2017;58(10):1999–2007. doi: 10.1194/jlr.M077792. 28814639 PMC5625123

[52] Zamora R, Vodovotz Y, Billiar TR. Inducible nitric oxide synthase and inflammatory diseases. Mol Med. 2000;6(5):347–373. doi: 10.1007/BF03401781. 10952018 PMC1949959

[53] Thursby E, Juge N. Introduction to the human gut microbiota. Biochem J. 2017;474(11):1823–1836. doi: 10.1042/BCJ20160510. 28512250 PMC5433529

[54] Franzosa EA, Sirota-Madi A, Avila-Pacheco J, Fornelos N, Haiser HJ, Reinker S, Vatanen T, Hall AB, Mallick H, McIver LJ, et al. Gut microbiome structure and metabolic activity in inflammatory bowel disease. Nat Microbiol. 2018;4(2):293–305. doi: 10.1038/s41564-018-0306-4. 30531976 PMC6342642

[55] Parada Venegas D, De La Fuente MK, Landskron G, González MJ, Quera R, Dijkstra G, Harmsen HJM, Faber KN, Hermoso MA. Short chain fatty acids (SCFAs)-mediated gut epithelial and immune regulation and its relevance for inflammatory bowel diseases. Front Immunol. 2019;10:277. doi: 10.3389/fimmu.2019.00277. 30915065 PMC6421268

[56] Thomas JP, Modos D, Rushbrook SM, Powell N, Korcsmaros T. The emerging role of bile acids in the pathogenesis of inflammatory bowel disease. Front Immunol. 2022;13. doi: 10.3389/fimmu.2022.829525. PMC8850271 35185922

[57] Roager HM, Licht TR. Microbial tryptophan catabolites in health and disease. Nat Commun. 2018;9(1):3294. doi: 10.1038/s41467-018-05470-4. 30120222 PMC6098093

[58] Cani PD, Depommier C, Derrien M, Everard A, De Vos WM. *Akkermansia muciniphila*: paradigm for next-generation beneficial microorganisms. Nat Rev Gastroenterol Hepatol. 2022;19(10):625–637. doi: 10.1038/s41575-022-00631-9. 35641786

[59] Hou X, Zhang P, Du H, Chu W, Sun R, Qin S, Tian Y, Xu F. *Akkermansia muciniphila* potentiates the antitumor efficacy of FOLFOX in colon cancer. Front Pharmacol. 2021;12:725583. doi: 10.3389/fphar.2021.725583. 34603035 PMC8484791

[60] Kawai Y, Kaneda K, Morisawa Y, Akagawa K. Protection of mice from lethal endotoxemia by use of an ornithine-containing lipid or a serine-containing lipid. Infect Immun. 1991;59(8):2560–2566. doi: 10.1128/iai.59.8.2560-2566.1991. 1906840 PMC258056

[61] Morgan XC, Tickle TL, Sokol H, Gevers D, Devaney KL, Ward DV, Reyes JA, Shah SA, LeLeiko N, Snapper SB, et al. Dysfunction of the intestinal microbiome in inflammatory bowel disease and treatment. Genome Biol. 2012;13(9):R79. doi: 10.1186/gb-2012-13-9-r79. 23013615 PMC3506950

[62] Schoeler M, Caesar R. Dietary lipids, gut microbiota and lipid metabolism. Rev Endocr Metab Disord. 2019;20(4):461–472. doi: 10.1007/s11154-019-09512-0. 31707624 PMC6938793

[63] Fábrega MJ, Rodríguez-Nogales A, Garrido-Mesa J, Algieri F, Badía J, Giménez R, Gálvez J, Baldomà L. Intestinal anti-inflammatory effects of outer membrane vesicles from *Escherichia coli* Nissle 1917 in DSS-experimental colitis in mice. Front Microbiol. 2017;8:1274. doi: 10.3389/fmicb.2017.01274. 28744268 PMC5504144

[64] Engevik MA, Danhof HA, Ruan W, Engevik AC, Chang-Graham AL, Engevik KA, Shi Z, Zhao Y, Brand CK, Krystofiak ES, et al. *Fusobacterium nucleatum* secretes outer membrane vesicles and promotes intestinal inflammation. mBio. 2021;12(2):e02706-20. doi: 10.1128/mBio.02706-20. 33653893 PMC8092269

[65] Liu L, Liang L, Yang C, Zhou Y, Chen Y. Extracellular vesicles of Fusobacterium nucleatum compromise intestinal barrier through targeting RIPK1-mediated cell death pathway. Gut Microbes. 2021;13(1):1902718. doi: 10.1080/19490976.2021.1902718. 33769187 PMC8007154

[66] Hao H, Zhang X, Tong L, Liu Q, Liang X, Bu Y, Gong P, Xia Y, Ai L, Yi H. Effect of extracellular vesicles derived from lactobacillus plantarum Q7 on Gut microbiota and ulcerative colitis in mice. Front Immunol. 2021;12:777147. doi: 10.3389/fimmu.2021.777147. 34925349 PMC8674835

[67] Kang C sung, Ban M, Choi EJ, Moon HG, Jeon JS, Kim DK. Extracellular vesicles derived from gut microbiota, especially *Akkermansia muciniphila*, protect the progression of dextran sulfate sodium-induced colitis. PLOS One. 2013;8 e76520. doi: 10.1371/journal.pone.0076520. 24204633 PMC3811976

[68] Bittel M, Reichert P, Sarfati I, Dressel A, Leikam S, Uderhardt S, Stolzer I, Phu TA, Ng M, Vu NK, et al. Visualizing transfer of microbial biomolecules by outer membrane vesicles in microbe‐host‐communication in vivo. J Extracell Vesicles. 2021;10(12):e12159. doi: 10.1002/jev2.12159. 34664784 PMC8524437

[69] Lee CH, Choi EY. Macrophages and inflammation. J Rheum Dis. 2018;25(1):11. doi: 10.4078/jrd.2018.25.1.11.

[70] Chen S, Saeed AFUH, Liu Q, Jiang Q, Xu H, Xiao GG, Rao L, Duo Y. Macrophages in immunoregulation and therapeutics. Signal Transduct Target Ther. 2023;8(1):207. doi: 10.1038/s41392-023-01452-1. 37211559 PMC10200802

[71] Zheng T, Hao H, Liu Q, Li J, Yao Y, Liu Y, Zhang T, Yi H. Effect of extracelluar vesicles derived from *Akkermansia muciniphila* on intestinal barrier in colitis mice. Nutrients. 2023;15(22):4722. doi: 10.3390/nu15224722. 38004116 PMC10674789

[72] Kawai Y, Takasuka N, Inoue K, Akagawa K, Nishijima M. Ornithine-containing lipids stimulate CD14-dependent TNF-α production from murine macrophage-like J774.1 and RAW 264.7 cells. FEMS Immunol Med Microbiol. 2000;28(3):197–203. doi: 10.1111/j.1574-695X.2000.tb01477.x. 10865171

[73] Pizzuto M, Hurtado-Navarro L, Molina-Lopez C, Soubhye J, Gelbcke M, Rodriguez-Lopez S, Ruysschaert J, Schroder K, Pelegrin P. Ornithine lipid is a partial TLR4 agonist and NLRP3 activator. Cell Rep. 2024;43(10):114788. doi: 10.1016/j.celrep.2024.114788. 39340778

[74] Mitchell S, Vargas J, Hoffmann A. Signaling via the NFκB system. WIREs Syst Biol Med. 2016;8(3):227–241. doi: 10.1002/wsbm.1331. PMC8363188 26990581

[75] Oeckinghaus A, Ghosh S. The NF-κB family of transcription factors and its regulation. Cold Spring Harb Perspect Biol. 2009;1(4):a000034–a000034. doi: 10.1101/cshperspect.a000034. 20066092 PMC2773619

[76] Liu T, Zhang L, Joo D, Sun SC. NF-κB signaling in inflammation. Signal Transduct Target Ther. 2017;2(1):17023. doi: 10.1038/sigtrans.2017.23. 29158945 PMC5661633

[77] Cullen SP, Kearney CJ, Clancy DM, Martin SJ. Diverse activators of the NLRP3 inflammasome promote IL-1β secretion by triggering necrosis. Cell Rep. 2015;11(10):1535–1548. doi: 10.1016/j.celrep.2015.05.003. 26027935

[78] Kelley N, Jeltema D, Duan Y, He Y. The NLRP3 inflammasome: an overview of mechanisms of activation and regulation. Int J Mol Sci. 2019;20(13):3328. doi: 10.3390/ijms20133328. 31284572 PMC6651423

[79] Pellegrini C, Antonioli L, Lopez-Castejon G, Blandizzi C, Fornai M. Canonical and non-canonical activation of NLRP3 inflammasome at the crossroad between immune tolerance and intestinal inflammation. Front Immunol. 2017;8. doi: 10.3389/fimmu.2017.00036. PMC5263152 28179906

[80] Deng M, Guo H, Tam JW, Johnson BM, Brickey WJ, New JS, Lenox A, Shi H, Golenbock DT, Koller BH, et al. Platelet-activating factor (PAF) mediates NLRP3-NEK7 inflammasome induction independently of PAFR. J Exp Med. 2019;216(12):2838–2853. doi: 10.1084/jem.20190111. 31558613 PMC6888982

[81] Kawai Y, Akagawa K. Macrophage activation by an ornithine-containing lipid or a serine-containing lipid. Infect Immun. 1989;57(7):2086–2091. doi: 10.1128/iai.57.7.2086-2091.1989. 2499544 PMC313845

[82] Binding N, Jaschinski S, Werlich S, Bletz S, Witting U. Quantification of bacterial lipopolysaccharides (endotoxin) by GC–MS determination of 3-hydroxy fatty acids. J Env Monit. 2004;6(1):65–70. doi: 10.1039/B309237B. 14737472

[83] Raetz CRH, Reynolds CM, Trent MS, Bishop RE. Lipid a modification systems in gram-negative bacteria. Annu Rev Biochem. 2007;76(1):295–329. doi: 10.1146/annurev.biochem.76.010307.145803. 17362200 PMC2569861

[84] Ueda N, Tsuboi K, Uyama T. N-acylethanolamine metabolism with special reference to N-acylethanolamine-hydrolyzing acid amidase (NAAA). Prog Lipid Res. 2010;49(4):299–315. doi: 10.1016/j.plipres.2010.02.003. 20152858

[85] Vences-Guzmán MÁ, Geiger O, Sohlenkamp C. Ornithine lipids and their structural modifications: from A to E and beyond. FEMS Microbiol Lett. 2012;335(1):1–10. doi: 10.1111/j.1574-6968.2012.02623.x. 22724388

