# Supplementary material for: Ornithine lipids from Akkermansia muciniphila are dynamically modulated in colitis and shape macrophage inflammatory responses
Source: Gut Microbes. 2025 Dec 16;17(1):2601376. doi: 10.1080/19490976.2025.2601376 (PMC12710885; doi:10.1080/19490976.2025.2601376)
Supplement: Supplementary Material — Supplementary figures. [file KGMI_A_2601376_SM9735.docx]

# Supplementary Information

**Ornithine lipids from *Akkermansia muciniphila* are dynamically modulated in colitis and shape macrophage inflammatory responses**

Habiba Selmi^1^, Alesia Walker^1*^, Laurence Balas^2^, Marianna Lucio^1^, Markus Klotz^3^, Aicha Jeridi^3^, Anna G. Burrichter^4,5^, Devon Conti^6,7^, Lorenzo Chaffringeon^6,7^, Brice Beinsteiner^8^, Marion Jasnin^8^, Nicolas Vanthuyne^9^, Thierry Durand^2^, Ali Önder Yildirim^3,10^, Bärbel Stecher^4,5,11^, Laurent Debarbieux^6^, Philippe Schmitt-Kopplin^1,12^

^1^Research Unit Analytical BioGeoChemistry, Helmholtz Zentrum München, 85764 Neuherberg, Germany

^2^Institut des Biomolécules Max Mousseron, IBMM, Université de Montpellier, CNRS, ENSCM, 34093 Montpellier, France

^3^Institute of Lung Health and Immunity (LHI), Comprehensive Pneumology Center (CPC), Helmholtz Munich, Member of the German Center for Lung Research (DZL), 85764 Munich, Germany.

^4^Max von Pettenkofer Institute of Hygiene and Medical Microbiology, Faculty of Medicine, LMU Munich, Germany

^5^Chair of Intestinal Microbiome, Technical University of Munich, Freising, Germany

^6^Institut Pasteur, Université Paris Cité,CNRS UMR6047, Bacteriophage Bacterium Host, Paris, France

^7^Sorbonne Université, Collège Doctoral, Paris, France

^8^Helmholtz Pioneer Campus, Helmholtz Zentrum München, 85764 Neuherberg, Germany

^9^Aix Marseille Univ., CNRS, Centrale Med, FSCM, Chiropole, 13397 Marseille Cedex 20, France

^10^Institute of Experimental Pneumology, LMU University Hospital, Ludwig-Maximilian’s University, Munich, Germany

^11^German Center for Infection Research (DZIF), partner site LMU Munich, Germany

^12^Chair of Analytical Food Chemistry, Technical University of Munich, Freising, Germany

E-mail: alesia.walker@helmholtz-munich.de

HelmholtzZentrum München

Deutsches Forschungszentrum für Gesundheit und Umwelt

Department of Environmental Sciences

Analytical BioGeoChemistry

Ingolstaedter Landstrasse 1

85764 Neuherberg

Germany

##

**Table of Contents**

[Supplementary Information 0](#_Toc213237549)

[Supplementary Figures 2](#_Toc213237550)

[Supplementary Notes 13](#_Toc213237551)

[Supplementary Note S1. Bacterial strains and growth conditions 13](#_Toc213237552)

[Supplementary Note S2. Fractionation, FA identification and estimation of *A. muciniphila* OL concentration 14](#_Toc213237553)

[Supplementary Note S3. Synthesis of enantiomerically enriched 3(*R*)-OH-C16:0 (compound 10) 16](#_Toc213237554)

[Mandelate esters with (*R*)-(−)-α-Acetoxyphenylacetic acid (compound 11) 17](#_Toc213237555)

[Mandelate esters with (*S*)-(−)-α-Acetoxyphenylacetic acid (compound 12) 18](#_Toc213237556)

[Measurement of the enantiomeric excess (ee %) of alcohol 10 *via* ^1^H NMR of diastereomers 11 or 12 18](#_Toc213237557)

[Assignment of the absolute configuration in compound 9 *via* compounds 11 and 12. 18](#_Toc213237558)

[Supplementary Note S4. Enantiomerically enriched 3-HPA characterization (compound 10) 19](#_Toc213237559)

[Supplementary Note S5. Synthesis of OLs 21](#_Toc213237560)

[General strategy 21](#_Toc213237561)

[Materials and general methods for OL synthesis 21](#_Toc213237562)

[Synthesis of a diastereomeric mixture of OL 16/0/15:0 (compound 8) 22](#_Toc213237563)

[Synthesis of enantiopure OLs 16:0/15:0 (Compounds 8, diast. (3*S,S*)-8 and (3*R,S*)-8) 26](#_Toc213237564)

[Supplementary Note S6. Precursors, synthetic diastereomeric mixture and enantiopure OLs characterization (compound 2 to 12) 26](#_Toc213237565)

[Supplementary NMR spectra 32](#_Toc213237566)

[Supplementary Tables 52](#_Toc213237567)

[Table S1. Non-targeted lipidomics MS and MS/MS parameters (Sciex X500 QTOF) 52](#_Toc213237568)

[Table S2. Targeted lipidomics MS and MS/MS parameters (Bruker maXis QTOF). 53](#_Toc213237569)

[Table S3. Estimation of OL 16:0/15:0 concentration in the *A. muciniphila* cell pellet and colon content samples using authentic standard (compound 8). 54](#_Toc213237570)

[Table S4. Comparison of the chemical shifts (1H NMR in CDCl3, 298 K) in both compounds 11 and 12. 54](#_Toc213237571)

[Table S5. Chiral chromatographic separation parameters of both enantiopure epimers 7. 54](#_Toc213237572)

[References 55](#_Toc213237573)

## Supplementary Figures

**
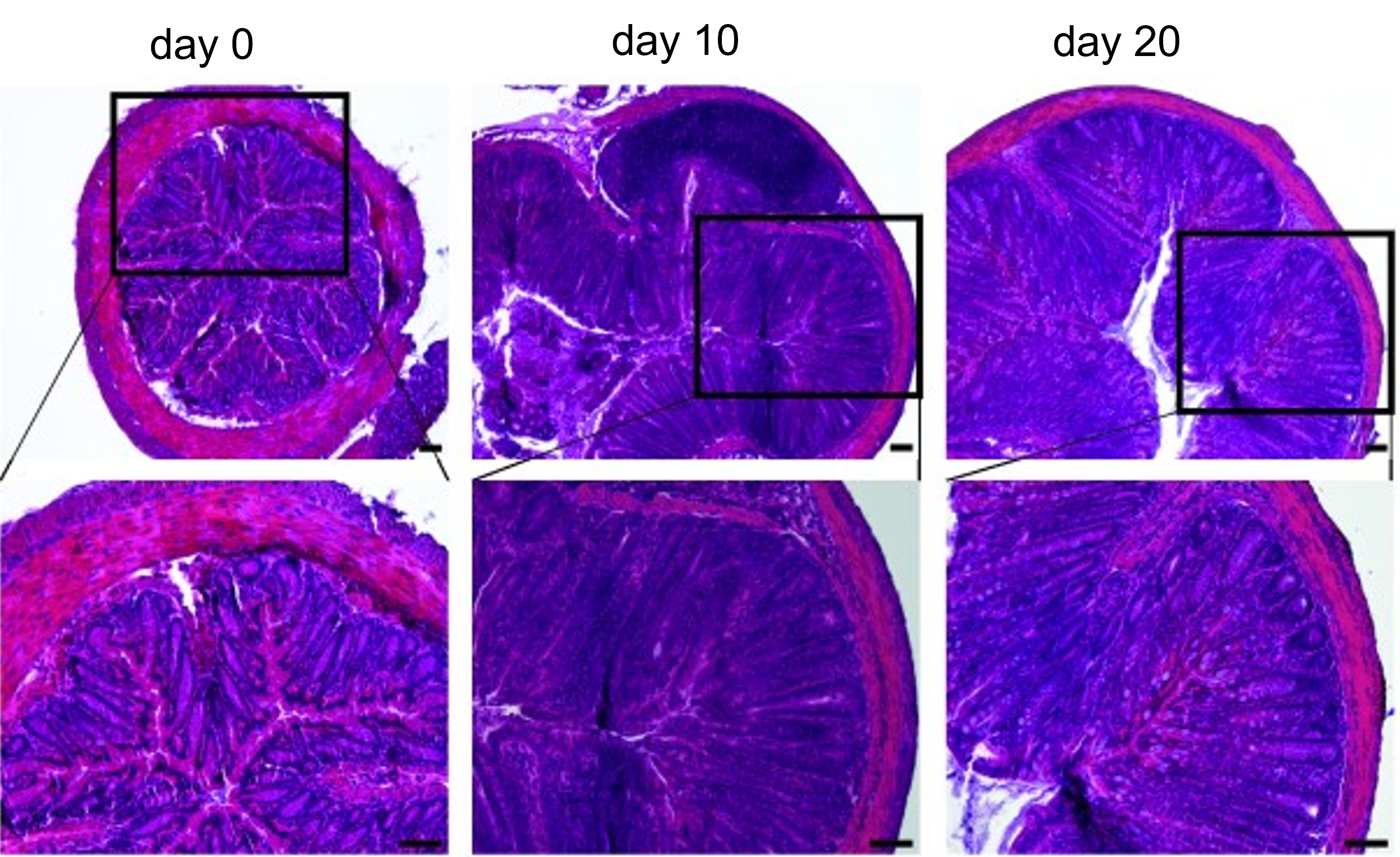
**

**Figure S1.** Histopathological characterization of the inflammatory response in *C. rodentium*-induced colitis model in OMM^12^ mice. Representative images of H&E staining of colon sections from OMM^12^ mice collected at day 0 (before infection), day 10 and day 20 post-infection by *C. rodentium*, show epithelial damage and inflammatory cell infiltration at day 10 compared to day 0, which were both reduced at day 20. Images are presented at two magnifications (top, ×10; bottom, ×20). Scale bars, 50 µm.

**
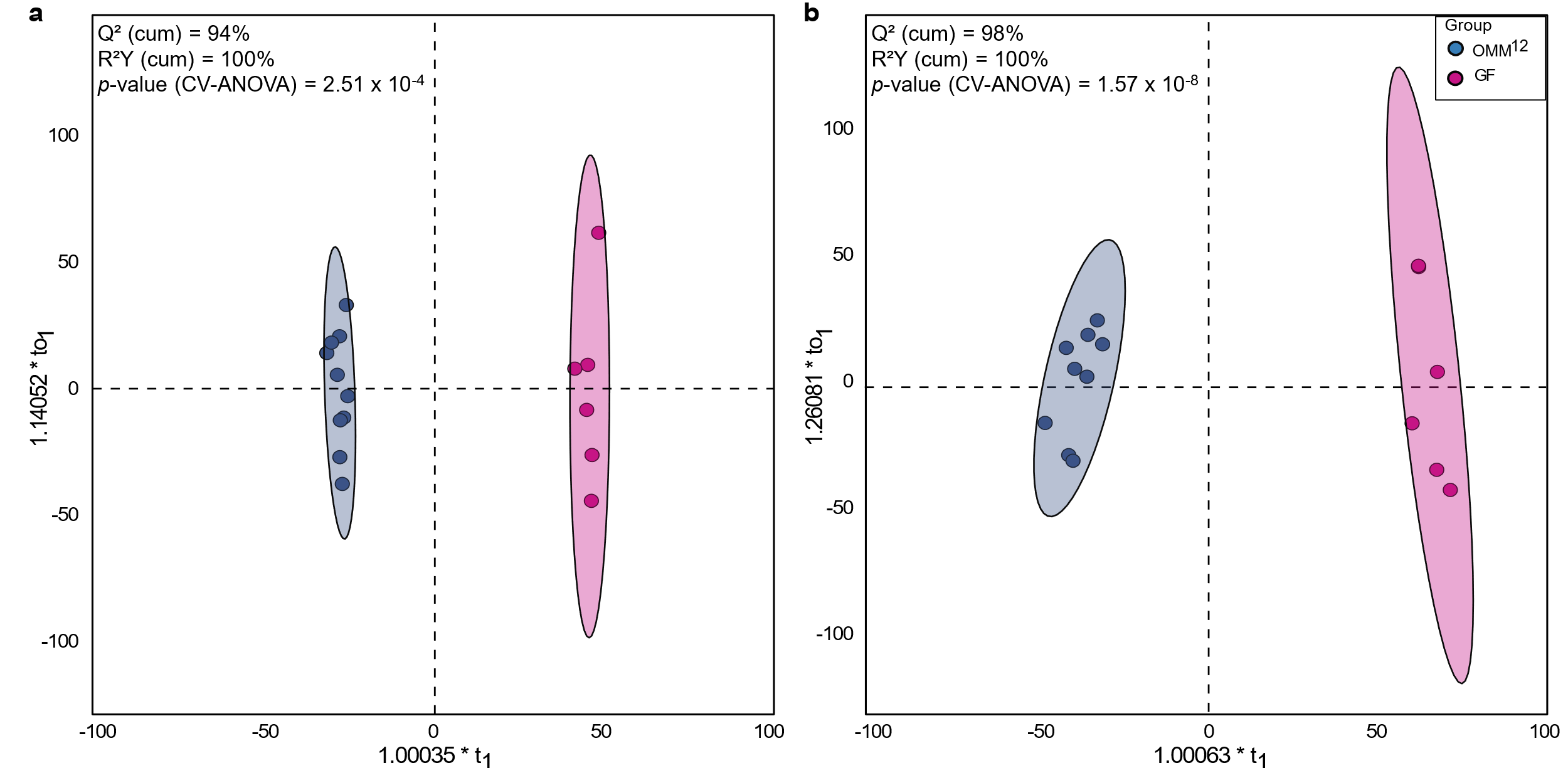
Figure S2.** Score plot of OMM^12^ and GF groups showing clear separation by metabolite profiles. (a) OPLS-DA score plot of ileal and (b), colonic metabolite and lipid profiles from OMM^12^ and GF mice. The ellipses represent 95% confidence intervals for each group, highlighting the clear separation between OMM^12^ (blue ellipse) and GF (purple ellipse) groups.


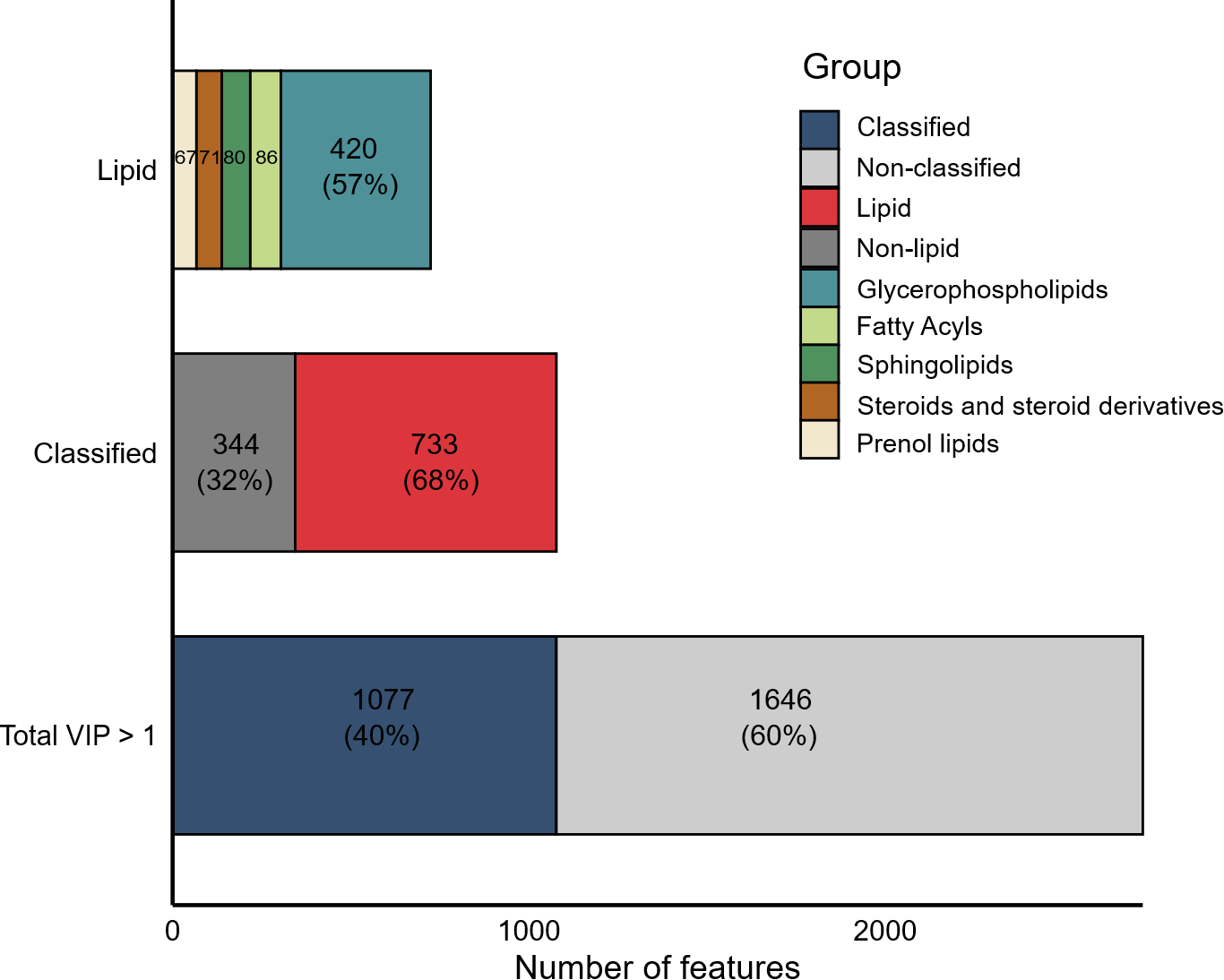


**Figure S3. Distribution of significant features (VIP > 1) and lipid classification.** Horizontal bars show the total number of features with VIP > 1 (n = 2723). Of these, 40% were classified (n = 1077). Within the classified set, 68% (~27% of all features) were identified as lipids. Major lipid classes are shown separately, with glycerophospholipids representing the most abundant group.


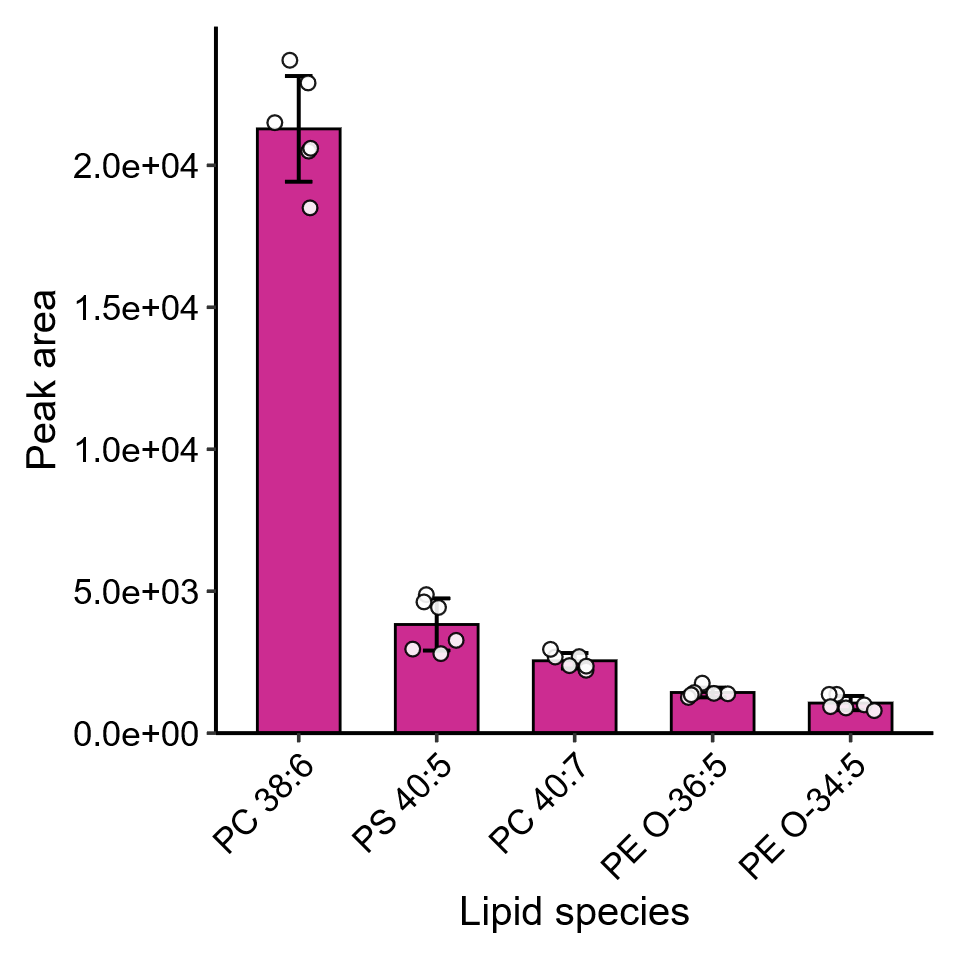


**Figure S4.** Examples of lipid species detected in colonic tissue samples of GF mice. Bar plots represent the mean peak area ± standard deviation for each lipid, with individual measurements shown as overlaid points.


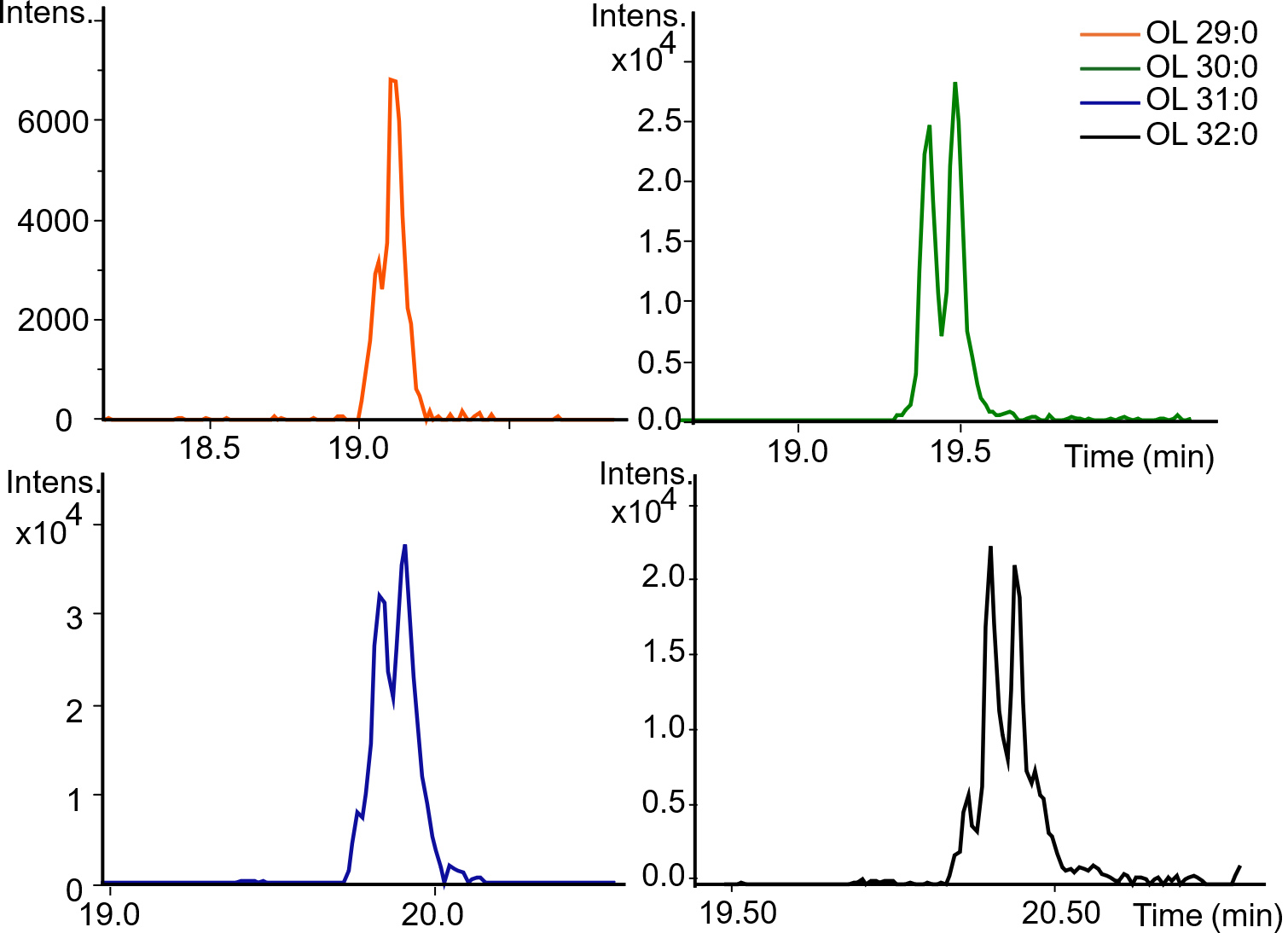


**Figure S5.** Extracted ion chromatograms (EICs) of OLs detected in murine colonic samples: OL 29:0 (*m*/*z* = 581.4899 ± 0.005 Da), OL 30:0 (*m*/*z* = 595.5055 ± 0.005 Da), OL 31:0 (*m*/*z* = 609.5212 ± 0.005 Da), and OL 32:0 (*m*/*z* = 623.5368 ± 0.005 Da), following LC-MS/MS separation on a reversed-phase C8 column in ESI(-) mode.

**
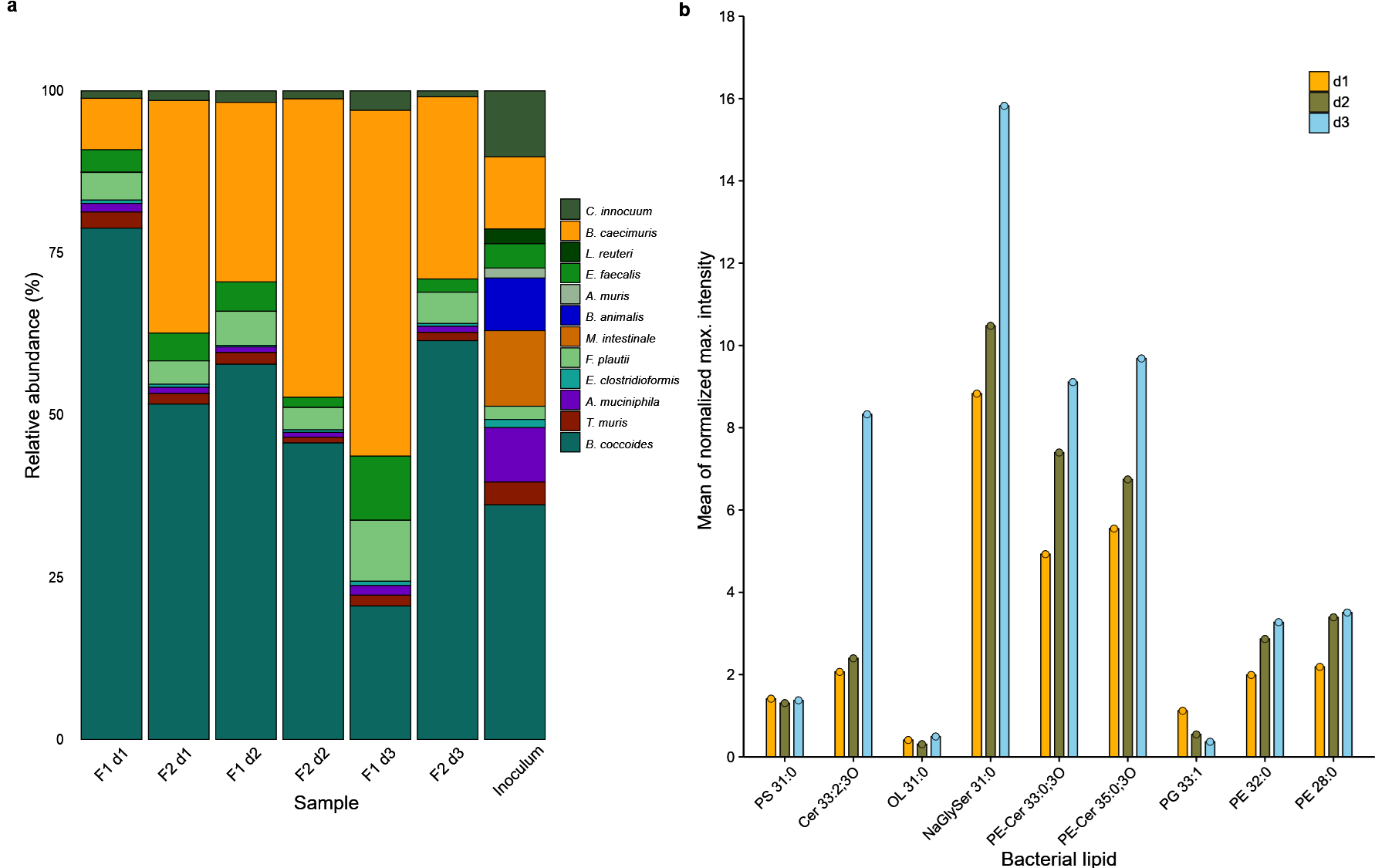
Figure S6.** Community composition and lipidomic profiling of OMM^12^ bacterial co-cultures grown in AF media. (a) Relative abundances of individual bacterial strains within the OMM^12^ community in continuous community culture (two biological replicates, shown separately). Relative abundances were determined by qPCR analysis for the *16S* rRNA gene, conducted in duplicates. Each stacked bar represents the relative abundance of all twelve bacterial strains in each co-culture time point and replicate. (b) Follow-up and examples of bacterial lipid abundances from OMM^12^ co-cultures samples collected at day 1 (d1), day 2 (d2), and day 3 (d3). Data represent the average of normalized maximum peak intensities across replicates for each lipid.

**
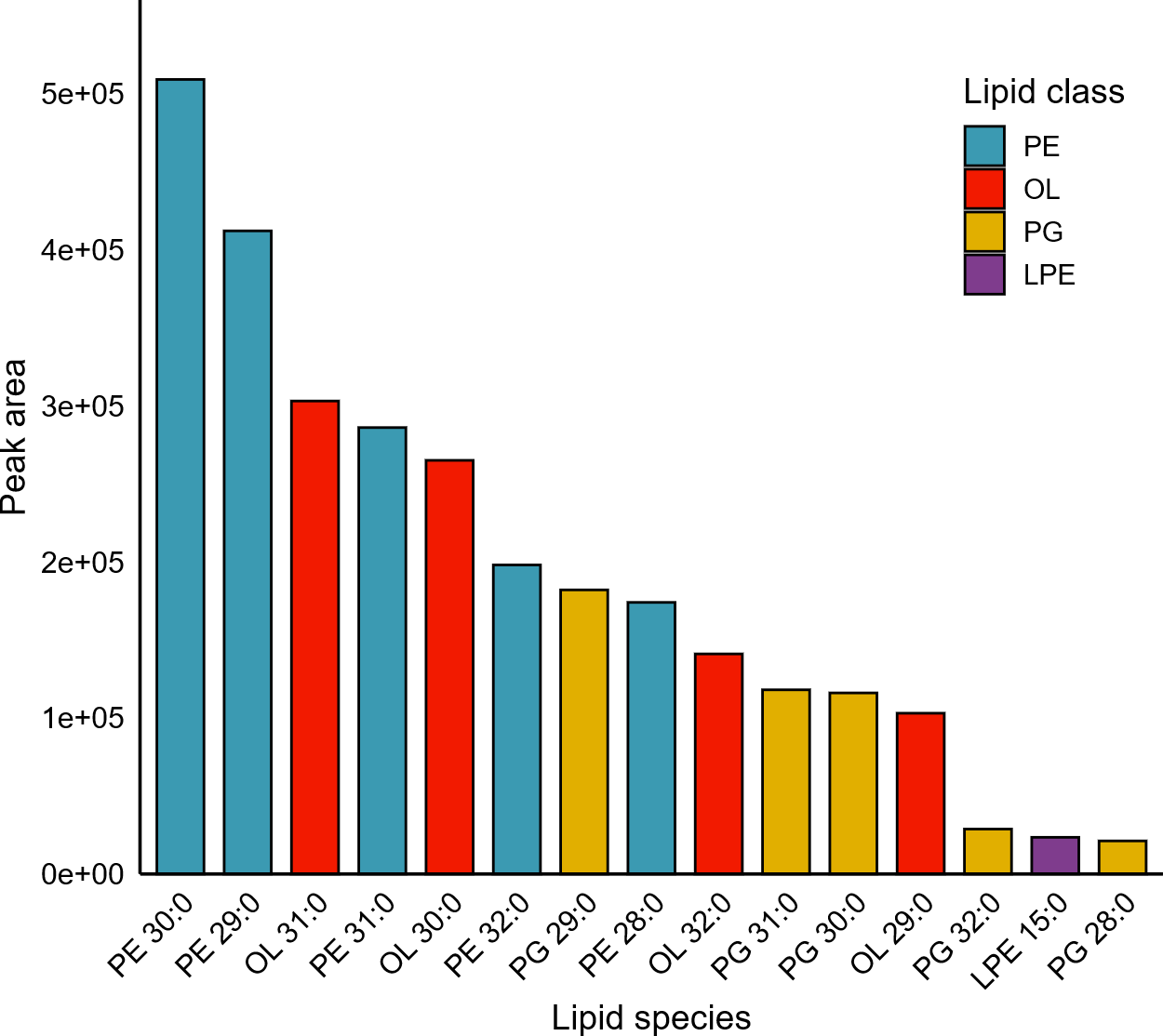
**

**Figure S7**. Abundances of lipid species identified in the *A. muciniphila* cell pellet cultured in AF media. The displayed lipids represent the most abundant species, ranked according to their absolute peak areas.


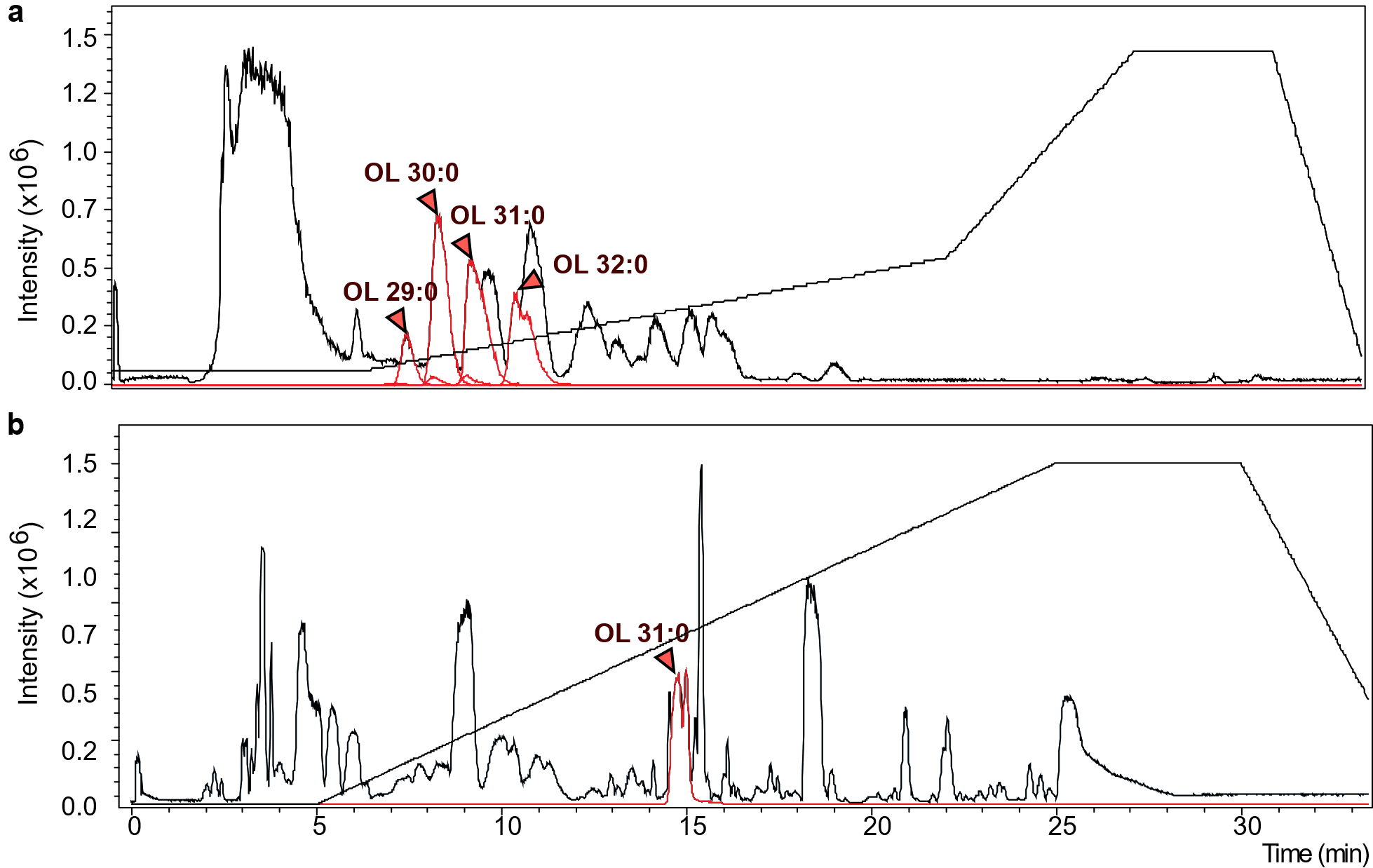


**Figure S8.** Chromatographic separation of a mixture of OLs from *A. muciniphila* pellet. (a) Base peak chromatogram showing the separation of four OLs species: OL 29:0 (*m/z* = 583.5050 ± 0.001 Dalton), OL 30:0 (*m/z* = 597.5203 ± 0.001 Dalton), OL 31:0 (*m/z* = 611.5359 ± 0.001 Dalton), and OL 32:0 (*m/z* = 625.5518 ± 0.001 Dalton) following fractionation on an RP C8 column in ESI(+). The chromatogram highlights the distinct elution profiles of each OL species. (b) Example of base peak chromatogram obtained by HILIC analysis

**
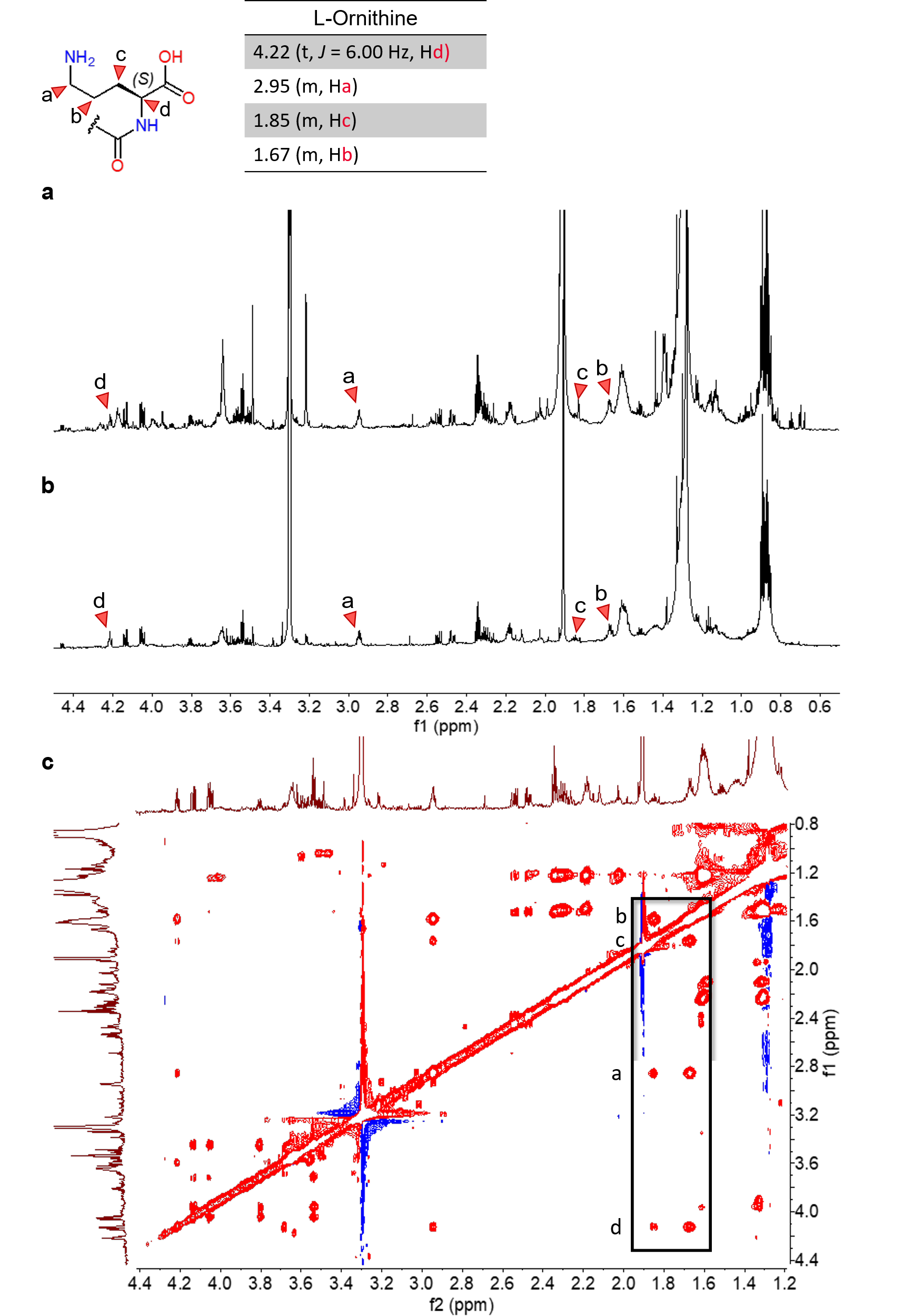
**

**Figure S9.** NMR characterization of isolated OLs from *A. muciniphila* cell pellet. (a, b) ^1^H NMR spectra of OL 30:0 and OL 31:0, respectively, isolated from *A. muciniphila* cell pellet. Spectra were recorded at 800 MHz in CD_3_OD and display characteristic chemical shifts, with annotated functional groups. (c) Phase-sensitive sensitivity-improved 2D TOCSY with WATERGATE and using DIPSI-2 spectrum of *A. muciniphila* OL 31:0, recorded at 800 MHz in CD_3_OD. Characteristic chemical shifts are highlighted by labeled letters.

**
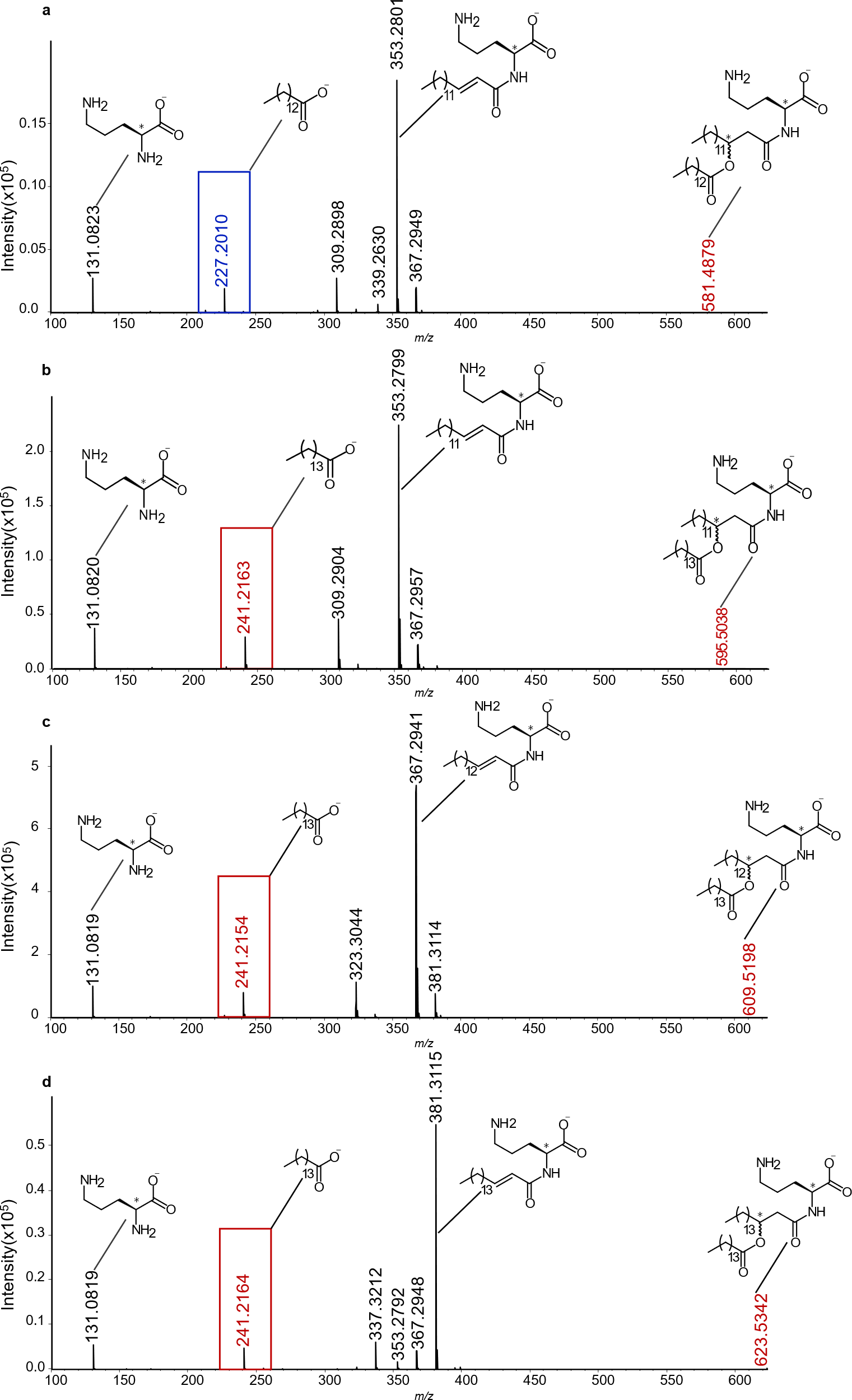
**

**Figure S10.** MS-based characterization of the most abundant OL isomers from *A. muciniphila* cell pellet. (a–d) MS/MS spectrum, acquired in ESI(-), illustrating the structural elucidation of major OL species extracted from *A. muciniphila*. Each panel highlights key fragmentation patterns and characteristic ions (red frame), defining the molecular structure of the OLs: (a) OL 15:0/14:0, detected by the parent ion at *m/z* 581.4879 ± 0.002 Dalton ([M-H]^-^, C_34_H_65_N_2_O_5_^-^); (b) OL 15:0/15:0, detected at *m/z* 595.5038 ([M-H]^-^, C_35_H_67_N_2_O_5_^-^); (c) OL 16:0/15:0, at *m/z* 609.5198 ([M-H]^-^, C_36_H_69_N_2_O_5_^-^) and (d) OL 17:0/15:0, showing an ion at *m/z* 623.5342 ([M-H]^-^, C_37_H_71_N_2_O_5_^-^).


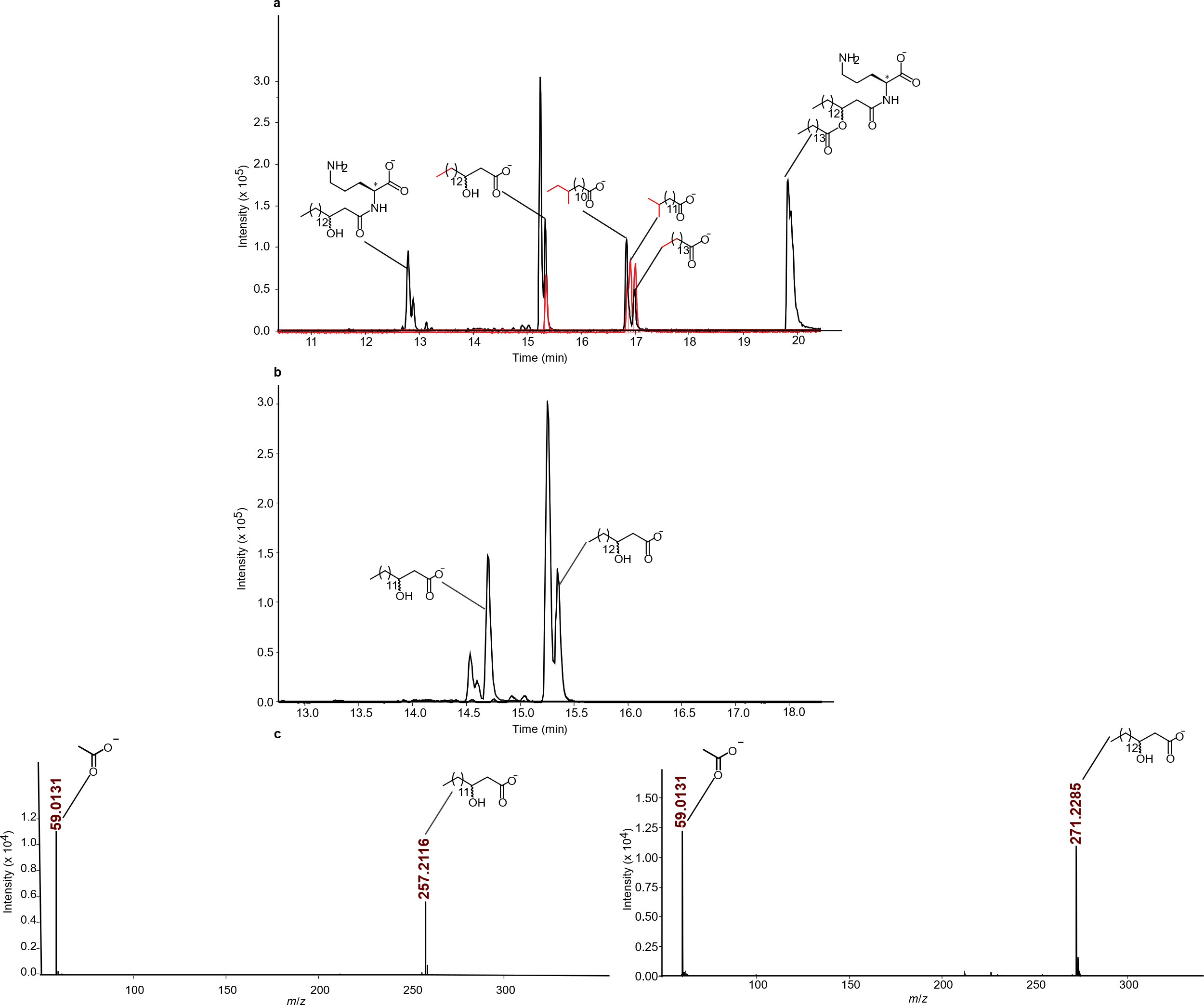


**Figure S11.** Structural identification of FAs and OH-FAs released from OLs isolated from *A. muciniphila* pellet using alkaline hydrolysis. (a) EICs of OL 16:0/15:0 following a two-step alkaline hydrolysis procedure, revealing the release of constituent FAs and OH-FAs. Pentadecanoic acid and 12-methyltetradecanoic acid were identified as the major ester-linked FAs, while straight-chain 3-OH-C16:0 was detected as the amide-linked FA. Validation of these species was confirmed using authentic standards, highlighted in red. (b) EICs illustrate the detection of 3-hydroxypentadecanoic acid and 3-OH-C16:0 following sequential hydrolysis of OL 15:0/15:0 and OL 16:0/15:0, respectively. (c) MS/MS fragmentation patterns of 3-OH-FAs derived from OL 30:0 and OL 31:0, displaying characteristic fragments for structural identification.

**
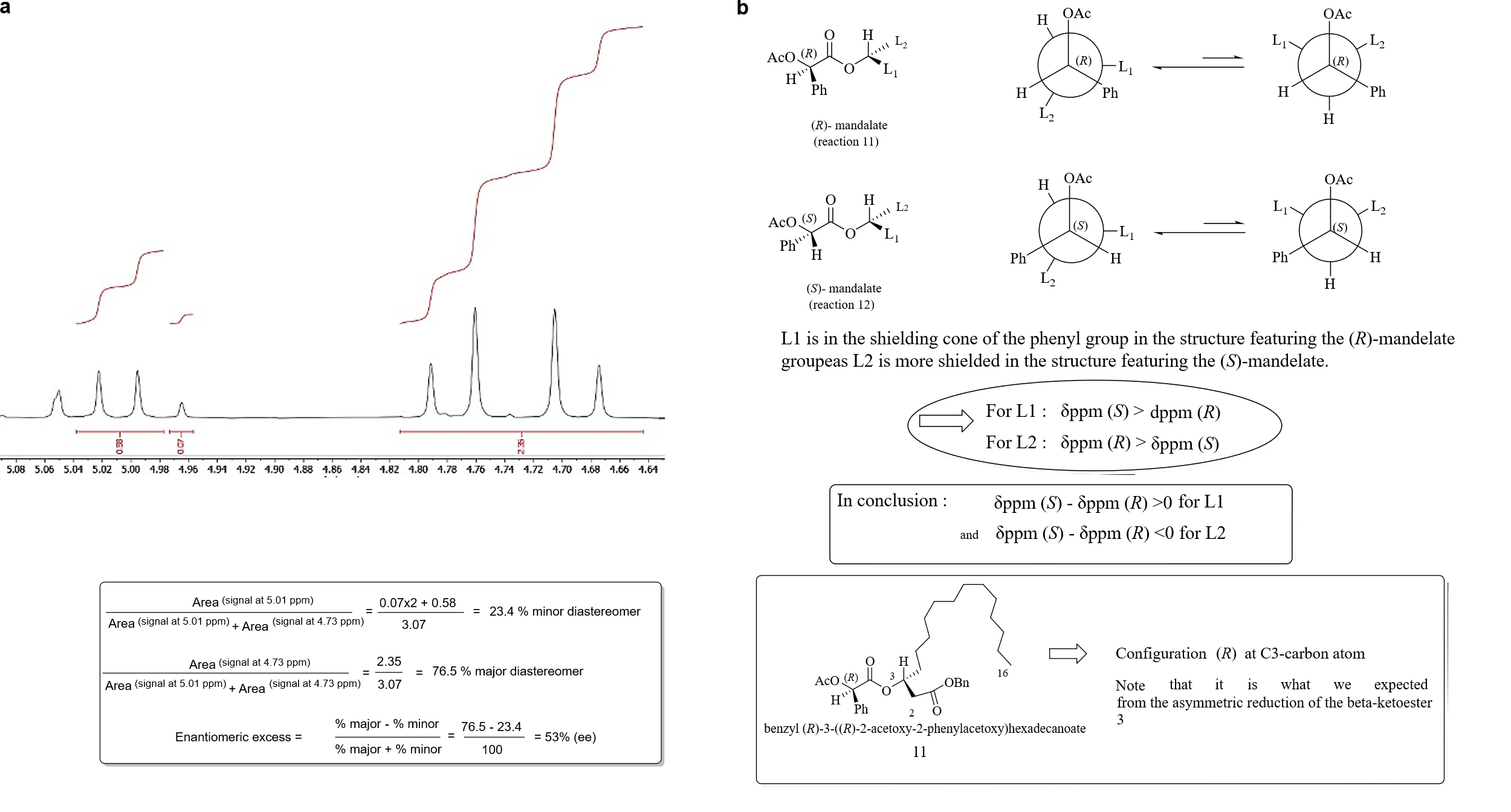
**

**Figure S12.** Determination of ee% and absolute configuration of enriched 3(*R*)-OH-C16:0 (compound 10). (a) Expanded region of the ^1^H NMR spectrum showing the benzylic methylene signals used to determine the diastereomeric excess (dr) of crude compound **11**. Integration analysis indicated, despite a modest stereoselectivity during the Corey–Bakshi–Shibata reduction, a mixture predominantly composed of one diastereomer (77%) and a minor diastereomer (23%), yielding an enantiomeric excess (ee) of 53% for compound 10 *via* ^1^H NMR. Overlapping of ^1^H NMR signals gave an underestimated ee. Chiral HPLC of compound **10** shows a better ee (59%). (b) Assignment of absolute configurations for compounds **11** and **12** based on Δδ values and ^1^H NMR chemical shifts, following the methodology of Chataigner et *al*. (refer to Supplementary Table S4). The benzylic methylene group and H2 protons, α to both the COOBn and secondary alkoxy groups, exhibit greater shielding in the (*R*)-OAc-mandelic acid-derived diastereomer (compound **11**). Since the configuration is preserved during hydrogenation, these findings prove that 3(*R*)-OH-C16:0 is the predominant enantiomer of compound **10**.


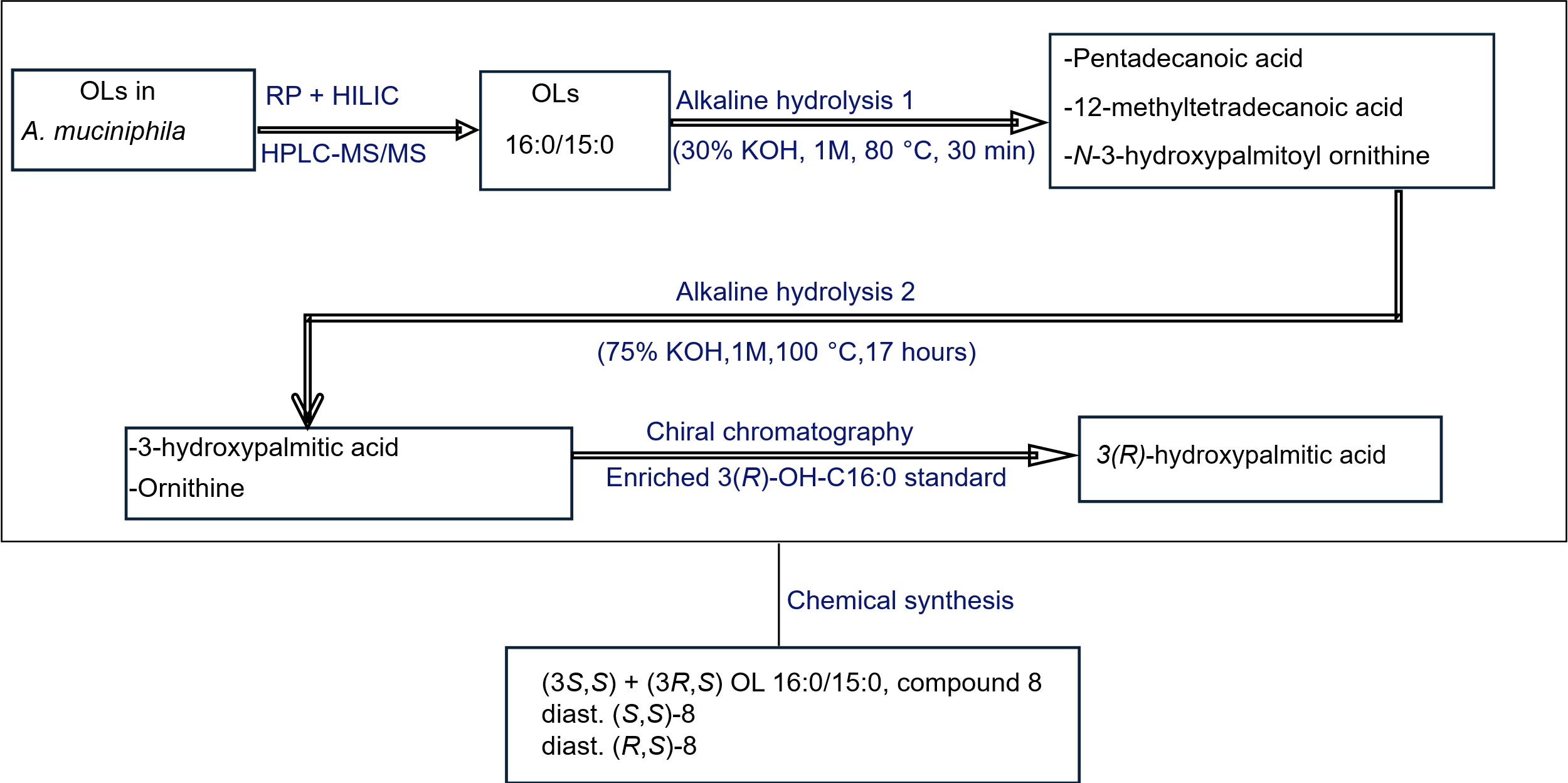


**Figure S13.** Strategy for identification of ester and amide-linked FAs in OL 16:0/15:0 from *A. muciniphila* cell pellet.

**
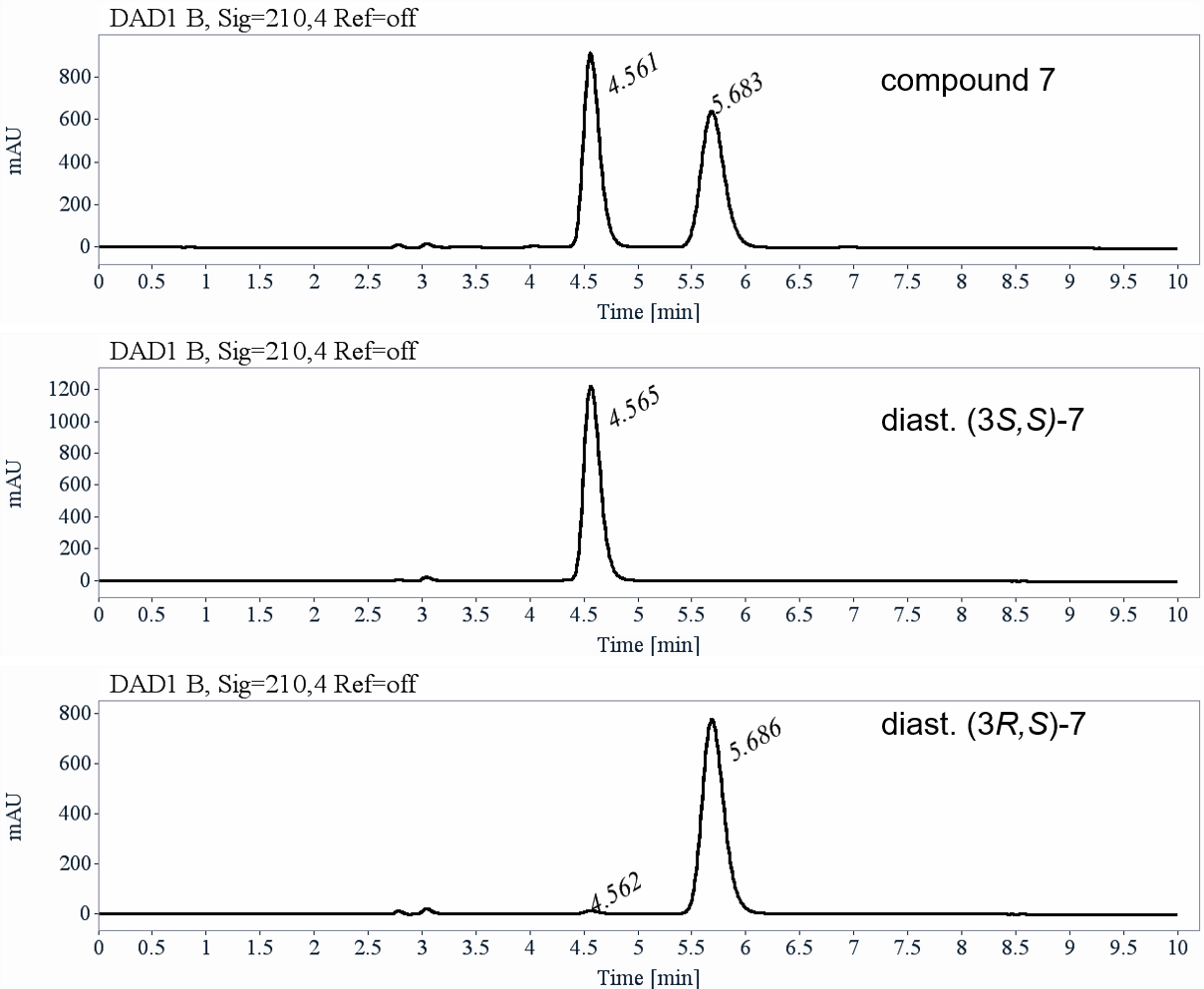
**

**Figure S14.** Chiral preparative HPLC separation of diastereomeric mixture of compound **7** (diast. (3*S,S*)-**7** and (3*R,S*)-7)). UV-Chromatograms on Lux-Cellulose-2 (heptane / ethanol 8:2, 1 mL/min, UV 210 nm) display the separation of the protected OL 16:0/15:0 diastereomeric mixture (Compound **7**) into its enantiopure (3*S,S*)-7 and (3*R,S*)-7 diastereomers and their purity after preparative chromatography.

**
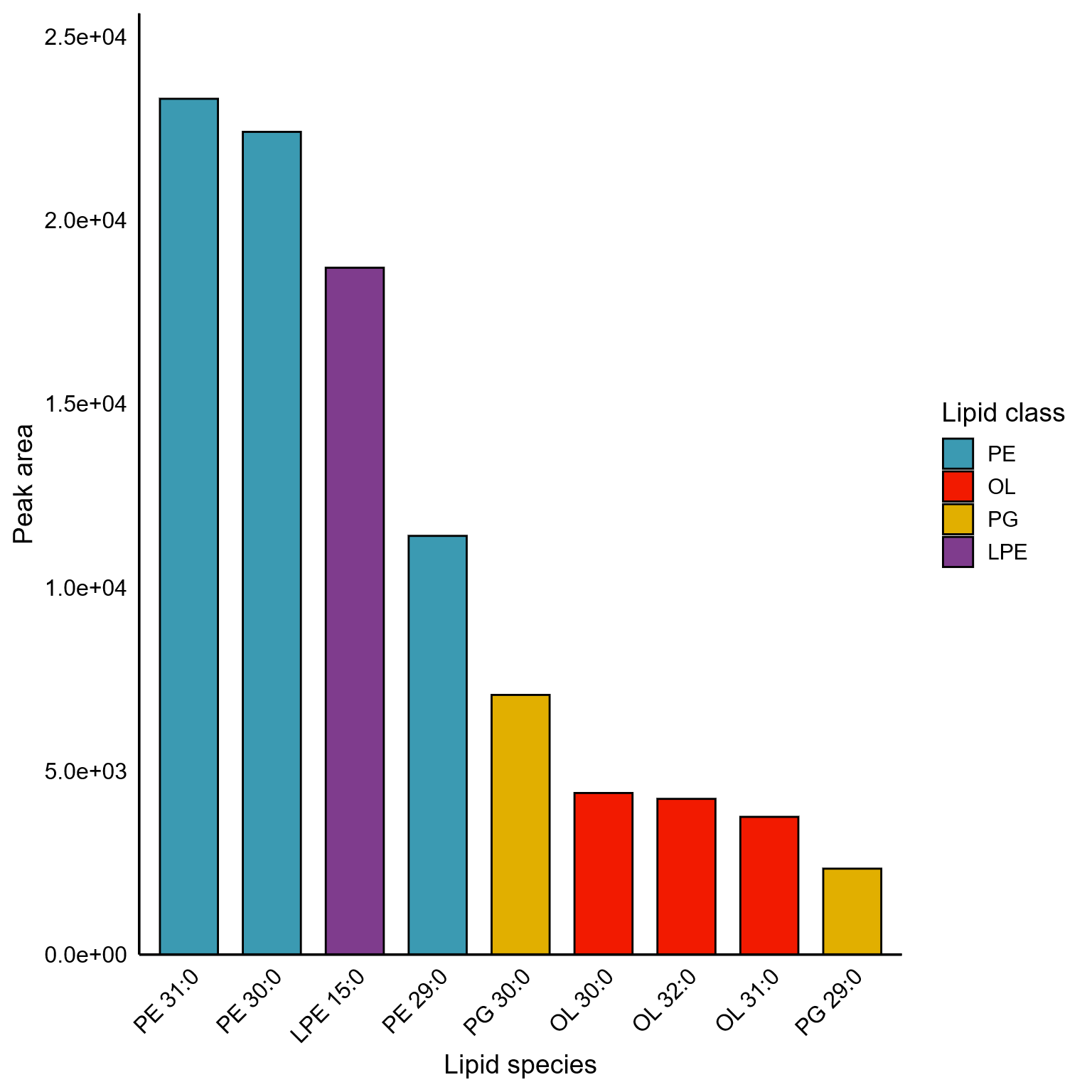
**

**Figure S15.** Examples of lipid species identified in the *A. muciniphila* vesicles cultured in AF media. The displayed lipids represent the most abundant species, ranked according to their absolute peak areas.


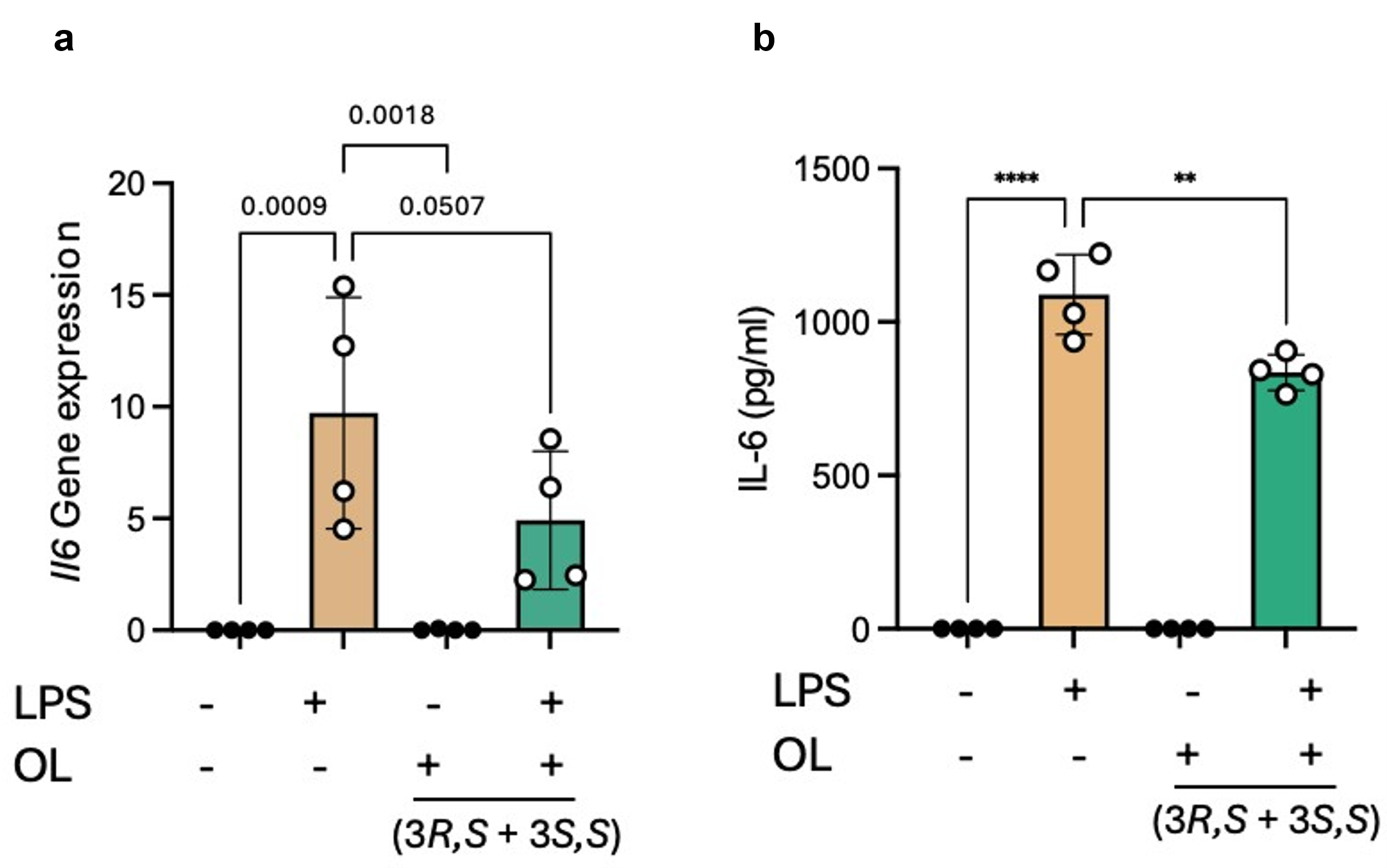


**Figure S16.** Effect of the diastereomeric OL mixture (*3R,S + 3S,S*) on *Il6* mRNA expression (a) and IL-6 protein secretion (b) in control and LPS-stimulated BMDMs.

**
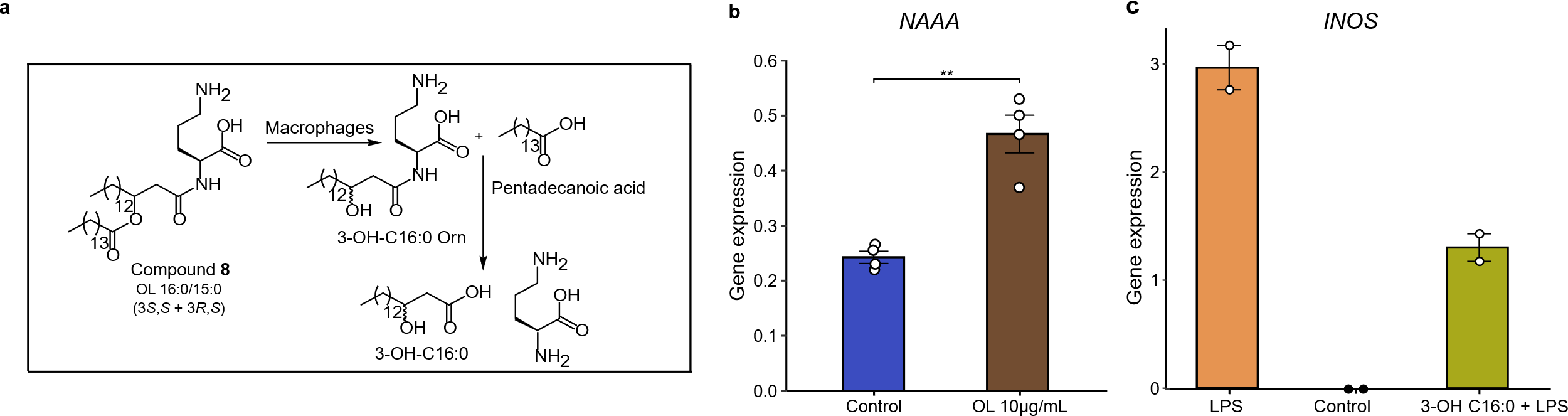
**

**Figure S17.** Functional bioassays of synthetic OL 16:0/15:0 degradation products in BMDMs. (a) Proposed metabolic pathway of OL 16:0/15:0 following uptake by BMDMs, showing enzymatic cleavage of OL into metabolites. (b) *NAAA* gene expression normalized to *Hprt*, in BMDMs treated with synthetic OL 16:0/15:0 (10 µg/mL) for 48 hours compared to untreated controls (DMSO). Data are shown as mean ± SEM from two independent experiments. Statistical analysis was performed using an unpaired two-tailed Student’s *t*-test. (c) Gene expression levels of *INOS* normalized to *Hprt*, measured in LPS-stimulated BMDMs treated with 3-OH-C16:0 (10 µg/mL). BMDMs were pre-treated with 3-OH-C16:0 for 1 hour, followed by stimulation with LPS (10 ng/mL) for 6 hours.

## Supplementary Notes

## Supplementary Note S1. Bacterial strains and growth conditions

Thirteen bacterial strains were used in this study, including *Bifidobacterium animalis* YL2 (DSM26074), *Muribaculum intestinale* YL27 (DSM28989), *Bacteroides caecimuris* I48 (DSM26085), *Enterococcus faecalis* KB1 (DSM32036), *Enterocloster clostridioformis* YL32 (DSM26114), *Acutalibacter muris* KB18 (DSM26090), *Flavonifractor plautii* YL31 (DSM26117), *A. muciniphila* YL44 (DSM26127), *Turicimonas muris* YL45 (DSM26109), *Clostridium innocuum* I46 (DSM26113), *Limosilactobacillus reuteri* I49 (DSM32035), *Blautia coccoides* YL58 (DSM26115), and *Citrobacter rodentium* strain (ATCC51459). Cultures were initiated from frozen monoculture stock solutions in 10 mL volumes and subsequently subcultured in T25 cell culture flasks (Sarstedt). Each bacterial strain was incubated separately at 37 °C under strict anaerobic conditions in an atmosphere of 7% H_2_, 10% CO_2_ and 83% N_2_. To assess the impact of nutrient composition on bacterial lipid production (e.g., OLs from *A. muciniphila*), bacteria were cultured in two media: AF medium^1^, supporting the full bacterial community, and BHI medium, optimized for single-strain growth while eliciting fewer confounding background signals in lipid analysis. The key difference was the presence of heat-inactivated fetal calf serum (FCS) in AF medium, which provides proteins, fatty acids, and bioactive compounds that may influence lipid metabolism. The AF medium, optimized for supporting the growth of all bacterial community members, was composed of 15 g/L trypticase soy broth (Oxoid), 5 g/L yeast extract, 18 g/L glucose-free brain heart infusion (BHI, Oxoid), 0.5 mg/L menadione, 2.5 g/L K_2_HPO_4_, 3% heat-inactivated fetal calf serum (FCS), 1 mg/L haemin, 0.5 g/L D-glucose, and 0.25 g/L cysteine-HCl × H_2_O. The BHI medium contained 37 g/L glucose-free brain heart infusion (BHI, Oxoid), supplemented with the same ingredients as the AF medium, except for FCS. To cultivate the OMM^12^ bacterial community, monoculture inocula were subcultured, diluted in fresh AF medium and mixed in equal ratios. This starting inoculum was transferred to a continuous culture system (Dasgip, Eppendorf, Hamburg, Germany) and supplied with fresh medium with a complete medium turnover time of 2d. Fermenter experiments were maintained for 72 hours, with samples collected every 24 hours for qPCR and LC-MS analysis. DNA extraction and qPCR analysis were conducted following the protocols described by Weiss *et al*.^1^

## Supplementary Note S2. Fractionation, FA identification and estimation of *A. muciniphila* OL concentration

A crude *A. muciniphila* pellet (1 g, OD_600_ = 0.6) was extracted using the extraction method described in the main part. The methanol extract was subjected to sequential fractionation using semi-preparative reversed-phase liquid chromatography (RPLC) and hydrophilic interaction liquid chromatography (HILIC). Fractionation of OLs from *A. muciniphila* was performed using an Agilent 1200 Series HPLC system coupled to a Bruker maXis Quadrupole time-of-flight (QTOF) mass spectrometer (Bruker Daltonics, Bremen, Germany). Initially, RPLC was employed to separate OLs using an Xbridge™ C8 preparative column (5μm, 10 x 100 mm, Waters, Germany). The mobile phase consisted of a mixture of water/ACN (A: 5 millimolar ammonium acetate acidified with acetic acid (0.1%) and B: ACN. The gradient for RPLC started at 85% B for 5.3 min, followed by an initial increase to 90% B over 12.7 min, then to 99% B for 4 min, maintained for an additional 5 min. Reconditioning was conducted for 5 min, followed by a pre-run equilibration time of 2 min with 85% solvent B. 100 μL of sample was then injected into a column heated to 60 °C and analyzed at a flow rate of 2 mL/min for 27 min. Four different OL fractions were collected every 1 min between 5.5 and 9.5 min. The individual OLs obtained from the RPLC fractionation were first evaporated, reconstituted in 75% ACN, and then subjected to purification using an Xbridge™ HILIC column (5μm, 4.6 x 250 mm, Waters, Germany). The separation was carried out using a mobile phase consisting of A: 95% ACN and 5% water and B: water, both with 10 millimolar ammonium acetate. Isocratic conditions were maintained with 1% B for 1 min, followed by a gradient increase to 50% B over 25 min, maintained for 5 min, and subsequently returned to the initial conditions for 5 min. The flow rate was set at 1 mL/min, with a column temperature of 40 °C, injection volume of 100 μL, and separation was conducted for 35 min. Fractions were collected separately every 0.5 min between 14.5 and 15 min. The sample manager was maintained at 4 °C. The MS analyses were performed in ESI(+) in data-dependent mode. Details of MS and MS/MS parameters are provided in Supplementary Table S2. Prior measurements, the MS was calibrated using electrospray tuning mix (Agilent, Santa Clara, CA, USA) in both modes. The ESI tuning mix was injected in the first 0.3 min of each run by switching valve for internal calibration. Purified OL fractions were concentrated using a SpeedVac and reconstituted in 100 μL of methanol-d₃ (CD₃OD) for NMR analysis. ¹H and ¹H-¹H NMR spectra were recorded on a Bruker 800 MHz spectrometer (Bruker Biospin, Rheinstetten, Germany). NMR data were analyzed using Mnova v.14.2.1, and structures were illustrated in ChemDraw v.19.11.32. MS data acquired in .d format was processed with Bruker DataAnalysis (version 5.0).

OL fractions were isolated using RP and HILIC HPLC-MS/MS. Initial analysis was performed on an RP C8 column,^2^ followed by isomer separation using an optimized method on an ACQUITY™ Premier BEH C18 column (150 mm × 2.1 mm, 1.7 μm; Waters)^3^. Targeted analyses used UHPLC (Acquity, Waters, Milford, MA, USA) coupled to a quadrupole time-of-flight MS (maXis, Bruker Daltonics, Bremen, Germany) with mobile phases of 60:40 acetonitrile/water (A) and 90:10 isopropanol/acetonitrile (B), both containing 5 mM ammonium acetate. A gradient (32%–97% B over 25 minutes) was applied at 0.25 mL/min at 40 °C. Ester- and amide-linked FAs were identified through sequential alkaline hydrolysis. Ester-bound FAs were cleaved by incubating OL fractions (~1 µg/mL) in 70 μL methanol with 30 μL of 1 M KOH at 80 °C for 30 minutes, followed by neutralization with 10 μL of formic acid. Samples were analyzed on the RP C8 column in ESI(-) mode using straight- and branched-chain saturated FA commercially available standards (pentadecanoic acid, 13- and 12-Methyltetradecanoic acid). Amide-bound OH-FAs were hydrolyzed by further incubation of the first hydrolysate (70 µL), with 200 µL of 1 M KOH at 100 °C for 17 hours and neutralization by adding 27 µL of formic acid. Hydrolysates were analyzed using LC-MS in ESI(-) mode, with FA and 3-OH-FAs commercially available standards (3-OH-C16:0). Chiral separation of 3-OH-FAs was performed on a Chiralpak IA-U (Daicel) column^4^. Elution order was confirmed using enantiomerically enriched 3(*R*)-OH-C16:0 (compound **10**) alongside hydrolysates from OL 16:0/15:0 fractions and *A. muciniphila* cell pellets. OL 16:0/15:0 concentrations in biological samples were estimated using a synthetic OL standard (Supplementary Table S3, compound **8**).

## Supplementary Note S3. Synthesis of enantiomerically enriched 3(*R*)-OH-C16:0 (compound 10)

Compound **10** was synthesized in two steps, starting from benzyl 3-oxohexadecanoate (compound **3**), as detailed below.

To a solution of (*R*)-(+)-2-methyl-CBS-oxazaborolidine (abbreviated (*R*)-Me-CBS, 209 µL of 1 M solution in toluene, 0.209 mmol, 0.15 equiv) was added borane dimethylsulfide complex (2.16 mL, 1 M in THF, 2.16 mmol, 1.55 equiv). The mixture was stirred for 10 min under a nitrogen atmosphere at rt then cooled to -4 °C. After 10 min stirring, the solution of β-keto ester (compound **3**, 502.7 mg, 1.39 mmol, 1.0 equiv) in anhydrous THF (4 mL) was cannulated dropwise within 45 min at -4 °C to 0 °C. The reaction mixture was stirred for 2h 30min, diluted with ethyl ether (15 mL) and quenched with 2 M HCl (5 mL). The solution was extracted 3 times with ether (15 mL). The combined organic layers were washed with water (5 mL × 3), dried over anhydrous MgSO_4_, and evaporated under reduced pressure. The residue was purified by flash column chromatography (pentane/AcOEt: 9/1). A second flash column chromatography (pentane/AcOEt: 95/5 to 93/7) was necessary to remove traces of impurities that spread all other silica gel. The expected 3-hydroxy ester (compound **9**) was obtained in 74% yield (not optimized, enantiomeric excess (ee) =59% based on HPLC peak area integration of the betaketoacid **10**; ee= 53%, see the method section below and Supplementary Figure S7 for its estimation by ^1^H NMR).

The hydrogenation reaction was conducted following the above-described procedure for the preparation of compound **8**. Briefly, a solution of protected benzylic ester (compound **9**, 150 mg, 0.41 mmol, 1.0 equiv.) in AcOEt/anhydrous EtOH (1v/1v, 4.4 mL) was hydrogenated at rt under atmospheric pressure of hydrogen in the presence of 10% Pd/C catalyst (15 mg, 10% w/w).The expected volume of hydrogen was absorbed and stabilized within ca. 2.5 h The reaction was maintained over hydrogen gas overnight. Upon completion, the reaction mixture was filtered over celite (ca. 1 mL, glass funnel filter with a sintered glass disc, diameter ca. 0.8 cm, porosity 3). The celite was washed with AcOEt, ca. 20 mL). Evaporation of the solvents under reduced pressure afforded the desired carboxylic acid (compound **10**) as a white solid in 81% yield.

To determine the absolute configuration of the synthesized enantiomerically enriched 3-OH-C16:0 (compound **10**) by ^1^H NMR, esterifications of sample **9** were performed with triflylpyridinium (Tf-DMAP) as coupling reagent^5^ and (*R*)- or (*S*)-α-acetoxyphenylacetic acids as chiral derivatizing agents (see details procedure below).

### Mandelate esters with (*R*)-(−)-α-Acetoxyphenylacetic acid (compound 11)

Triethylamine (36 µL, 0.27 mmol, 1.5 equiv., freshly distilled over CaH_2_) was added to a solution of (*R*)-(−)-α-acetoxyphenylacetic acid (35 mg, 0.17 mmol, 1.0 equiv.) and enantiomerically enriched secondary alcohol **9** (51.16 mg, 0.14 mmol, 1.2 equiv.) in dichloromethane (1.2 mL) without special precaution (air). Subsequently, Tf-DMAP (94 mg, 0.22 mmol, 1.3 equiv., prepared according to Chen *et al*.^6^) was added to the reaction mixture. After the reaction was stirred for 15 min, the crude mixture was quenched by water (1.5 mL) and extracted by dichloromethane (3 x 3 mL). The combined organic layers were washed twice with water (1.5 mL), dried over anhydrous MgSO_4_ and filtered over cotton. After the solvent was removed under reduced pressure, the resulting residue was purified by flash column chromatography on silica gel (pentane/ethyl ether: 94/6 to 88/12) to afford the desired esters **11** (diast. (3*R,R*)-**11** (major compound) and diast. (3*R,S*)-**11** (minor compound)). The overall yield was 91%.

### Mandelate esters with (*S*)-(−)-α-Acetoxyphenylacetic acid (compound 12)

The esterification reaction was performed as described above for the synthesis of mandelate (compound **11**), using (*S*)-(+)-α-acetoxyphenylacetic acid instead of (*R*)-(−)-α-Acetoxyphenylacetic acid. This yielded the desired esters **12** as a mixture of two diastereomers, diast. (3*S,R)*-**12** and diast. (3*S,S*)-**12**, in 74% yield (non-optimized).

### Measurement of the enantiomeric excess (ee %) of alcohol 10 *via* ^1^H NMR of diastereomers 11 or 12

¹H NMR analyses were performed on the diastereomeric mixture of crude compound **11** to determine the enantiomeric excess (ee %) of alcohol **10**. In the ¹H NMR spectrum of the crude product obtained from the esterification of secondary alcohol **9** with (*R*)-(−)-α-acetoxyphenylacetic acid (experiment: crude **11**), the benzylic protons of the mandelate esters appear as well-separated signals. However, most of the other proton signals of the diastereomers overlap, precluding accurate integration of the (*R*)- and (*S*)-signals. Thus, the benzylic protons (AB systems at 5.01 and 4.73 ppm for the minor and major diastereomers, respectively) were used to measure the diastereomeric excess (dr) in crude **11** (see Supplementary Figure S7a), which represents the enantiomeric excess of beta-hydroxyester **9** and beta-hydroxyacid **10**. Due to the overlapping of crucial signals in the ^1^H NMR spectra, the deduced ee is underestimated (see the ee observed by chiral HPLC of compound **10**). Analysis of the ^1^H NMR spectra of the diastereomeric mixture **12** gave the same results.

### Assignment of the absolute configuration in compound 9 *via* compounds 11 and 12.

The absolute configuration of the C3 carbon atom in the 3-OH-FA chain was assigned by comparing the chemical shifts of the major diastereomers in esterification crude mixtures (compounds **11** and **12**) prepared with (*R*)- and (*S*)-O-acetyl mandelic acids, following the methodology described by Chataigner et *al*.^7^ (see Supplementary Table S4). According to Chataigner *et al*., a Δ**δ** value greater than 0.05 ppm is considered significant to distinguish configurations. The positive calculated Δ**δ** value shows that the benzylic methylene group and the H2 protons (α to both the COOBn and secondary alkoxy groups) are more shielded in the diastereomer derived from (*R*)-OAc-mandelic acid (experiment **11**) compared to that from (*S*)-OAc-mandelic acid (experiment 12). This indicates the sensitivity of these protons to the phenyl group in the mandelic moiety. According to Chataigner et *al*., the group L1 in the Fisher features the benzylic methylene group and the H2 protons (α to both the COOBn and CHO functions) (see Supplementary Figure S7b). Since the configuration of the benzylated 3-OH-C16:0 (compound **9**) remains unaffected by the hydrogenation reaction. The enantiomerically enriched 3-OH-C16:0 (compound **10**) comprises 3(*R*)-OH-C16:0 as the major enantiomer and 3(*S*)-OH-C16:0 as the minor enantiomer.

## Supplementary Note S4. Enantiomerically enriched 3-HPA characterization (compound 10)

Enantiomerically enriched benzyl (3*R*)-hydroxyhexadecanoate (compound 9)

**Appearance**: white solid

**Rf** = 0.37 (pentane/AcOEt: 90/10, anisaldehyde, UV)

**IR**: 3614-3162 (br OH), 2954, 2914, 2850, 1732 (COOR), 1464, 1455, 1404, 1386, 1327, 1308, 1289, 1267, 1174, 1078, 982, 907, 732, 696 υcm^-1^.

**^1^H NMR** (400 MHz, CDCl_3_): **δppm** 7.42- 7.30 (m, 5H, CH_arom_), 5.15 (s, 2H, CH2O_benzylic_), 4.10-3.94 (m, 1H, CHO), 2.84 (d, *J* = 3.8 Hz, 1H, OH), 2.56 (dd, *J* = 16.4, 3.2 Hz, 1H, CH2 _alpha of C=O_), 2.46 (dd, *J* = 16.5, 8.9 Hz, 1H, CH2 _alpha of C=O_), 1.57-1.36 (m, 3H, CH3), 1.36-1.20 (m, 21H, CH2), 0.88 (t, *J* = 7.1 Hz, 3H, CH3).

Enantiomerically enriched (3*R*)-3-hydroxyhexadecanoic acid (compound 10)

**Appearance**: white solid

**Rf** = 0.50 (CH_2_Cl_2_/MeOH: 90/10, anisaldehyde, No UV)

**IR**: 3533 (OH), 2700 (Br, COOH), 2953, 2913, 2846, 1681 (COOH), 1470, 1443, 1392, 1233, 1221, 1209, 1072, 910, 873, 718 υcm^-1^.

**^1^H NMR** (400 MHz, CDCl_3_): (poorly soluble) **δppm** 4.10-3.95 (m, 1H, CHO), 2.57 (dd, *J* = 16.6 Hz and 3.1 Hz, 1H, CH2 _alpha of C=O_), 2.47 (dd, *J* = 16.6 Hz and 8.9 Hz, 1H, CH2 _alpha of C=O_), 1.62-1.36 (m, 2H, CH2), 1.36-1.71 (m, 22H, CH2), 0.88 (t, *J* = 7.0 Hz, 3H, CH3).

**^1^H NMR** (400 MHz, MeOH*-d4*): 4.03-3.90 (m, 1H, CHO), 2.44 (dd, *J* = 15.2 Hz and 4.9 Hz, 1H, CH2 _alpha of C=O_), 2.36 (dd, *J* = 15.2 Hz and 8.1 Hz, 1H, CH2 _alpha of C=O_), 1.53-1.40 (m, 2H, CH2), 1.40-1.21 (m, 22H, CH2), 0.90 (t, *J* = 7.1, 6.7 Hz, 3H, CH3).

**^13^C NMR** (100 MHz, MeOH*-d4*): **δppm** 175.73 (COOH), 69.36 (CHO), 43.27 (CH2 _alpha of C=O_), 38.13 (CH2), 33.08 (CH2), 30.79 (CH2), 30.73 (CH2), 30.70 (CH2), 30.48 (CH2), 26.66 (CH2), 23.74 (CH2), 14.44 (CH3).

**ESI (negative)** m/z: 271.23 (M-H)^-^, 543.46 (2M-H)^-^.

**HRMS: (ESI)** calculated for C_16_H_31_O_3_ (M-H)^-^ 271.22787, found 271.2278.

Mandelate esters with (*R*)-(−)-α-Acetoxyphenylacetic acid (compound diast. (*R,R*)-11)

**Appearance**: colorless oil

**Rf** = 0.42 (pentane/Et_2_O: 84/16, anisaldehyde, UV); Rf = 0.70 (pentane/AcOEt: 90/10, anisaldehyde, UV).

**IR**: 2923, 2854, 1741 (COOR x3), 1497, 1456, 1372, 1275, 1259, 1229, 1207, 1171, 1051, 1002, 965 υcm^-1^.

**^1^H NMR** (400 MHz, CDCl_3_): 7.51-7.40 (m, 2H, H _arom_), 7.40- 7.28 (m, 6H, H _arom_), 7.26- 7.20 (m, 2H, H _arom_), 5.85 (s, 1H, CHOAc), 5.36-5.22 (m, 1H, CHO _beta of COOBn_), 4.81 (υ_center_ AB system, υ_A_ = 4.86, υ_B_ = 4.77, J_AB_ = 12.3 Hz, CH2O _benzyl group_), 2.52 (υ_A_ in the ABX system observed for CH2 _alpha of C=O_, J_AX_ = 13.7 Hz, J_AB_ = 15.7 Hz), 2.49 (υ_B_ in the ABX system observed for CH2 _alpha of C=O_, J_BX_ = 11.4 Hz, J_AB_ = 15.7 Hz), CH2 _alpha of C=O_), 2.18 (s, 3H, OAc), 1.73-1.50 (m, 4H, CH2), 1.38-1.12 (m, 22H, CH2), 0.88 (t, *J* = 7.1 Hz, 3H, CH3).

**^13^C NMR** (100 MHz, CDCl_3_): **δppm** 170.46 (COOR_mandelic_ _group_), 169.75 (COOBn), 168.27 (CH3COOR), 135.73 (C_quat arom from benzylic group_), 133.87 (C_quat arom in the mandelic group_), 129.27 (CH_arom_), 128.84 (CH_arom_), 128.65 (CH_arom_),128.34(CH_arom_), 127.75 (CH_arom_), 74.81 (CHOAc), 72.29 (CHO _beta of COOBn_), 66.48 (CH2O _benzylic_), 39.14 (CH2 _alpha of C=O_), 34.05 (CH2), 32.08 (CH2), 29.84 (CH2), 29.81 (CH2), 29.78 (CH2), 29.66 (CH2), 29.56 (CH2), 29.51 (CH2), 29.44 (CH2), 25.02 (CH2), 22.84 (CH2), 20.83 (CH3CO), 14.27 (CH3).

**ESI** (positive) *m*/*z*: 479.31 (M-OAc)^+^, 539.34 (M+H)^+^, 556.36 (M+NH_4_)^+^, 561.32 (M+Na)^+^.

HRMS: (ESI) calculated for C_33_H_47_O_6_ (M+H)^+^ 539.33672, found 539.33655; calculated for C_33_H_46_O_6_Na (M+Na)^+^ 561.31866, found 561.31866.

Mandelate esters with (*S*)-(+)-α-Acetoxyphenylacetic acid (major compound diast. (*S,R*)-12)

**Appearance**: colorless oil

**Rf** = 0.27 (pentane/Et_2_O: 84/16, anisaldehyde, UV); Rf = 0.62 (pentane/AcOEt: 90/10, anisaldehyde, UV).

**IR**: 2923, 2854, 1741 (COOR x3), 1497, 1456, 1372, 1275, 1259, 1229, 1207, 1171, 1051, 1002, υcm^-1^.

**^1^H NMR** (500 MHz, CDCl_3_): **δppm** 7.48- 7.41 (m, 2H, H _arom_), 7.39-7.30 (m, 8H, H _arom_), 5.85 (s, 1H, CHOAc), 5.31- 5.23 (m, 1H, CHO _beta of COOBn_), 5.09 (υ_center_ AB system, υ_A_ = 5.12, υ_B_ = 5.06, J_AB_ = 12.2 Hz, CH2O _benzyl group_), 2.70 (dd, *J* = 15.6, 7.2 Hz, 1H, CH2 _alpha of C=O_), 2.57 (dd, *J* = 15.6, 6.0 Hz, 1H, CH2 _alpha of C=O_), 2.18 (s, 3H, OAc), 1.52- 1.38 (m, 2H, CH2), 1.38- 1.15 (m, 18H, CH2), 1.15-1.01 (m, 4H, CH2), 0.88 (t, *J* = 7.1 Hz, 3H, CH3).

**^13^C NMR** (125.8 MHz, CDCl_3_): **δppm** 170.35(COOR_mandelic_ _group_), 170.00 (COOBn), 168.38(CH3COOR), 135.81(C_quat arom from benzylic group_), 133.96 (C_quat arom in the mandelic group_), 129.35 (CH_arom_), 128.84 (CH_arom_), 128.68 (CH_arom_), 128.53 (CH_arom_), 128.42 (CH_arom_), 127.77 (CH_arom_), 74.69 (CHOAc), 72.08 (CHO _beta of COOBn_), 66.72 (CH2O _benzylic_), 39.33 (CH2 _alpha of C=O_), 33.83 (CH2), 32.07 (CH2), 29.84 (CH2), 29.80 (CH2), 29.76 (CH2), 29.55 (CH2), 29.51 (CH2), 29.46 (CH2), 29.21 (CH2), 24.65 (CH2), 22.84 (CH2), 20.86 (CH3CO), 14.29 (CH3).

**ESI** (positive) *m*/*z*: 479.31 (M-OAc)^+^, 539.34 (M+H)^+^, 556.36 (M+NH_4_)^+^, 561.32 (M+Na)^+^.

**HRMS**: (ESI) calculated for C_33_H_47_O_6_ (M+H)^+^ 539.33672, found 539.33655; calculated for C_33_H_46_O_6_Na (M+Na)^+^ 561.31866, found 561.31866.

## Supplementary Note S5. Synthesis of OLs

### General strategy

A diastereomeric (diast.) mixture of OL 16:0/15:0 (compound **8**) was synthesized using a previously reported strategy^8^, with modified procedures described in detail below. Structural elucidation of synthesized compounds was performed using 1D and 2D NMR and MS. Enantiopure OLs 16:0/15:0 ((3*S,S*)-**8** diast. and (3*R,S*)-**8** diast.)) were obtained *via* preparative HPLC chiral separation of the protected diastereomeric mixture **7**, followed by hydrogenation, as outlined in the methods.

### Materials and general methods for OL synthesis

Unless otherwise stated, all commercially available chemicals were used as received without further purification. Reactions were performed in oven-dried glassware under nitrogen. Flash column chromatography was conducted using Macherey-Nagel silica gel 60 (0.04–0.063 mm). Thin-layer chromatography was performed on precoated aluminum plates (Merck Kieselgel 60 F254) and visualized under UV light, followed by p-anisaldehyde staining and heating at 200 °C. Infrared (IR) spectra were recorded on a Perkin Elmer Spectrum 3 FTIR spectrometer and only partial data are listed. Optical rotations were measured on a Anton Parr 4100 polarimeter (MCP 4100 model) at 20°C. Standard wavelength was the sodium D-line at 589 nm. Concentration (g/mL) and solvents are reported with the determined value.^1^H and ^13^C-NMR spectra were recorded on a Bruker Avance III HD 400 MHz NMR spectrometer equipped with BroadBand Inverse (BBI) probe, a Bruker Avance III 500 MHz NMR spectrometer equipped with a 5 mm 1H/X BBO Helium cryoprobe or a Bruker Avance III 600 MHz NMR spectrometer using TCI Cryoprobe Prodigy®. CDCl_3_ was neutralized by filtration over a column of basic Al_2_O_3_. Chemical shifts are reported in ppm, and coupling constants are reported in Hz. ^1^H NMR resonances are referenced to the residual solvent peak (CDCl_3_, 7.26 ppm) and ^13^C NMR resonances are referenced to the residual solvent peak (CDCl_3_, 77.16 ppm)^9^. NMR signals are represented as: chemical shift, multiplicity (s = singlet, d = doublet, t = triplet, q = quartet, m = multiplet), J coupling, integration, assignments). ^1^H NMR and ^13^C NMR signals were assigned from 2D NMR spectra (COSY-^1^H-^1^H, phase-sensitive HSQC and HMBC).

LC-MS analyses were obtained using an Acquity H-Class (Waters) system equipped with a Kinetex EVO C18 column (1.9 mm particle size, 50 x 2.1 mm, Phenomenex). Mobile phase A consisted of water while mobile phase B was acetonitrile, both containing 0.1% formic acid. The gradient started at 0% mobile phase B and increased to 100% mobile phase B over 3 min. After holding for 0.6 min the gradient returned to 0% B before re-equilibration for 1.4 min, to give a total run time of 5 min. The flow rate was 0.5 mL/min and the eluent was directed to the atmospheric pressure ionization source of an Synapt G2-S (Waters, SN: UEB205) operating in ESI (+/-) mode depending on the sample. Capillary and cone voltage were respectively 3000V and 30V. Source and desolvation temperatures were respectively 450 °C and 140 °C. MS analyses were obtained by infusion of the sample in the ESI source of a Synapt G2-S (Waters, SN: UEB205) mass spectrometer operating in positive or negative mode. Capillary and cone voltage were respectively 3000V and 30V. Source and desolvation temperatures were 250 °C and 100 °C respectively. MS/MS analysis was performed by selecting the precursor ion in the Q-Tof system. CID fragmentation is achieved with a ramp from 15 to 40eV. The molecular mass of obtained fragments is determined by the time-of-flight mass analyzer. All MS data was processed by Masslynx 4.2 software.

### Synthesis of a diastereomeric mixture of OL 16/0/15:0 (compound 8)

Compound **8** was synthesized in seven steps, starting from myristic acid (compound **1**), as detailed below.

To a mixture of myristic acid (compound **1**, 1.0 g, 4.38 mmol, 1.0 equiv.), 1-(3-dimethylaminopropyl)-3-ethylcarbodiimide hydrochloride (EDCI.HCl, 923.36 mg, 4.82 mmol, 1.1 equiv.), and 4-(dimethylamino)pyridine (DMAP, 588.45 mg, 4.82 mmol, 1.1 equiv.) in CH_2_Cl_2_ (5 mL) at room temperature (rt) were added 2,2-dimethyl-1,3-dioxane-4,6-dione (Meldrum's acid, 694.22 mg, 4.82 mmol, 1.1 equiv.). The reaction mixture was stirred for 7 hours (h) at rt. It turned into a bright yellow homogeneous solution. Upon completion, the reaction mixture was diluted with CH_2_Cl_2_ (15 mL) and quenched with 1 M HCl (5 mL). The two layers were separated. The aqueous phase was extracted three times with CH_2_Cl_2_ (15 mL). The combined organic phases were dried over MgSO_4_, filtered over cotton. Evaporation of the solvent under reduced pressure afforded quantitatively the desired myristoyl Meldrum’s acid derivative (compound **2**) as a pale-yellow solid, which was used without further purification in the next step.

To the above-described crude Meldrum’s acid derivative (compound **2**, theoretical 1.18g, 4.38 mmol, 1.0 equiv.) dissolved in anhydrous acetonitrile (10 mL) was added freshly distilled benzyl alcohol (2.3 mL, 21.89 mmol, 5 equiv.). The reaction was refluxed at 95 °C for 17 hours. The conversion was complete. Upon cooling, the reaction mixture was evaporated to dryness and the resulting residue was purified by silica gel flash chromatography (pentane/AcOEt: 99/1 to 98/2 as eluent), yielding the expected benzyl beta-ketoester (compound **3**) as a white powder (99% yield over two steps).

Sodium cyanoborohydride (684 mg, 10.87 mmol, 2.5 equiv.) was added to a solution of beta-ketoester (compound **3**) in EtOH/THF (1v/2v, 14 mL) at 0 °C to 5 °C. Subsequently, HCl 1M was added drop by drop over 30 min. The resulting mixture was stirred at 0 °C for 1 h then 1 h at room temperature (rt). Upon completion, the reaction mixture was extracted three times with CH_2_Cl_2_ (30 mL). The combined organic phases were washed with a saturated aqueous NaCl solution (3 x 10 mL). The resulting aqueous phases were extracted with CH_2_Cl_2_ (20 mL). The organic phases were dried over MgSO_4_, filtered over cotton and evaporated to dryness under reduced pressure to give the expected secondary alcohol (compound **4**, quantitative yield) as a colorless oil that slowly solidifies at rt. Compound **4** was directly used in the next step without further purification.

To a solution of pentadecanoic acid (1.26 g, 5.22 mmol, 1.2 equiv.) and DMAP (929,35 mg, 7.61 mmol, 1.75 equiv.) in anhydrous CH_2_Cl_2_ (20 mL) at 0 °C to 5 °C was added EDCI. HCl under a flux of nitrogen. Alcohol (compound **4**) diluted in CH_2_Cl_2_ (23 mL) was cannulated drop by drop. The reaction mixture was stirred at 0 °C to 5 °C for 5 min then overnight at rt. Upon completion, the reaction mixture was diluted with CH_2_Cl_2_ (30 mL) and quenched with HCl 1M (12 mL). After separation of the two layers, the cloudy organic phase was washed twice with HCl 1M (15 mL) and the resulting milky acid aqueous phase was extracted twice with CH_2_Cl_2_ (30 mL). The combined organic phases were washed twice with a saturated aqueous NaHCO_3_ phase (20 mL), then brine (twice 20 mL). The combined basic phases were extracted with CH_2_Cl_2_ (30 mL). The combined organic layers were dried over MgSO_4_, filtered over cotton. After evaporation under reduced pressure, the resulting residue was purified by flash column chromatography on silica gel (elution with pentane/AcOEt: 98/2 to 97/3), providing the desired 3-FAHFA benzyl ester (compound **5**) in 96% yield as a colorless fluid oil.

A solution of benzylated 3-FAHFA (compound **5**, 2.18 g, 3.71 mmol, 1.0 equiv.) in AcOEt (20 mL) was carefully evacuated just until the solvent began to bubble and back-filled with nitrogen. These two steps were repeated three times. The catalyst (10% Pd on carbon, 218 mg, 10% w/w) was added under nitrogen. The flask was evacuated for a few seconds and back-filled with nitrogen three times before the addition of anhydrous ethanol (20 mL) under nitrogen. The flask was connected to a hydrogenation apparatus with graduated burettes filled with hydrogen gas. The reaction mixture was carefully evacuated just until the solvent began to bubble and back-filled with hydrogen, three times, then stirred under hydrogen at atmospheric pressure. The expected volume of hydrogen gas was absorbed and stabilized within ca. 1.5 h. The reaction was maintained under hydrogen overnight. Upon completion, the reaction mixture was filtered over celite (height = ca. 2.5 cm, glass funnel filter with a sintered glass disc, diameter ca. 3.8 cm, porosity 3). The celite was washed with AcOEt/EtOH : 1/1 (ca. 100 mL). Evaporation of the solvents under vacuum provided the desired 3-FAHFA (compound **6**) as a white solid in 98% yield. The sample was used without further purification.

To a solution of 3-FAHFA (compound **6**, 100.4 mg, 0.20 mmol, 1.2 equiv.), protected L-ornithine (Orn, *L*-Orn(Z)-OBzl.HCl, CAS 63594-37-6, fluorochem, 66.2 mg, 1.17 mmol, 1.0 equiv.), 4-methylmorpholine (NMP, 69.7 µL, 0.63 mmol, 3.75 equiv.) in anhydrous CH_2_Cl_2_ (1.7 mL) was added 2-(1H-benzotriazole-1-yl)-1,1,3,3-tetramethylaminium hexafluorophosphate (HBTU, 111.82 mg, 0.29 mmol, 1.75 equiv.) and 1-hydroxybenzotriazole hydrate (HOBt.xH_2_O, 2.30 mg, 0.017 mmol, 0.1 equiv.) under nitrogen. The pale yellowish reaction mixture was stirred for 4 h at rt. Upon completion, the mixture was diluted with CH_2_Cl_2_ (10 mL) and quenched with HCl 1M (1 mL). After separation of the two layers, the organic phase was washed with HCl 1M (1 mL) and the resulting acid aqueous phase was extracted twice with CH_2_Cl_2_ (4 mL). The combined organic phases were washed with a saturated aqueous NaHCO_3_ phase (1 mL), then brine (twice 2 mL) and dried over MgSO_4_, filtered over cotton. After evaporation under reduced pressure, the resulting residue was purified by flash column chromatography on silica gel (elution with CH_2_Cl_2_/MeOH >99/1 to 99/1). The ^1^H NMR spectrum of the collected fractions (96%) showed traces of impurity. A second flash column chromatography on silica gel provided the expected amide (compound **7**) in 85% yield.

The hydrogenation reaction was conducted following the above-described procedure for the preparation of 3-FAHFA (compound **6**). Briefly, a solution of protected OL (compound **7**, 135.30 mg, 0.16 mmol, 1.0 equiv.) in AcOEt/anhydrous EtOH (1v/1v, 6.4 mL) was hydrogenated at rt under atmospheric pressure of hydrogen in the presence of 10% Pd/C catalyst (13.55 mg, 10% w/w). The expected volume of hydrogen was absorbed and stabilized within ca. 2.5 h. The reaction was maintained over hydrogen gas overnight. Upon completion, the reaction mixture was filtered over celite (ca. 1 mL, glass funnel filter with a sintered glass disc, diameter ca. 0.8 cm, porosity 3). The celite was washed with CH_2_Cl_2_/MeOH/hexane: 2/1/0.5 (ca. 100 mL). Evaporation of the solvents under reduced pressure afforded the desired OL (compound **8**) as a white solid in 85% yield.

### Synthesis of enantiopure OLs 16:0/15:0 (Compounds 8, diast. (3*S,S*)-8 and (3*R,S*)-8)

Enantiopure OLs 16:0/15:0 were synthesized in two steps, starting from compound **7**. The first step involved the separation of (3*S,S*)-**7** and (3*R,S*)-**7** diastereomers using chiral HPLC. A Lux-Cellulose-2 column (5 µm, 250 × 10 mm) was employed to resolve the diastereomeric mixture (330 mg) dissolved in an ethanol/hexane solution (40:60, 10 mL). Samples (180 µL) were injected at a flow rate of 1 mL/min, with UV detection at 210 nm. Isocratic elution with hexane/ethanol (80:20) as mobile phase successfully separated the (3*S,S*) and (3*R,S*) diastereomers, which were collected as distinct fractions (Supplementary Table S5). In the second step, the purified fractions were subjected to hydrogenation, following the procedure described for compound **8**, to yield the enantiopure deprotected OLs 16:0/15:0 (diast. (3*S,S*)-**8** and (3*R,S*)-**8**).

2D NMR experiments, including NOESY and ROESY, were conducted but were insufficient to determine the absolute configuration at the C3 position in both (3*S*,*S*)-**8** and (3*R*,*S*)-**8** epimers. To overcome this limitation, we employed sequential alkaline hydrolysis to release the corresponding 3-OH-C16:0, followed by chiral HPLC analysis. The hydrolysates were then compared to racemic and enantiomerically (*R*)-enriched synthetic standards of 3-OH-C16:0 (compound **10**), enabling the assignment of their absolute configuration.

## Supplementary Note S6. Precursors, synthetic diastereomeric mixture and enantiopure OLs characterization (compound 2 to 12)

**Nomenclature**

OL nomenclature followed the LIPID Maps annotation system, where the first lipid represents the hydroxy fatty acid attached to the amide moieties, and the second denotes the fatty acyl moiety. The separator (/) was used, as the alkyl/acyl composition is known as defined by LIPID Maps conventions. For hydroxylated fatty acids, the OH-FA annotation was applied in accordance with established nomenclature.^10^ Additionally, the abbreviation HPA was used for hydroxypalmitic acid in later sections, following previously reported publications.^11^

5-(1-hydroxytetradecylidene)-2,2-dimethyl-1,3-dioxane-4,6-dione (compound 2)

**Appearance**: pale yellow solid

**Rf** = 0.54 (pentane/AcOEt/AcOH: 80/20/0.01, anisaldehyde or KMnO_4_, UV); 0.45 (pentane/AcOEt: 1/1, anisaldehyde or KMnO_4_, UV).

**IR:** 2917, 2848, 1726 (COOR), 1649, 1578, 1425, 1383, 1301, 1211, 1155, 955, 913 υcm^-1^.

**^1^H NMR** (400 MHz, CDCl_3_):  **δppm** 3.08-3.01 (m, 2H, CH2_allylic_), 1.71 (s, 6H, CH3_acetal_), 1.74-1.63 (m, 2H, CH2), 1.44-1.34 (m, 2H, CH2), 1.34-1.17 (m, 18H, CH2), 0.87 (t, *J* = 7.1 Hz, 3H, CH3).

**^13^C NMR** (100 MHz, CDCl_3_): **δppm** 198.49 (C_quat-OH olefin_), 104.80 (C_quat acetal_), 91.32 (C_quat olefin_), 36.00 (CH2), 32.04 (CH2), 29.79 (CH2), 29.76 (CH2), 29.71 (CH2), 29.56 (CH2), 29.51 (CH2), 29.47 (CH2), 29.37 (CH2), 26.92 (CH3_acetal_), 26.28 (CH2), 22.81 (CH2), 14.24 (CH3). The peaks for the two ester functions were not observed: the delay d1 was too short for the relaxation of these quaternary carbons). However, the IR spectrum showed a strong ester absorbance.

**ESI** (positive) *m*/*z*: 355.2 (M+H)^+^.

**HRMS**: (ESI) calculated for C_20_H_35_O_5_ (M+H)^+^ 355.2479, found 355.2480.

Benzyl 15-methyl-3-oxohexadecanoate (compound **3**)

**Appearance**: white powder

**Rf** = 0.62 (pentane/AcOEt: 94/6, anisaldehyde, UV)

**IR**: 2916, 2849, 1756 (COOR), 1738 (CO), 1717 (enol-COOR), 1471, 1410, 1269, 1162, 1072, 988, 737, 696 υcm^-1^.

**^1^H NMR** (400 MHz, CDCl_3_): **δppm** 7.43-7.28 (m, 5H, CH_arom_), 5.17 (s, 2H, CH2 _benzylic_), 3.48 (s, 2H, CH2 _alpha of C=O and COOR_), 2.50 (t, *J* = 7.4 Hz, 2H, CH2 _alpha of C=O_), 1.61-1.51 (m, 2H, CH2), 1.35-1.19 (m, 20H, CH2), 0.88 (t, *J* = 7.0 Hz, 3H, CH3).

**^13^C NMR** (100 MHz, CDCl_3_): **δppm** 202.87 (C=O _ketone_), 167.24 (COOR), 135.4 (C_quat_ _arom_), 128.75 (CH_arom_), 128.59 (CH_arom_), 128.52 (CH_arom_), 128.30 (CH_arom_), 67.23 (CH2O_benzylic_), 49.38 (CH2_alpha C=O and COOR_), 43.23 (CH2_alpha C=O_), 32.06 (CH2), 29.82 (CH2), 29.79 (CH2), 29.73 (CH2), 29.58 (CH2), 29.49 (CH2), 29.14 (CH2), 23.59 (CH2), 22.83 (CH2), 14.26 (CH3).

**ESI** (positive) *m*/*z*: 343.3 (M-H_2_O+H)^+^, 361.3 (M+H)^+^, 378. 3 (M+NH_4_)^+^, 383.3 (M+Na)^+^.

**HRMS**: (ESI) calculated for C_23_H_35_O_2_ (M-H_2_O+H)^+^ 343.2632, found 343.2635; calculated for C_23_H_37_O_3_ (M+H)^+^ 361.2737, found 361.2735; calculated for C_23_H_40_ NO_3_ (M+NH_4_)^+^ 378.3003, found 378.3012; calculated for C_23_H_36_NaO_3_ (M+Na)^+^ 383.2557, found 383.2561.

Benzyl 3-hydroxyhexadecanoate (compound **4**)

**Appearance**: colorless oil that slowly solidifies at rt.

**Rf** = 0.33 (pentane/AcOEt: 94/6, anisaldehyde, UV)

**IR**: 3613-3163 (br OH), 2954, 2914, 2850, 1732 (COOR), 1464, 1455, 1404, 1386, 1327, 1308, 1289, 1267, 1174, 1078, 982, 907, 732, 696 υcm^-1^.

**^1^H NMR** (400 MHz, CDCl_3_): **δppm** 7.43-7.29 (m, 5H, CH_arom_), 5.16 (s, 2H, CH2O_benzylic_), 4.10-3.93 (m, 1H, CHO), 2.84 (br s, 1H, OH), 2.56 (dd, *J* = 16.5 and 3.2 Hz, 1H, CH2 _alpha of C=O_), 2.46 (dd, *J* = 16.4 and 9.0 Hz, 1H, CH2 _alpha of C=O_), 1.59-1.47 (m, 1H, CH2), 1.47-1.36 (m, 2H, CH2), 1.36-1.19 (m, 21H, CH2), 0.88 (t, *J* = 7.1 Hz, 3H, CH3).

**^13^C NMR** (100 MHz, CDCl_3_): **δppm** 173.17 (COOR), 135.71 (C_quat_ _arom_), 128.75 (CH_arom_), 128.51 (CH_arom_), 128.39 (CH_arom_), 68.21 (CHO), 66.64 (CH2O), 41.47 (CH2_alpha COOR_), 36.69 (CH2), 32.05 (CH2), 29.82 (CH2), 29.79 (CH2), 29.71 (CH2), 29.79 (CH2), 29.65 (CH2), 29.49 (CH2), 25.59 (CH2), 22.82 (CH2), 14.25 (CH3).

**ES**I (positive) *m*/*z*: 345.3 (M-H_2_O+H)^+^, 363.3 (M+H)^+^.

**HRMS**: (ESI) calculated for C_23_H_39_O_3_ (M+H)^+^ 363.28937, found 363.2894.

Benzyl 3-(pentadecanoyloxy)hexadecanoate (compound **5**)

**Appearance**: colorless fluid oil

**Rf** = 0.52 (pentane/AcOEt: 97/3, anisaldehyde, UV)

**IR**: 2922, 2853, 1738 (COOR), 1464, 1456, 1261, 1163, 1118, 696 υcm^-1^.

**^1^H NMR** (400 MHz, CDCl_3_): **δppm** 7.43-7.28 (m, 5H, CH_arom_), 5.23 (tt, *J* = 7.3 and 5.5 Hz, 1H, CHO), 5.11 (s, 2H, CH2O_benzylic_), 2.69-2.52 (m, 2H, CH2 _alpha of C=O and CHO_), 2.28-2.11 (m, 2H, CH2 _alpha of C=O_), 1.63-1.49 (m, 4H, CH2), 1.41-1.04 (m, 44H, CH2), 0.88 (t, *J* = 7.1 Hz, 6H, CH3).

**^13^C NMR** (100 MHz, CDCl_3_): **δppm** 173.28 (COOR), 170.44 (COOR), 135.90 (C_quat_ _arom_), 128.67 (CH_arom_), 128.47 (CH_arom_), 128.40 (CH_arom_), 70.41 (CHO), 66.57 (CH2O), 39.47 (CH2_alpha of C=O and CHO_), 34.57(CH2), 34.18 (CH2), 32.07 (CH2), 29.84 (CH2), 29.80 (CH2), 29.77 (CH2), 29.68 (CH2), 29.64 (CH2), 29.61 (CH2), 29.50 (CH2), 29.43 (CH2), 29.27 (CH2), 25.25 (CH2), 25.11 (CH2), 22.83 (CH2), 14.25 (CH3).

**ESI** (positive) *m*/*z*: 587.5 (M+H)^+^.

**HRMS**: (ESI) calculated for C_38_H_67_O_4_ (M+H)^+^ 587.5039, found 587.5043.

3-(pentadecanoyloxy)hexadecanoic acid (compound **6**)

**Appearance**: white wax

**Rf** = 0.36 (pentane/AcOEt: 80/20, anisaldehyde, No UV)

**IR**: 3279-2637 (br OH), 2956, 2915, 2848, 1736 (COOR), 1696 (COOH), 1469, 1437, 1407, 1339, 1320, 1239, 1220, 1197, 1164, 1108, 1087, 1053, 929, 924, 909 υcm^-1^.

**^1^H NMR** (400 MHz, CDCl_3_): **δppm** 5.27-5.15 (m, 1H, CHO), 2.62 (υ_A_ in the ABX system observed for CH2 _alpha of C=O_, J_AX_ = 9.7 Hz, J_AB_ = 15.8 Hz), 2.57 (υ_B_ in the ABX system observed for CH2 _alpha of C=O_, J_BX_ = 7.9 Hz, J_AB_ = 15.8 Hz), 2.28 (t, *J* = 7.5 Hz, 2H, CH2 _alpha of COOR in the C15 chain_), 1.71-1.51 (m, 4H, CH2), 1.49-1.06 (m, 44H, CH2), 0.88 (t, *J* = 7.1 Hz, 6H, CH3).

**^13^C NMR** (100 MHz, CDCl_3_): **δppm** 176.45 (COOH), 173.47 (COOR), 70.15 (CHO), 39.10 (CH2_alpha of C=O and CHO_), 34.63 (CH2_alpha of C=O_), 34.14 (CH2), 32.08 (CH2), 29.85 (CH2), 29.82 (CH2), 29.79 (CH2), 29.70 (CH2), 29.65 (CH2), 29.51 (CH2), 29.44 (CH2), 29.28 (CH2), 25.27 (CH2), 25.16 (CH2), 22.84 (CH2), 14.26 (CH3).

**ESI** (negative) *m*/*z*: 495.4 (M-H)^-^ , 991.9 (2M-H)^-^

**HRMS**: (ESI) calculated for C_31_H_59_O_4_ (M-H)^-^ 495.4419, found 495.4418.

3-FA_C15_HPA-Orn(*Z*)-OBzl (compound **7**)

**Appearance**: white solid

**Rf** = 0.59 (pentane/AcOEt: 70/30, anisaldehyde, UV) ; 0.36 (CH_2_Cl_2_/MeOH :96/4, anisaldehyde, UV)

**IR**: 3318 (NH), 3064 (NH), 3036 (NH), 2953, 2916, 2850, 1738 (COOR), 1686 (C=O), 1644, (C=O), 1543 (C=O), 1468, 1456, 1445, 1357, 1287, 1268, 1217, 1150, 1029 υcm^-1^.

**^1^H NMR** (500 MHz, CDCl_3_): **δppm** (Two diastereomers) 7.61-7.32 (m, 10H x2, H_arom_), 6.48 (br d, *J* = 7.7 Hz, 1H, NH _alphaCH_), 6.44 (br d, *J* = 7.8 Hz, 1H, NH _alphaCH_), 5.28-5.14 (m, 3H x2, CH2 _benzyl of COOH alpha_ and CHO), 5.12 (br s, CH2 _benzyl of group Z_), 4.98 (br t, *J* = 6.1 Hz, 1H, NH _group Z_), 4.92 (br t, *J* = 6.2, 0.7 Hz, 1H, NH _group Z_), 4.78-4.58 (m, 1H x2, CHN _alpha of COOBn_), 3.21 (br q, *J* = 6.7 Hz, 2H x2, CH2_NCOO_), 2.64-2.43 (m, 2H x2, CH2 _alpha of CONH and CHO_), 2.32 (t, *J* = 7.5 Hz, 2H x2, CH2 _alpha of COOR in the C15 chain_), 2.31 (t, *J* = 7.2 Hz, 2H x2, CH2 _alpha of COOR in the C15 chain_), 2.04-1.86 (m, 1H x2, CH2), 1.83-1.45 (m, 7H x2, CH2), 1.45- 1.23 (m, 48H x2, CH2), 0.92 (t, *J* = 6.9 Hz, 6H x2, CH3).

**^13^C NMR** (125 MHz, CDCl_3_): **δppm** (Two diastereomers) 173.73 (COOR _FAHFA_), 173.62 (COOR _FAHFA_), 172.14 (COOBn _alpha_), 169.76 (CONH _alpha to CHN_), 169.70 (CONH _alpha to CHN_), 156.61 (NHCO _group Cbz_), 136.71 (Cquat _arom of group Cbz_), 135.32 (Cquat _arom of COOBn alpha_), 128.80 (CH _arom_), 128.70 (CH _arom_), 128.64 (CH _arom_), 128.48 (CH _arom_), 128.22 (CH _arom_), 71.26 (CHO), 67.41 (CH2O _benzyl_), 66.78 (CH2O _benzyl_), 51.97 (CHN _alpha to COOBn alpha_), 51.92 (CHN _alpha of COOBn alpha_), 41.89 (CH2 _alpha of CHO and CONH_), 41.66 (CH2 _alpha of CHO and CONH_), 40.50 (CH2NCOO) 34.66 (CH2 _alpha of COOR in the C15 chain_), 34.63 (CH2), 34.47 (CH2), 34.28 (CH2), 32.07 (CH2), 29.84 (CH2), 29.80 (CH2), 29.69 (CH2), 29.66 (CH2), 29.50 (CH2), 29.45 (CHppm2), 29.31 (CH2), 25.94 (CH2), 25.72 (CH2), 25.38 (CH2), 25.35 (CH2), 25.13 (CH2), 22.83 (CH2), 14.26 (CH3).

**ESI** (positive) *m*/*z*: 835.6 (M+H)^+^.

**HRMS**: (ESI) calculated for C_51_H_83_N_2_O_7_ (M+H)^+^ 835.61947, found 835.61948.

OL 16/0/15:0 diastereomeric mixture (compound **8**)

**Appearance**: white solid wax

**Solubility**: sparingly soluble in CHCl_3_, CH_2_Cl_2_, MeOH, DMSO (< 2mg/mL); not soluble in acetone, acetonitrile), slightly soluble in THF, soluble in CH_2_Cl_2_/MeOH, hexane or CDCl_3_/MeOH: 9:1 (ca. 10 mg/mL)

**Rf** = 0.06 (CH_2_Cl_2_/MeOH: 80/20, anisaldehyde, No UV)

**^1^H NMR** (600 MHz, CDCl_3_/MeOH-*d4*:9/1): **δppm** (two diastereomers) 5.17-5.00 (m, 1H, CHO), 4.08-3.95 (m, 1H, CHN), 3.88-3.59 (m, 4H, OH, NH3, NH), 2.91-2.71 (m, 2H, CH2N), 2.46-2.25 (m, 2H, H_alpha of C=O and CHOR_)), 2.26-2.12 (m, 2H, H _alpha of C=O in C15 FA chain_), 1.88-1.36 (m, 8H, CH2), 1.36-0.87 (m, 44H, CH2), 0.77 (t, *J* = 7.0 Hz, 6H, CH3).

**^13^C NMR** (151 MHz, CDCl_3_/MeOH-*d4*:9/1): **δppm** (two diastereomers) 176.15 (br, COOH), 173.88 (COOR), 173.78 (COOR), 170.06 (CONH), 170.00 (CONH), 71.29 (CHO), 71.10 (CHO), 53.55 (br, CHN), 41.37 (CH2_alpha of C=O and CHOR_), 41.29 (CH2_alpha of C=O and CHOR_), 39.02 (CH2N), 38.95 (CH2N), 34.49 (CH2 _alpha of C=O in C15 FA chain_), 34.44 (CH2 _alpha of C=O in C15 FA chain_), 34.26 (CH2), 34.21 (CH2), 31.86 (CH2), 29.94 (CH2), 29.63 (CH2), 29.59 (CH2), 29.52 (CH2), 29.48 (CH2), 29.36 (CH2), 29.33 (CH2), 29.30 (CH2), 29.27 (CH2), 29.12 (CH2), 29.10 (CH2), 25.15 (CH2), 25.01 (CH2), 24.97 (CH2), 23.31 (CH2), 23.23 (CH2), 22.61 (CH2), 13.96 (CH3).

**ESI** (negative) *m*/*z*: 609.522 (M-H)^-^ , ESI (positive) *m*/*z*: 611.535 (M+H)^+^.

**HRMS**: (ESI negative) calculated for C_36_H_69_N_2_O_5_ (M-H)^-^ 609.5212, found 609.5218; (ESI positive) calculated for C_36_H_71_N_2_O_5_ (M+H)^+^ 611.5363, found 611.5352.

Enantiopure 3-FA_C15_HPA-Orn(*Z*)-OBzl epimers (diast.(3*S,S*)-**7** and diast. (3*R,S*)-**7**)

Compound diast. (3*S,S*)-**7**

**Appearance**: white solid

**Retention time (RT)** = 4.561 min (heptane/ethanol: 80/20) (see chromatogram in Supplementary Figure S9).

**Optical rotation**: [α]_D_ = + 0.7 (20 °C, c = 10 mg/mL in CH_2_Cl_2_)

**^1^H NMR** (600 MHz, CDCl_3_): **δppm** 7.40-7.27 (m, 10H, H_arom_), 6.43 (d, *J* = 7.7 Hz, 1H, NH _alphaCHN_), 5.24-5.10 (m, 3H, CH2 _benzyl of COOH alpha_ and CHO), 5.08 (s, 2H, CH2 _benzyl of group Z_), 4.93 (br t, *J* = 6.0 Hz, 1H, NH _group Z_), 4.66-4.57 (m, 1H, CHN _alpha to COOBn_), 3.17 (q, *J* = 6.6 Hz, 2H, CH2NCOO), 2.49 ( υ_A_ in the ABX system observed for CH2 _alpha of C=O_, J_AX_ = 8.6 Hz, J_AB_ = 14.5 Hz), 2.45 (υ_B_ in the ABX system observed for CH2 _alpha of C=O_, J_BX_ = 6.3 Hz, J_AB_ = 14.5 Hz), 2.27 (t, *J* = 7.6 Hz, 2H, CH2 _alpha to COOR in the C15 chain_), 1.93-1.82 (m, 1H, 1H of CH2 _alpha to CNH_), 1.73-1.47 (m, 8H, CH2), 1.47-1.35 (m, 1H, 1H of CH2 _alpha to CH2N_), 1.34-1.20 (m, 42H, CH2), 0.88 (t, *J* = 7.0 Hz, 6H, CH3).

**^13^C NMR** (151 MHz, CDCl_3_): **δppm** 173.75 (COOR _FAHFA_), 172.16 (COOBn _alpha_), 169.70 (CONH _alpha to CHN_), 156.63 (NHCO, _group Cbz),_ 136.72 (Cquat _arom of group Cbz_), 135.32 (Cquat _arom of COOBn alpha_), 128.81 (CH _arom_), 128.71(CH _arom_), 128.66 (CH _arom_), 128.49 (CH _arom_), 128.23 (CH _arom_), 71.27 (CHO), 67.42 (CH2 _benzyl_), 66.79 (CH2 _benzyl group CBz_), 51.93 (CHN), 41.91(CH2 _alpha to CHO and CONH_), 40.52 (CH2NCOO), 34.64 (CH2 _alpha to COOR in theC15 chain_), 34.48 (CH2), 32.07 (CH2), 29.86 (CH2), 29.83 (Br, CH2), 29.81 (CH2), 29.70 (CH2), 29.67 (br, CH2), 29.51 (Br, CH2), 29.46 (CH2), 29.32 (CH2), 25.73 (CH2), 25.36 (CH2), 25.13 (CH2), 22.84 (CH2), 14.27 (CH3).

Compound diast. (3*R,S*)-**7**

**Appearance**: white solid

**Retention Time (RT)** = 5.683 min (heptane/ethanol: 80/20)

Optical rotation: [α]_D_ = + 0.6 (20 °C, c = 10 mg/mL in CH_2_Cl_2_)

**^1^H NMR** (600 MHz, CDCl_3_): **δppm** 7.42-7.27 (m, 10H, H_arom_), 6.39 (d, *J* = 7.7 Hz, 1H, NH _alphaCHN_), 5.27 – 5.11 (m, 3H, CH2 _benzyl of COOH alpha_ and CHO), 5.08 (s, 2H, CH2 _benzyl of group Cbz_), 4.87 (t, *J* = 6.1 Hz, 1H, NH _group Cbz_), 4.65-4.58 (m, 1H, CHN _alpha to COOBn_), 3.22-3.11 (m, 2H, CH2NCOO), 2.50 (υ_A_ in the ABX system observed for CH2 _alpha of C=O_, J_AX_ = 8.5 Hz, J_AB_ = 14.8 Hz), 2.47 (υ_B_ in the ABX system observed for CH2 _alpha of C=O_, J_BX_ = 7.1 Hz, J_AB_ = 14.8 Hz), 2.27 (t, *J* = 7.5 Hz, 2H, CH2 _alpha to COOR in the C15 chain_), 1.93-1.82 (m, 1H, 1H of CH2 _alpha to CNH_), 1.76-1.55 (m, 8H, CH2), 1.55-1.35 (m, 1H, 1H of CH2 _alpha to CH2N_), 1.35-1.10 (m, 42H, CH2), 0.88 (t, *J* = 7.0 Hz, 6H, CH3).

**^13^C NMR** (151 MHz, CDCl_3_): **δppm** 173.65 (COOR _FAHFA_), 172.14 (COOBn _alpha_), 169.78 (CONH _alpha to CHN_), 156.62 (NHCO, _group Cbz),_ 136.71 (Cquat _arom of group Cbz_), 135.33 (Cquat _arom of COOBn alpha_), 128.81 (CH _arom_), 128.71(CH _arom_), 128.66 (CH _arom_), 128.50 (CH _arom_), 128.24 (CH _arom_), 71.28 (CHO), 67.42 (CH2 _benzyl_), 66.80 (CH2 _benzyl group CBz_), 51.98 (CHN), 41.68 (CH2 _alpha to CHO and CONH_), 40.50 (CH2NCOO), 34.67 (CH2 _alpha to COOR in theC15 chain_), 34.29 (CH2), 32.07 (CH2), 29.85 (CH2), 29.83 (Br, CH2), 29.81 (CH2), 29.71(CH2), 29.67 (br, CH2), 29.51 (Br, CH2), 29.47 (CH2), 29.33 (CH2), 25.95 (CH2), 25.39 (CH2), 25.15 (CH2), 22.84 (CH2), 14.27 (CH3).

Enantiopure OL 16:0/15:0 (3*S*)-epimer (compound diast. (3*S,S*)-**8**)

**Appearance**: white solid

**Optical rotation**: [α]_D_ = + 6.4 (20 °C, c = 2.5 mg/mL in CH_2_Cl_2_/MeOH: 9/1)

**^1^H NMR** (600 MHz, CDCl_3_/MeOH-*d4*:9/1): **δppm** 5.19-5.10 (m, 1H, CHO), 4.14-4.06 (m, 1H, CHN), 3.40-3.03 (m, 4H, OH, NH3, NH), 2.92-2.82 (m, 2H, CH2N), 2.45-2.35 (m, 2H, CH2 _alpha of C=O and CHOR_), 2.22 (t, *J* = 7.2 Hz, 2H, _alpha of C=O in C15 FA chain_), 1.84-1.59 (m, 4H, CH2), 1.59-1.45 (m, 4H, CH2), 1.31-1.13 (m, 44H, CH2), 0.81 (t, *J* = 7.0 Hz, 6H, CH3).

**^13^C NMR** (151 MHz, CDCl_3_/MeOH-*d4*:9/1): **δppm** 175.40 (br, COOH), 173.98 (COOR), 170.29 (CONH), 71.17 (CH0), 53.04 (br, CHN), 41.38 (CH2_alpha of C=O and CHOR_), 39.12 (CH2N), 34.51(CH2 _alpha of C=O in C15 FA chain_), 34.35 (CH2), 31.93 (CH2), 29.70 (CH2), 29.66 (CH2),29.58 (CH2), 29.55 (CH2), 29.42, 29.36 (CH2), 29.32 (CH2), 29.16 (CH2), 25.19 (CH2), 25.02 (CH2), 23.30 (CH2), 22.68 (CH2), 14.06 (CH3).

Enantiopure OL 16:0/15:0 (3*R*)-epimer (compound diast. (3*R,S*)-**8**)

**Appearance**: white solid

**Optical rotation**: [α]_D_ = + 2.4 (20 °C, c = 2.5 mg/mL in CH_2_Cl_2_/MeOH: 9/1)

**^1^H NMR** (600 MHz, CDCl_3_/MeOH-*d4*:9/1): **δppm** 5.15-5.06 (m, 1H, CHO), 4.05-3.97 (m, 1H, CHN), 3.69-3.33 (m, 4H, OH, NH3, NH), 2.89-2.80 (m, 2H, CH2N), 2.47-2.29 (m, 2H, CH2 _alpha of C=O and CHOR_), 2.26-2.12 (m, 2H, _alpha of C=O in C15 FA chain_), 1.79-1.57 (m, 4H, CH2), 1.54-1.47 (m, 4H, CH2), 1.25-1.13 (m, 44H, CH2), 0.79 (t, *J* = 7.0 Hz, 6H, CH3).

**^13^C NMR** (151 MHz, CDCl_3_/MeOH-*d4*: 9/1): **δppm** 176.09 (br, COOH), 173.79 (COOR), 170.07 (CONH), 71.32 (CHO), 53.54 (br, CHN), 41.37 (CH2_alpha of C=O and CHOR_), 38.99 (CH2N), 34.52 (CH2 _alpha of C=O in C15 FA chain_), 34.24 (CH2), 31.90 (CH2), 30.20 (CH2), 29.67 (CH2), 29.63 (CH2), 29.55 (CH2), 29.51 (CH2), 29.36 (CH2), 29.33 (CH2), 29.30 (CH2), 29.15 (CH2), 25.18 (CH2), 25.04 (CH2), 23.34 (CH2), 22.65 (CH2), 14.01 (CH3).

## Supplementary NMR spectra


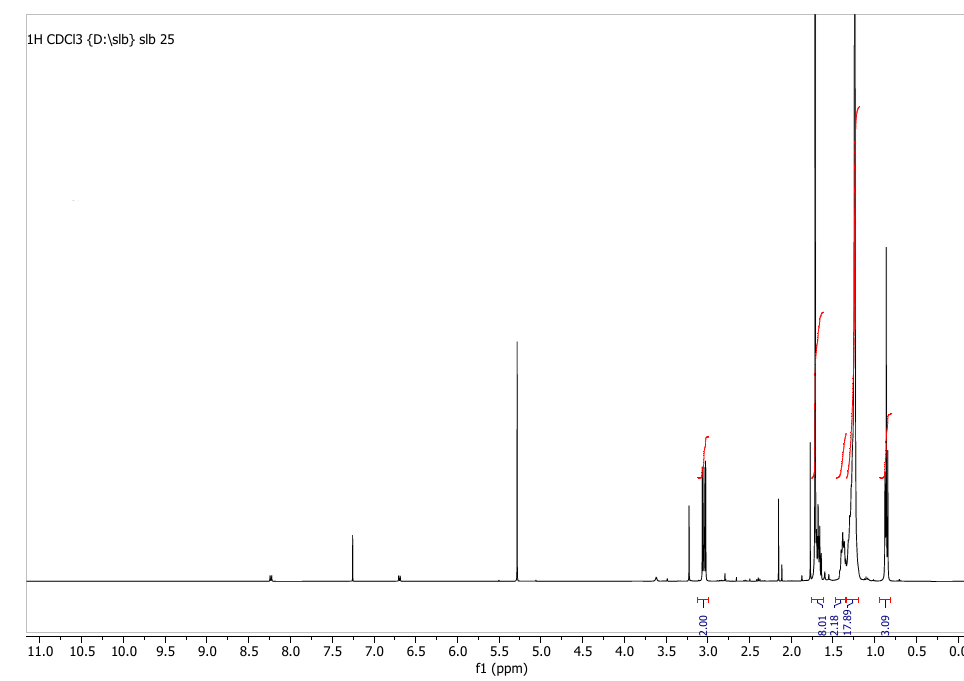

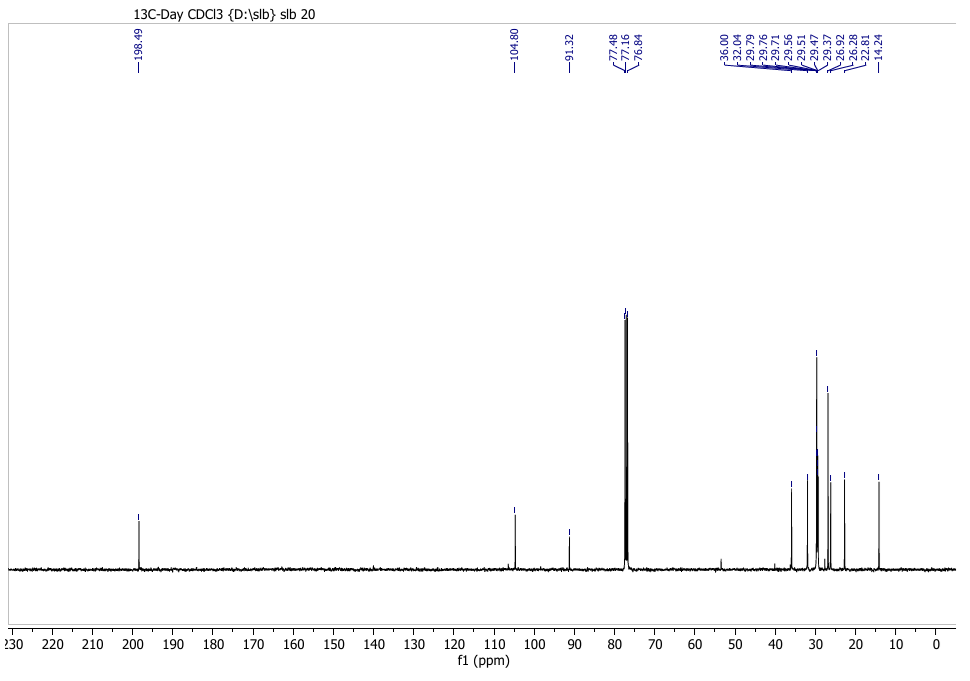

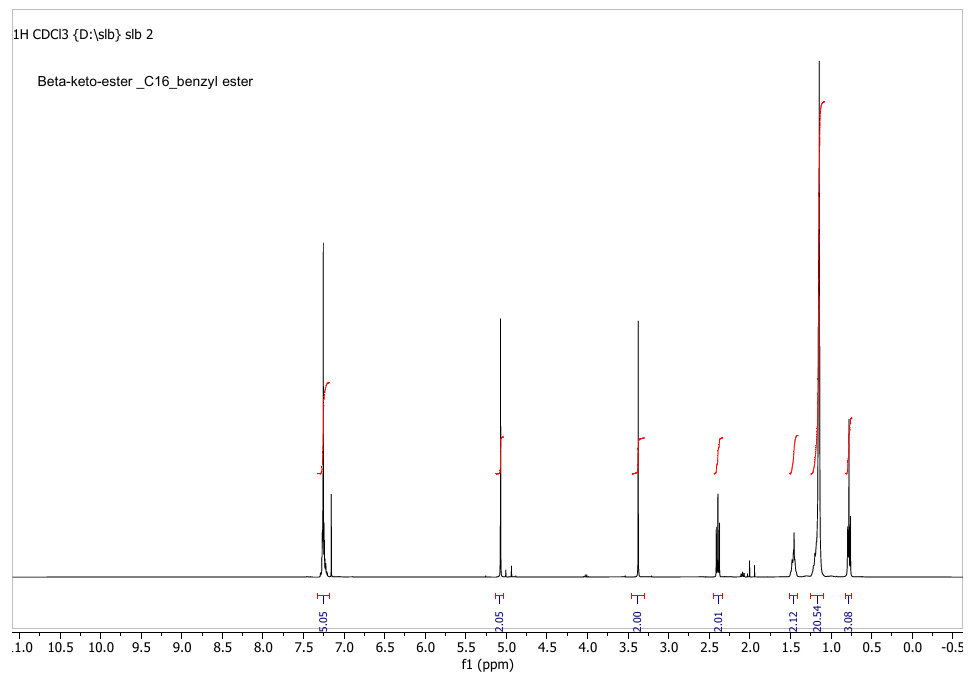

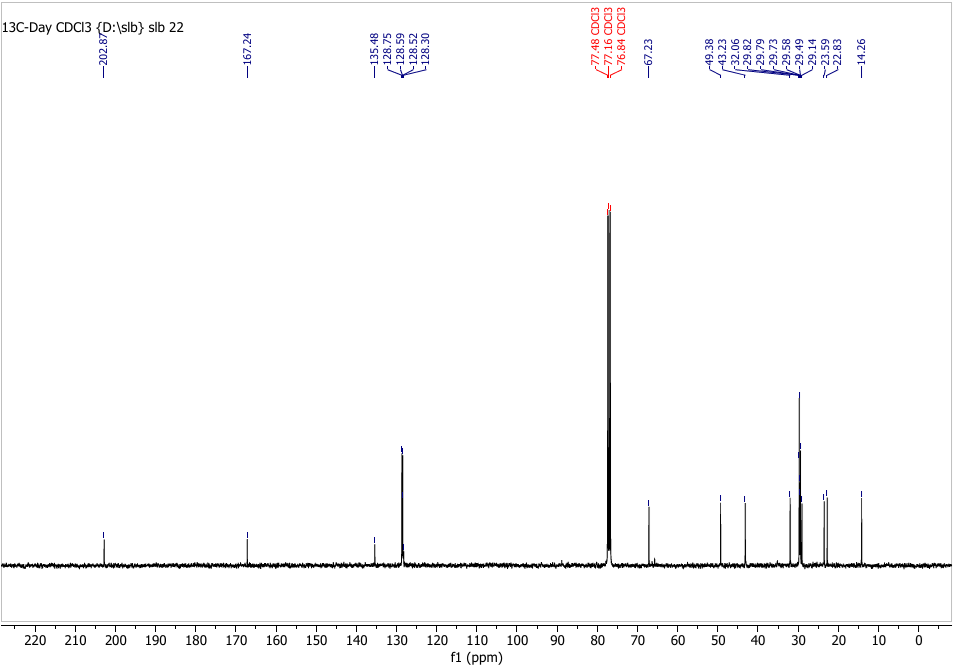

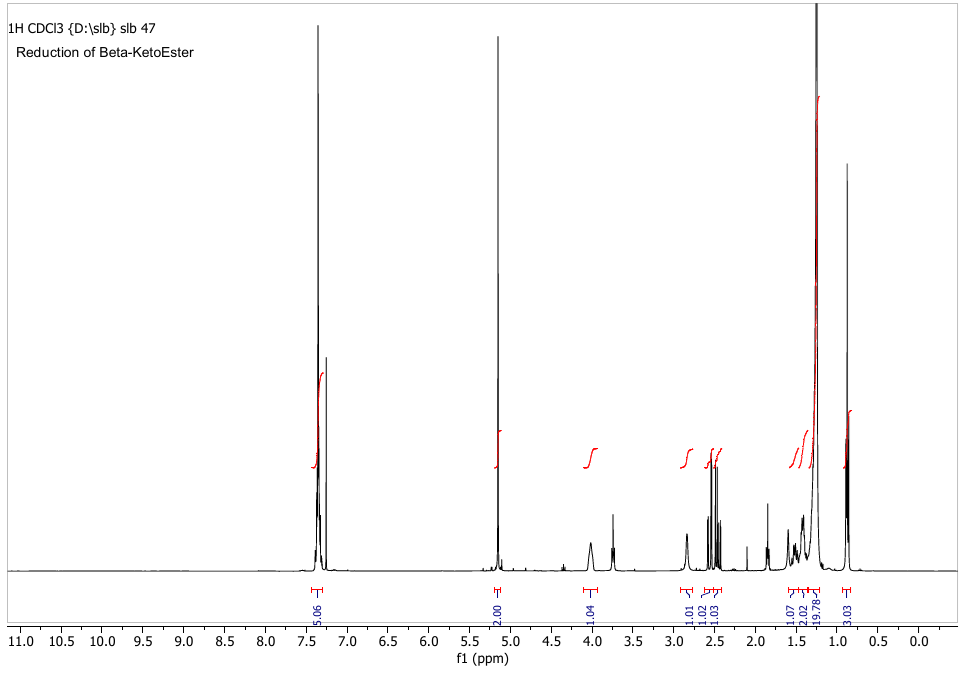

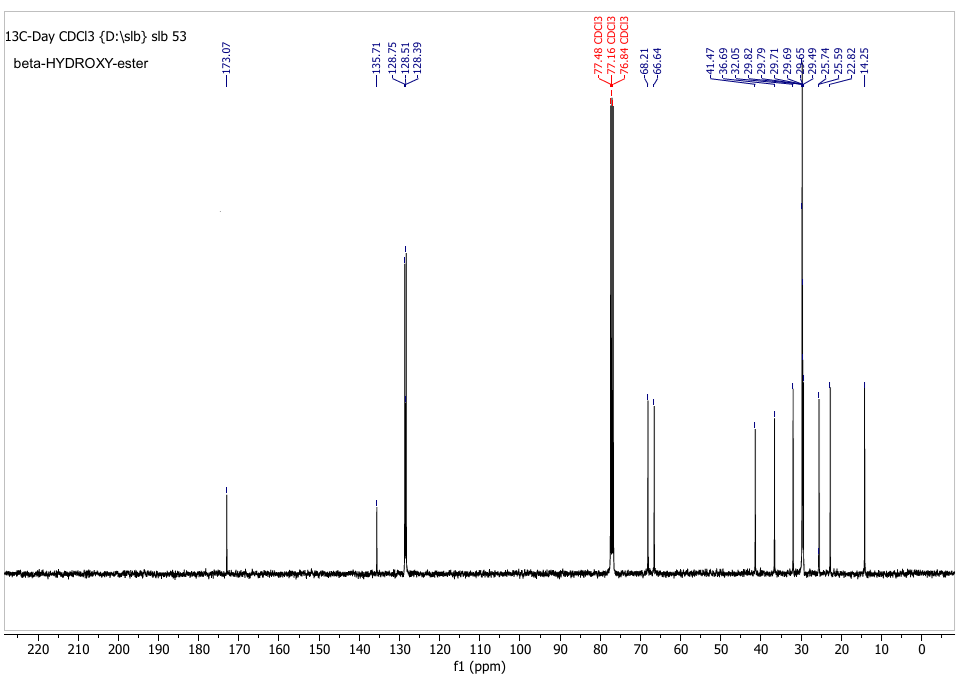

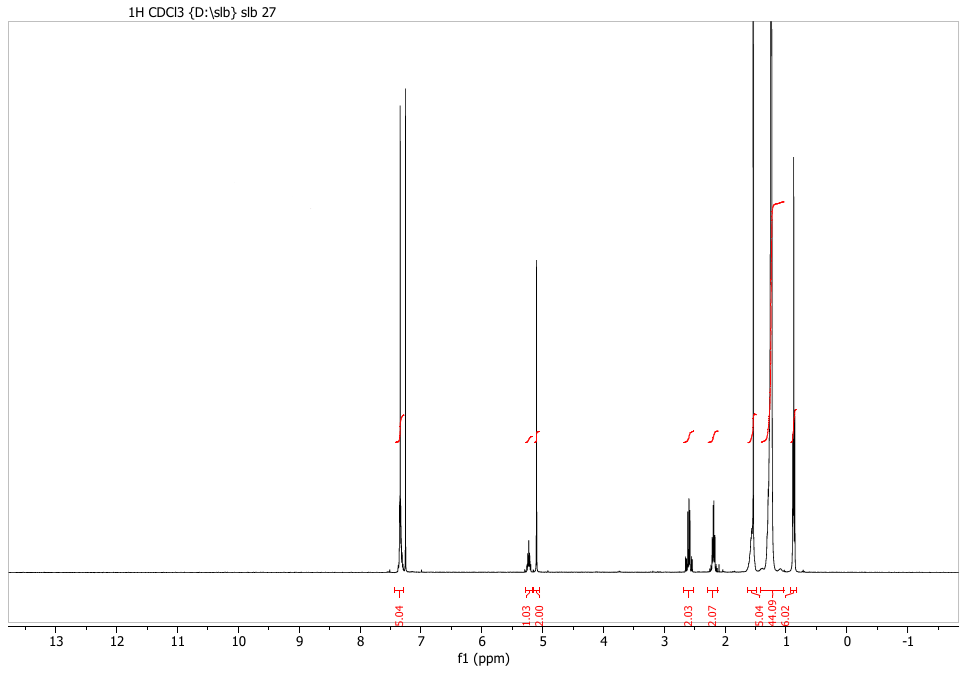

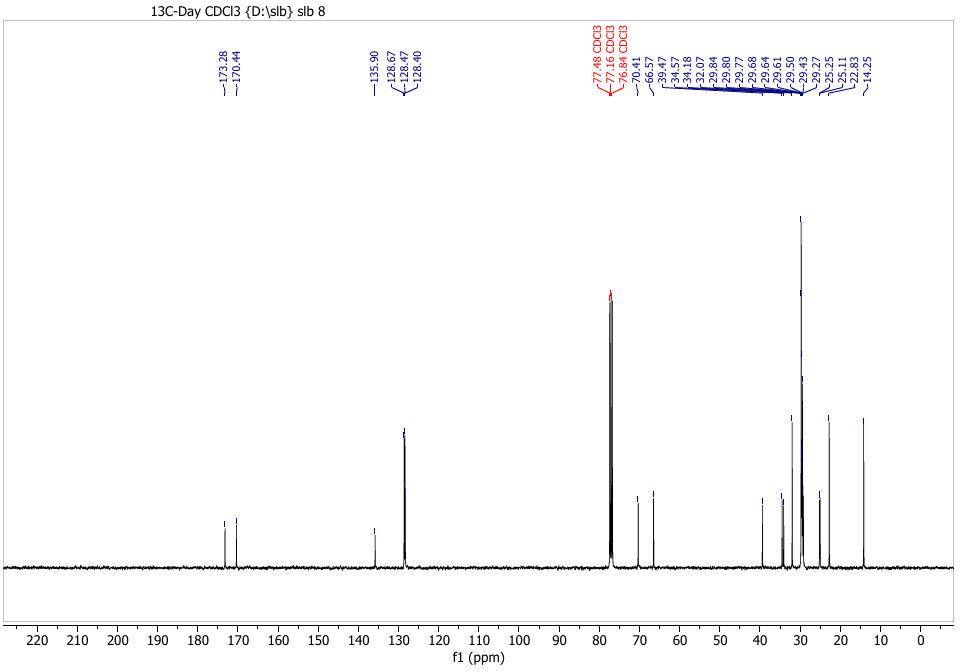

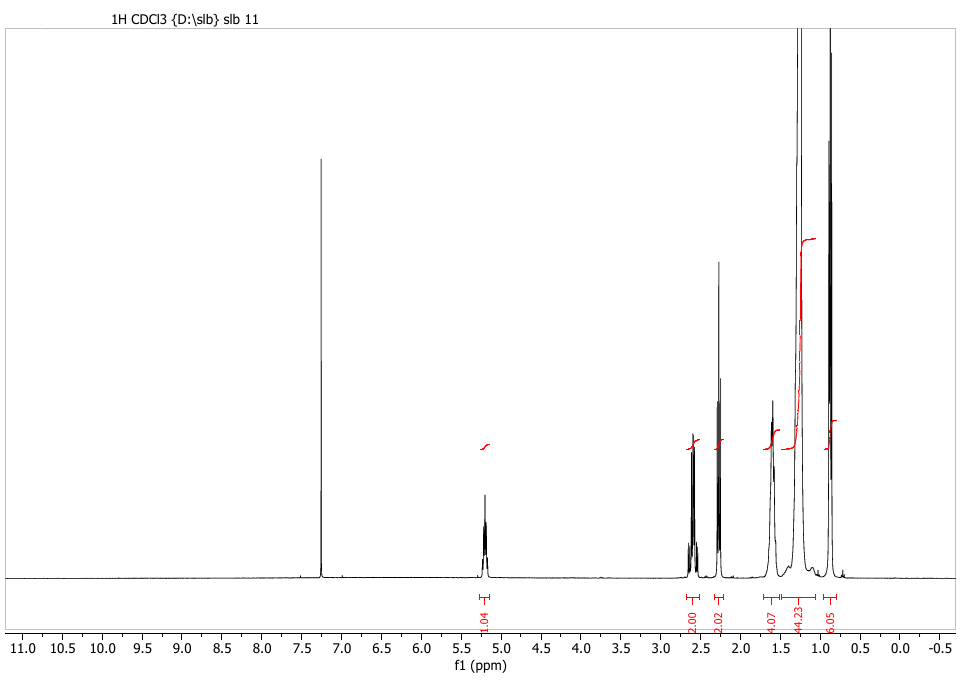

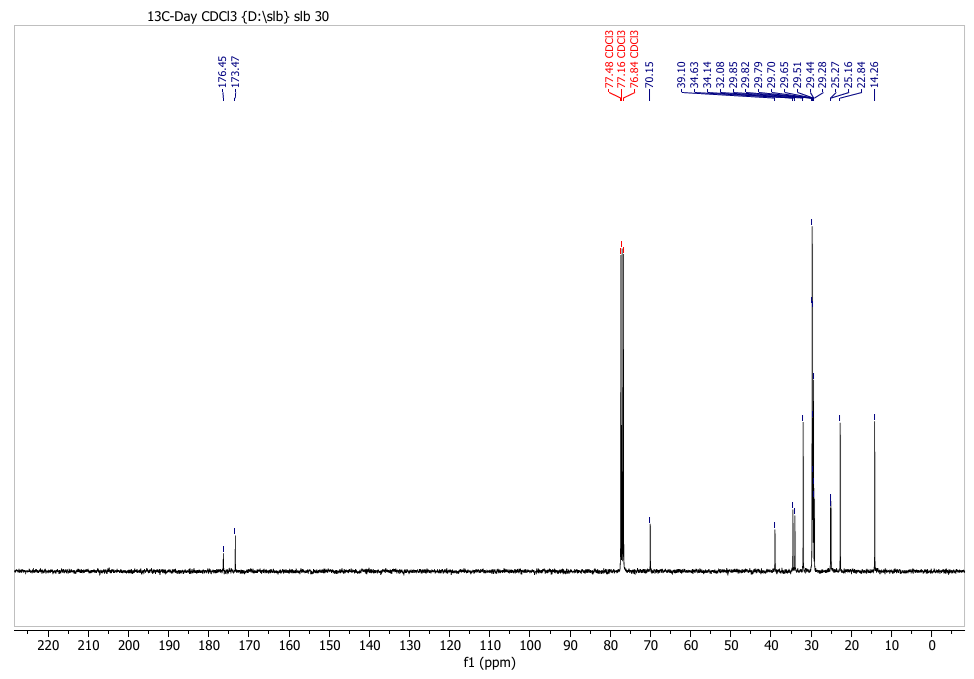

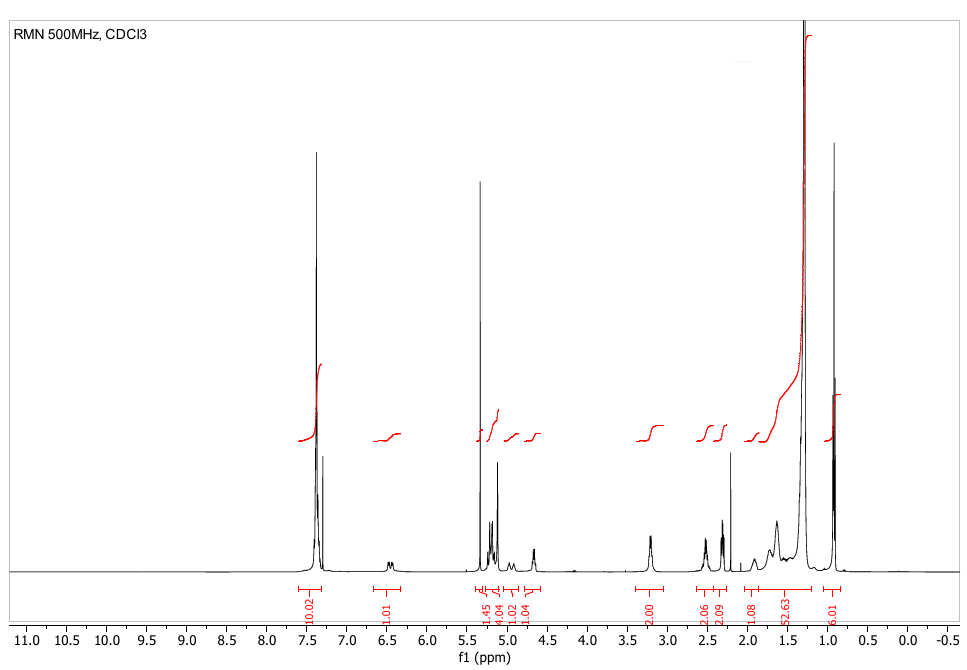

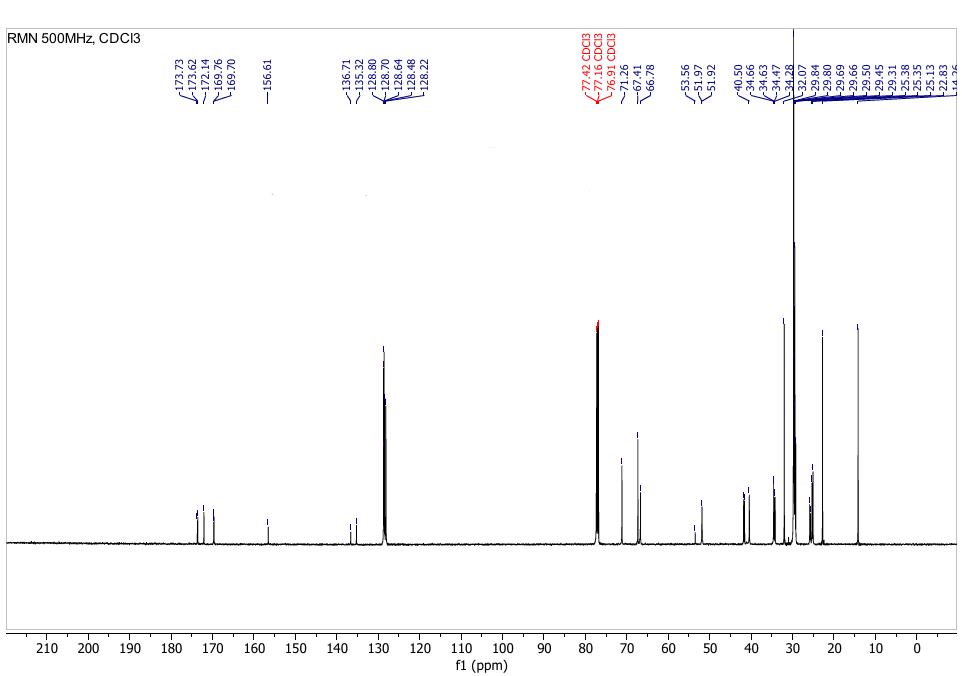


13C NMR, 125 MHZ, CDCl3


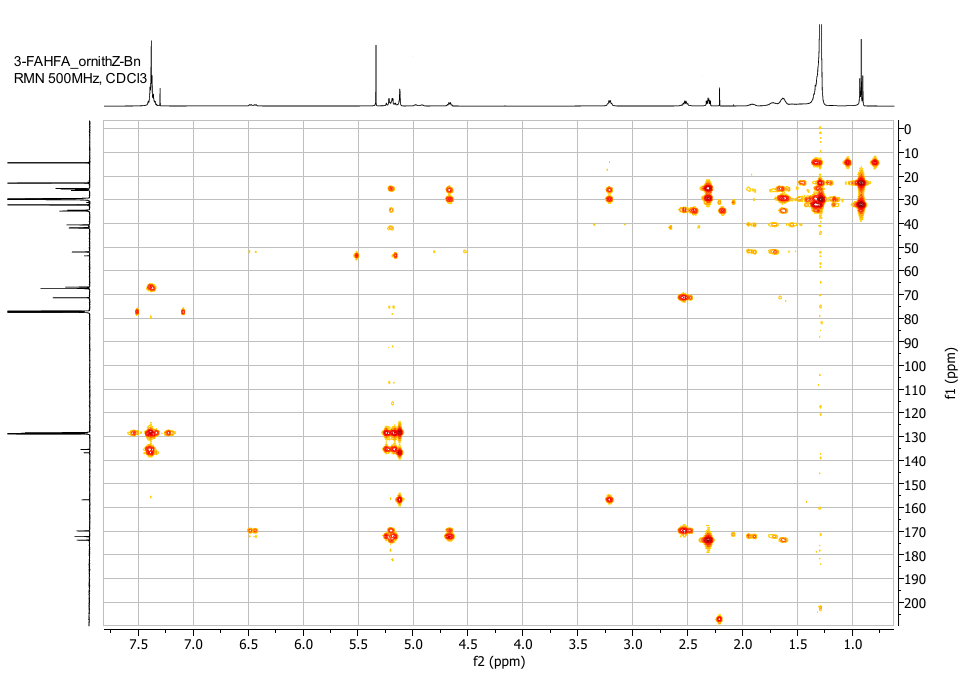


HMBC

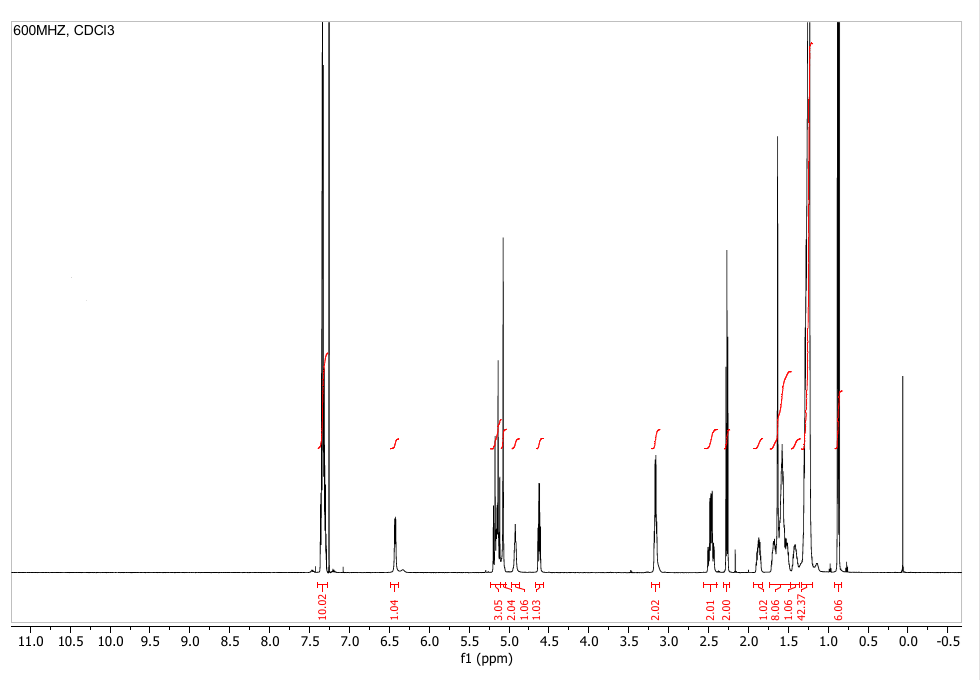

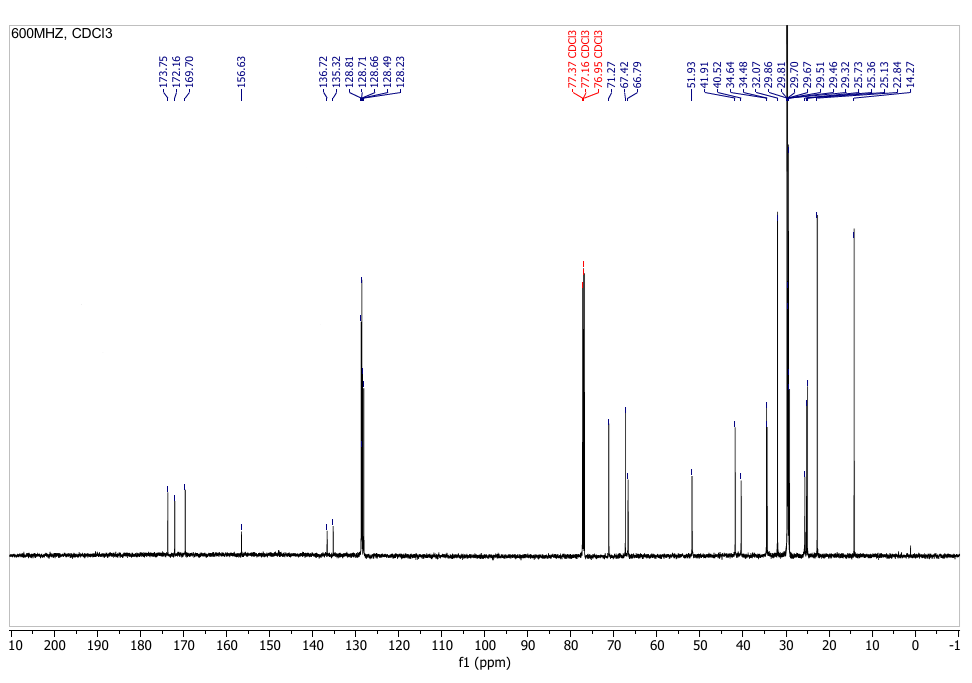


13C- CDCl3

13C NMR, 125 MHZ, CDCl3


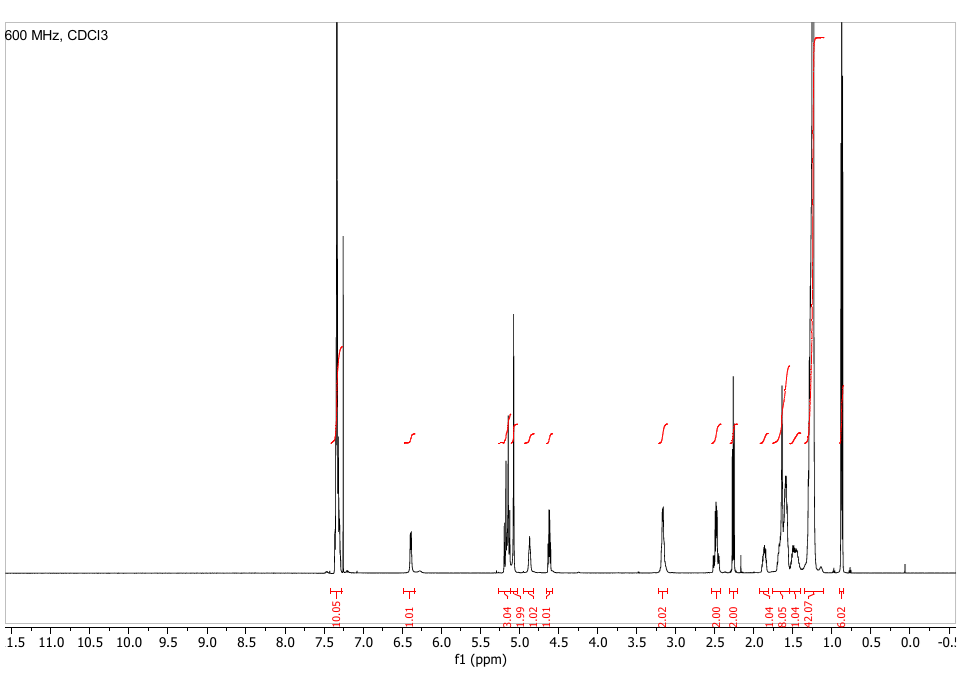

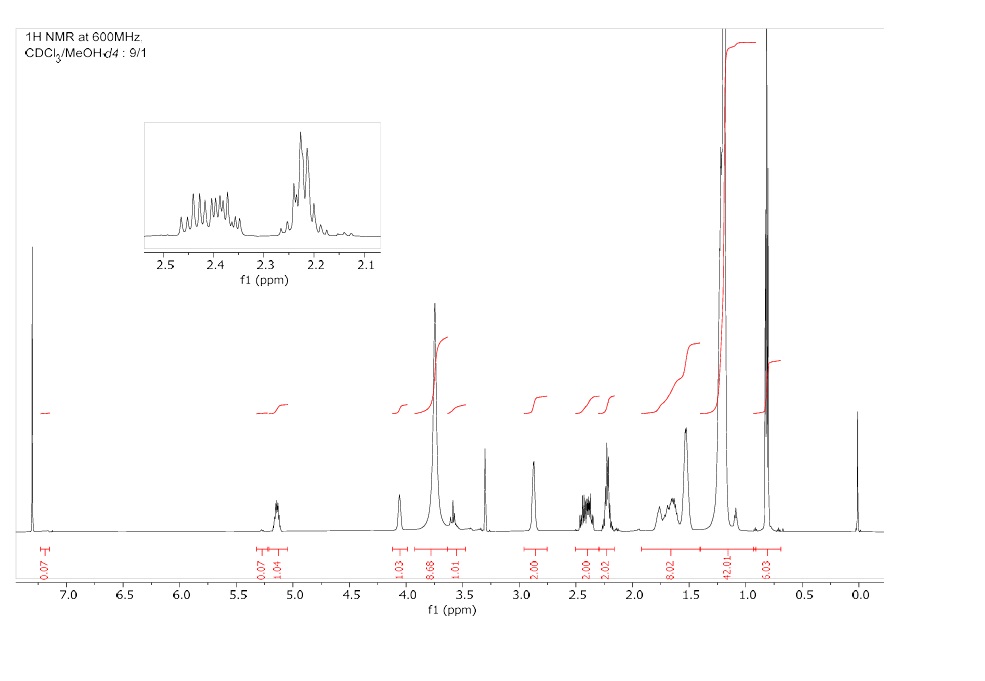

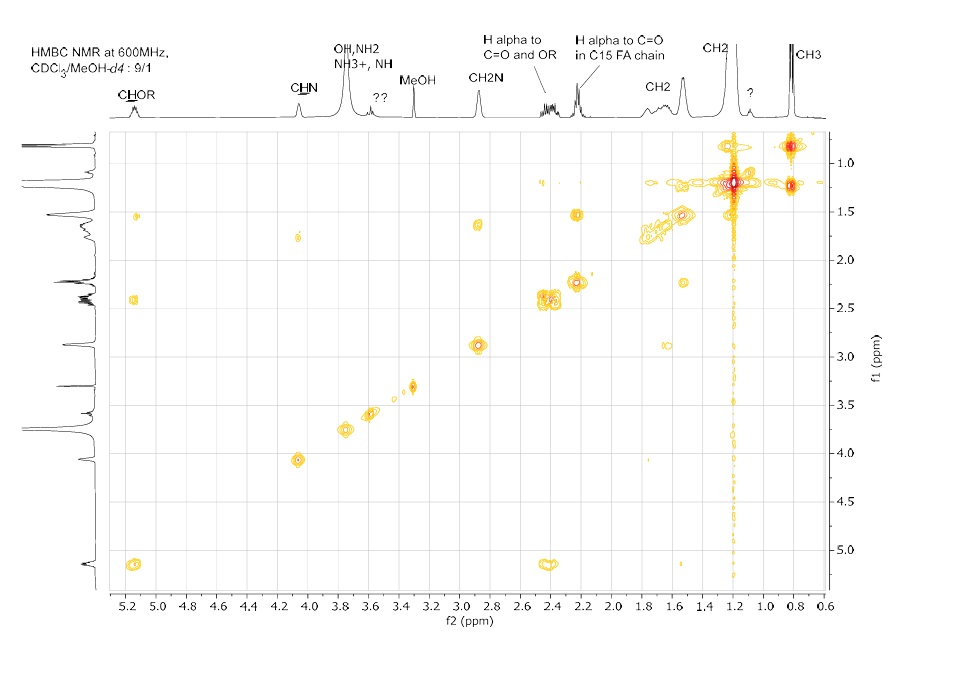

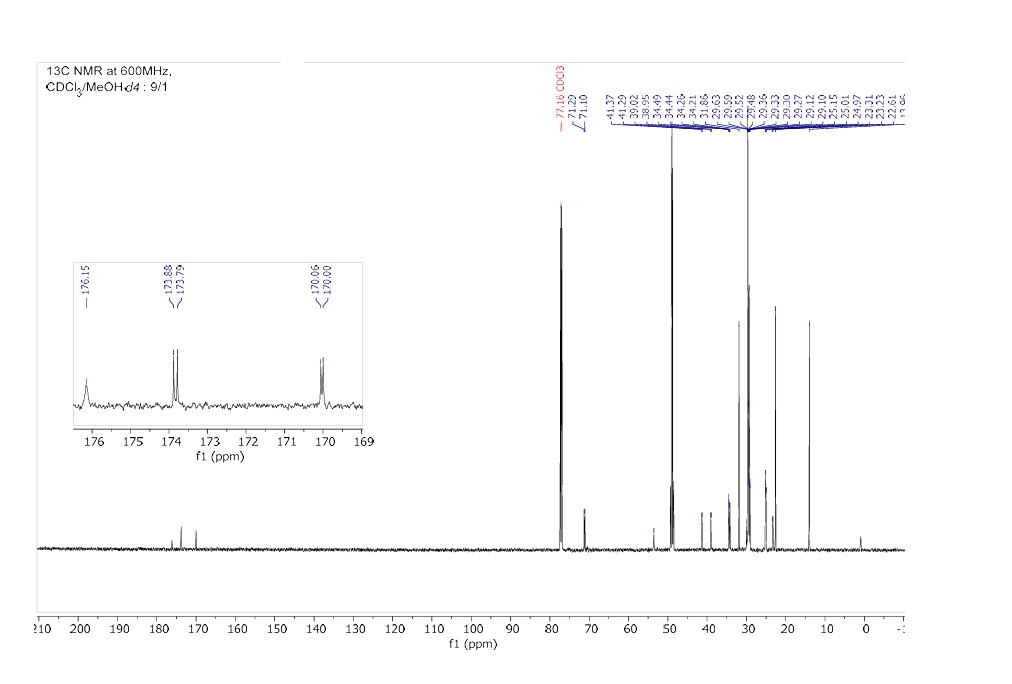

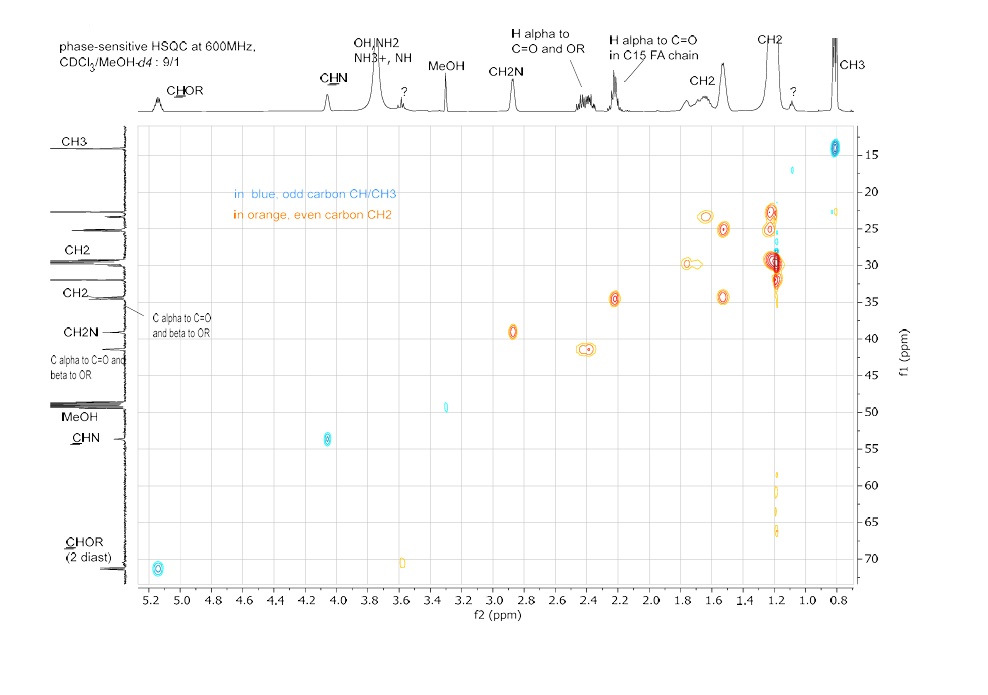

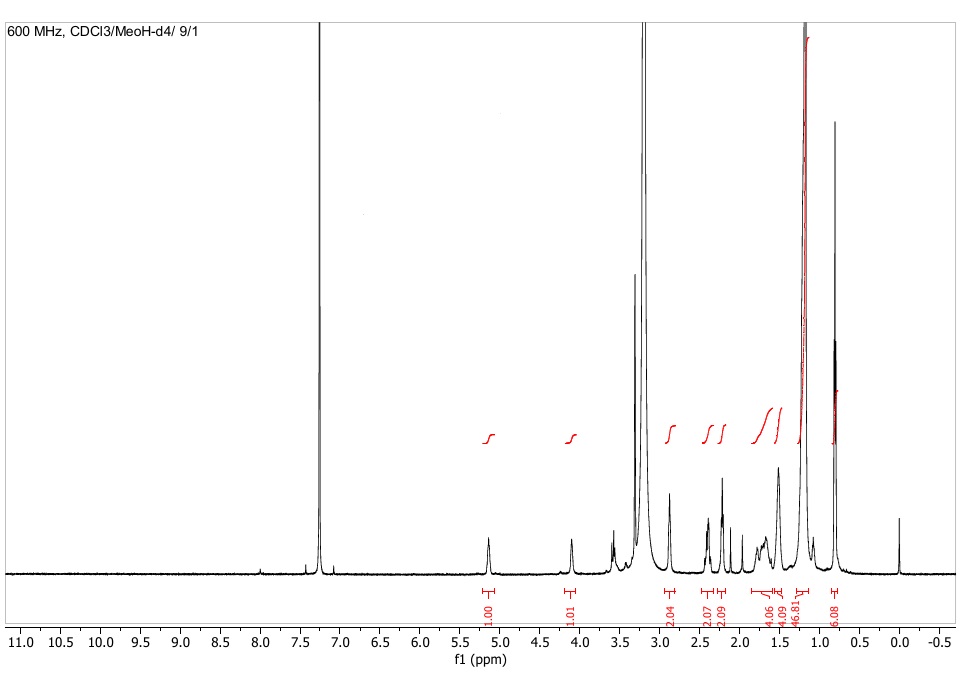

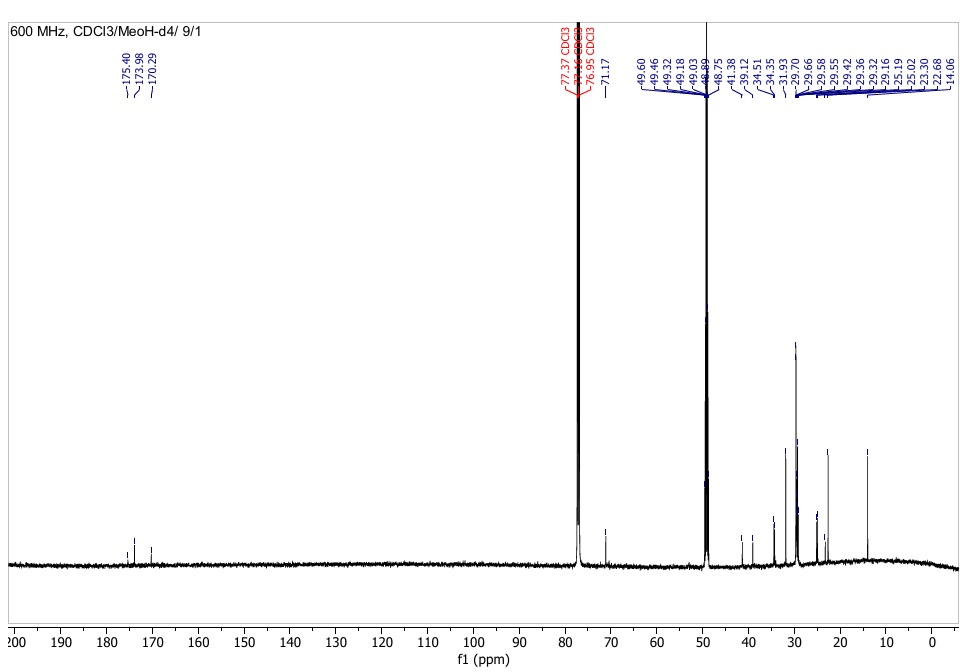


13C NMR, 125 MHZ, CDCl3/MeOH-d4/9/1


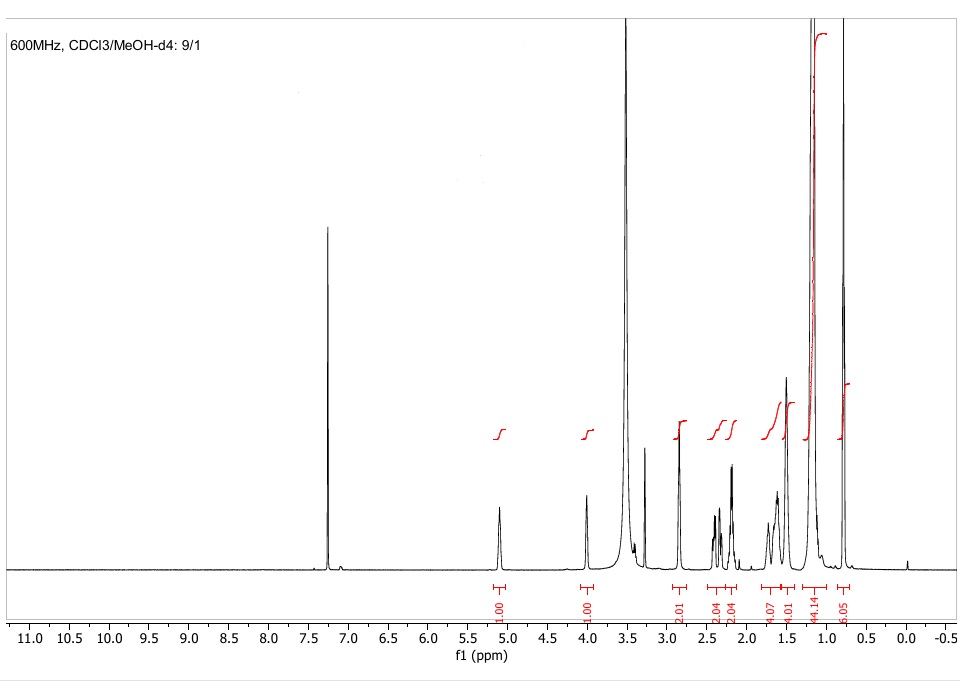

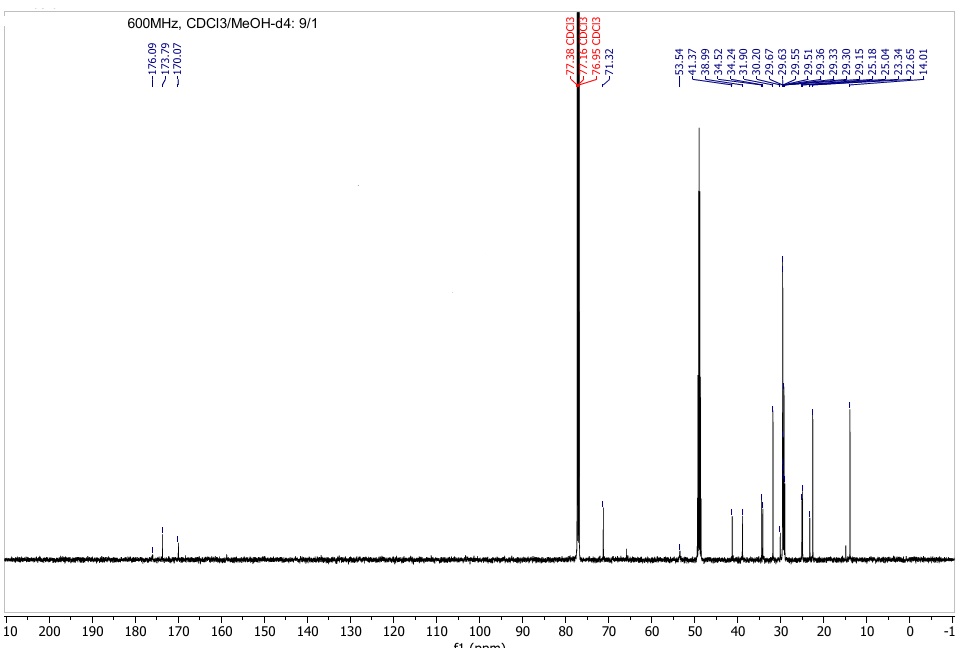


13C NMR, 125 MHZ, CDCl3/MeOH-d4/9/1

## Supplementary Tables

### Table S1. Non-targeted lipidomics MS and MS/MS parameters (Sciex X500 QTOF)

| Parameter | value | Information |
| --- | --- | --- |
| method duration | 25 min | source and gas parameters |
| Total scan time | 0.412 s |  |
| Estimated cycles | 3640 |  |
| Ion source gas 1 | 45 psi |  |
| Curtain gas | 30 psi |  |
| CAD gas | 7 |  |
| Temperature | 500 °C |  |
| TOF start mass | 65 Da | TOF MS parameters |
| TOF stop mass | 1000 Da |  |
| Spray voltage | (+/-) 4500 V |  |
| Accumulation time | 0.1 s |  |
| Declustering potential | (+/-) 50 V |  |
| DP spread | 0 V |  |
| Collision energy | (+/-) 5 V |  |
| CE spread | 0 V |  |
| Maximum candidate ions | 10 |  |
| Intensity threshold exceeds | 1000 cps |  |
| TOF MS/MS start mass | 50 | TOF MS/MS parameters |
| TOF MS/MS stop mass | 1000 |  |
| Declustering potential | (+/-) 80 |  |
| Collision energy | (+/-) 35 V |  |
| CE spread | 15 V |  |
| DP spread | 0 |  |
| Accumulation time | 0.025 s |  |

### Table S2. Targeted lipidomics MS and MS/MS parameters (Bruker maXis QTOF).

| Parameter | value | Information | MS/MS method | Mode | Chromatographic  method |
| --- | --- | --- | --- | --- | --- |
| method duration | 27-35 min | source and gas parameters | DDA | ESI(+) | HPLC RP and  HILIC |
| End plate offset | 500 V |  |  |  |  |
| Capillary voltage | 4500 V |  |  |  |  |
| Nebulizer gas | 2 bar |  |  |  |  |
| Dry gas flow | 10 L/min |  |  |  |  |
| Dry gas temperature | 200°C |  |  |  |  |
| TOF start mass | 50 Da | TOF MS parameters |  |  |  |
| TOF stop mass | 1500 Da |  |  |  |  |
| TOF MS/MS start mass | 50 Da | TOF MS/MS parameters |  |  |  |
| TOF MS/MS stop mass | 1500 Da |  |  |  |  |
| Scan rate | 5 Hz |  |  |  |  |
| Collision energy | 30 eV |  |  |  |  |
| method duration | 25 min | source and gas parameters | MRM | ESI  (+/-) | UHPLC RP  C18 HST |
| End plate offset | 500 V |  |  |  |  |
| Capillary voltage | 4500 V |  |  |  |  |
| Nebulizer gas | 2 bar |  |  |  |  |
| Dry gas flow | 4 L/min |  |  |  |  |
| Dry gas temperature | 200°C |  |  |  |  |
| TOF start mass | 50 Da | TOF MS parameters |  |  |  |
| TOF stop mass | 1500 Da |  |  |  |  |
| TOF MS/MS start mass | 50 Da | TOF MS/MS parameters |  |  |  |
| TOF MS/MS stop mass | 1500 Da |  |  |  |  |
| Scan rate | 5 Hz |  |  |  |  |
| Collision energy | 30 eV |  |  |  |  |
| method duration | 25 min | source and gas parameters | DDA | ESI(-) | UHPLC  RP C8 |
| End plate offset | 500 V |  |  |  |  |
| Capillary voltage | 4500 V |  |  |  |  |
| Nebulizer gas | 2 bar |  |  |  |  |
| Dry gas flow | 10 L/min |  |  |  |  |
| Dry gas temperature | 200°C |  |  |  |  |
| TOF start mass | 50 Da | TOF MS parameters |  |  |  |
| TOF stop mass | 1500 Da |  |  |  |  |
| TOF MS/MS start mass | 50 Da | TOF MS/MS parameters |  |  |  |
| TOF MS/MS stop mass | 1500 Da |  |  |  |  |
| Scan rate | 5 Hz |  |  |  |  |
| Collision energy | 20 eV |  |  |  |  |

### Table S3. Estimation of OL 16:0/15:0 concentration in the *A. muciniphila* cell pellet and colon content samples using authentic standard (compound 8).

| Compound | Concentration (µg/mL) |
| --- | --- |
| OL 16:0/15:0 isol. Fraction | 2.21 |
| OL 16:0/15:0 isol. Bacteria | 3.5 |
| OL 16:0/15:0 Colon | 0.18 |

### Table S4. Comparison of the chemical shifts (1H NMR in CDCl3, 298 K) in both compounds 11 and 12.

| 1H | **δ**ppm in 11 | **δ**ppm in 12 | **Δδ** = **δ**ppm (*S*)_mandel._ - **δ**ppm (*R*)_mandel._ |
| --- | --- | --- | --- |
|  | from | from |  |
|  | (*R*)-OAc-mandelic Acid | (*S*)-OAc-mandelic Acid |  |
| H2 | ABX system | 2 doublet of doublet |  |
| CH2 _alpha of C=O_ | υ_A_ = 2.52 | H_A_ = 2.70 | 0.18 |
|  | υ_B_ = 2.49 | H_B_ = 2.57 | 0.08 |
| H3 | Multiplet m | Multiplet m |  |
| CHO _beta of COOBn_ | 5.36-5.22 | 5.31- 5.23 | 0 |
| CH3 | triplet | triplet | 0 |
|  | 0.88 | 0.88 |  |
| CH2_benzyl_ | υ_Center_ = 4.81 | υ_Center_ = 5.09 | 0.28 |
|  | υ_A_ = 4.86 | υ_A_ = 5.12 | 0.26 |
|  | υ_B_ = 4.77 | υ_B_ = 5.06 | 0.29 |

### Table S5. Chiral chromatographic separation parameters of both enantiopure epimers 7.

| RT (min) | Area | Area Percent | Capacity | Compound |
| --- | --- | --- | --- | --- |
| 4.56 | 9549 | 49.75 | 0.55 | (*S*,*S* + *R*,*S*)-7 |
| 5.68 | 9643 | 50.25 | 0.93 |  |
| Sum | 19193 | 100 |  |  |
| 4.56 | 13163 | 100 | 0.55 | (*S*,*S*)-7 |
| Sum | 13163 | 100 |  |  |
| 4.56 | 109 | 0.97 | 0.93 | (*R*,*S*)-7 |
| 5.69 | 11141 | 99.03 |  |  |
| Sum | 11251 | 100 |  |  |

# References

1. Weiss AS, Burrichter AG, Durai Raj AC, von Strempel A, Meng C, Kleigrewe K, et al. In vitro interaction network of a synthetic gut bacterial community. ISME J. 2022;16(4):1095-1109. doi:10.1038/s41396-021-01153-z
2. Sillner N, Walker A, Koch W, Witting M, Schmitt-Kopplin P. Metformin impacts cecal bile acid profiles in mice. J Chromatogr B. 2018;1083:35-43. doi:10.1016/j.jchromb.2018.02.029
3. Selmi H, Walker A, Debarbieux L, Schmitt-Kopplin P. Improving the intestinal lipidome coverage in a gnotobiotic mouse model using UHPLC-MS-based approach through optimization of mobile phase modifiers and column selection. J Chromatogr B. 2024;1242:124188. doi:10.1016/j.jchromb.2024.124188
4. Fu X, Xu Z, Gawaz M, Lämmerhofer M. UHPLC-MS/MS method for chiral separation of 3-hydroxy fatty acids on amylose-based chiral stationary phase and its application for the enantioselective analysis in plasma and platelets. J Pharm Biomed Anal. 2023;223:115151. doi:10.1016/j.jpba.2022.115151
5. Chen D, Xu L, Yu Y, Mo Q, Qi X, Liu C. Triflylpyridinium Enables Rapid and Scalable Controlled Reduction of Carboxylic Acids to Aldehydes using Pinacolborane. Angewandte Chemie. 2023.
6. Chen D, Xu L, Ren B, Wang Z, Liu C. Triflylpyridinium as Coupling Reagent for Rapid Amide and Ester Synthesis. Org Lett. 2023;25(24):4571-4575. doi:10.1021/acs.orglett.3c01598
7. Chataigner I, Lebreton J, Durand D, Guingant A, Villiéras J. A new approach for the determination of the absolute configuration of secondary alcohols by 1H NMR with O-substituted mandelate derivatives. Tetrahedron Lett. 1998;39(13):1759-1762. doi:10.1016/S0040-4039(98)00059-8
8. Zhang Q, Linke V, Overmyer KA, Traeger LL, Kasahara K, Miller IJ, et al. Genetic mapping of microbial and host traits reveals production of immunomodulatory lipids by Akkermansia muciniphila in the murine gut. Nat Microbiol. 2023;8(3):424-440. doi:10.1038/s41564-023-01326-w
9. Gottlieb HE, Kotlyar V, Nudelman A. NMR Chemical Shifts of Common Laboratory Solvents as Trace Impurities. J Org Chem. 1997;62(21):7512-7515. doi:10.1021/jo971176v
10. Fautz E, Rosenfelder G, Grotjahn L. Iso-branched 2- and 3-hydroxy fatty acids as characteristic lipid constituents of some gliding bacteria. J Bacteriol. 1979;140(3):852-858. doi:10.1128/jb.140.3.852-858.1979
11. Natarajan S, Ibdah J. Role of 3-Hydroxy Fatty Acid-Induced Hepatic Lipotoxicity in Acute Fatty Liver of Pregnancy. Int J Mol Sci. 2018;19(1):322. doi:10.3390/ijms19010322
